# Time preferences and COVID-19 vaccination uptake

**DOI:** 10.1007/s10198-025-01801-7

**Published:** 2025-06-14

**Authors:** Arthur E. Attema, Marcello Antonini, Mesfin Genie, Aleksandra Torbica, Francesco Paolucci

**Affiliations:** 1 EsCHER, Erasmus School of Health Policy & Management, Erasmus University, P.O. Box 1738, Rotterdam, 3000 DR The Netherlands; 2 School of Medicine and Public Health, University of Newcastle, Callaghan, NSW 2308 Australia; 3 Department of Health Policy, London School of Economics and Political Science, London, UK; 4 Newcastle Business School, University of Newcastle, Newcastle, NSW 2300 Australia; 5 Health Economics Research Unit, Institute of Applied Health Sciences, University of Aberdeen, Aberdeen, AB25 2ZD UK; 6 Department of Social and Political Sciences, Bocconi University, 20136 Milano, Italy; 7 Department of Sociology and Business Law, University of Bologna, 40126 Bologna, Italy

**Keywords:** COVID-19, Present bias, Time preference, Vaccination

## Abstract

In the evolving COVID-19 endemic phase, the emergence of new variants and viruses may necessitate renewed efforts to control their spread. Understanding how individual preferences and attitudes influence vaccination behavior is crucial in preparation for this. This paper presents results from a multi-country online survey conducted in 22 countries between July 2022 and June 2023. The survey elicited individuals’ time preferences (discount rate and present bias) implementing choice list methodology and explored respondents’ behaviors and attitudes for vaccination using respectively revealed and stated preferences data through a Discrete Choice Experiment. Respondents were classified as vaccine accepters (one or more COVID-19 doses at the time of the data collection), hesitant (willing to get the vaccine if given conditions are met) and refuser (no intention to get the vaccine). Overall, we found no evidence of a present bias, but substantial variation in discount rate between respondents across different countries. Minimal differences between revealed and stated preferences data were found. Hesitant respondents revealed larger discount rates compared to the vaccinated respondents, indicating a stronger preference for immediate outcomes. On the contrary, refusers showed lower discount rates, indicating stronger preferences for future outcomes. The high discount rate of the hesitant group may result in a low weight given to the consequences of a future COVID-19 infection. Hence, policymakers could specifically target hesitant people to make the long-term advantages of COVID-19 vaccinations, in terms of reduced infection probability and lower disease severity in case of infection, more salient, or to focus more on the short-term benefits of vaccination, for instance by stressing the improved access it may bring to social events and the reduced chances of getting sick.

**Supplementary Information:**

The online version contains supplementary material available at 10.1007/s10198-025-01801-7.

## Introduction

In both health-related and financial decisions, the consideration of costs and consequences over time is imperative. These decisions often involve intertemporal tradeoffs, heavily influenced by the decision maker’s time preference, which is typically expressed by discount rates [1–3]. For instance, individuals with a high discount rate prioritize the present over the future and might be more inclined to engage in immediate gratification, such as smoking and drinking, neglecting their future well-being. In the context of preventive behavior, such as in the realm of vaccinations against potential future diseases, costs have mostly occurred in the short run, whilst benefits materialize much later [4]. As a result, individuals with a high discount rate tend to invest less in these preventive behaviors, as they give less weight to the future benefits relative to the more immediate costs. This underscores the significant role of time preferences in vaccination decisions [4–7]. The urgency to understand the influence of time preferences on vaccination behavior has been further heightened by the advent of the COVID-19 pandemic and the subsequent development of vaccines, aimed at mitigating the likelihood and severity of infections.

The study of vaccination uptake, especially concerning COVID-19, presents unique considerations distinct from other preventive activities. Vaccinations entail externalities, as the benefits extend not only to the vaccinated individuals but also to the broader community, potentially contributing to the achievement of herd immunity. However, challenges such as the rapid mutation of certain viruses, as seen in the case of COVID-19, may limit the attainment of herd immunity even with widespread vaccination [8]. Additionally, individuals with higher discount rates, i.e. giving less weight to future consequences, may not necessarily be more hesitant toward vaccination; instead, their impatience might prompt a preference for currently available imperfect vaccines, instead of waiting for better alternatives to emerge, if a vaccination allows immediate access to various activities or reduced illness severity post-infection in the short term [9]. Furthermore, the possibility of vaccine side effects, particularly long-term effects that are often unknown in newly developed vaccines like those for COVID-19, underscores the relevance of considering risk and ambiguity attitudes in vaccination decisions. For the case of COVID-19, we expect that present bias predominantly leads to vaccine hesitancy — that is, individuals intend to get vaccinated eventually but delay it because immediate costs (such as effort, discomfort, or side effects) are given disproportionate weight compared to future benefits. Conversely, for individuals with a high discount rate, we expect this to result in a lower intention to get vaccinated altogether, potentially leading to outright refusal. While vaccination can also offer immediate benefits (e.g., faster access to activities or reduced short-term infection risk), these benefits may often be less salient or perceived as less certain than the immediate inconveniences associated with getting vaccinated. Similar to the effect of time preference, the effect of risk aversion on the vaccination decision is a priori not evident, as risk averse individuals will dislike the risk of side-effects but also the risk of severe disease if getting infected without being vaccinated [10, 11].

Numerous prior studies have examined the link between time preference and vaccination behavior, including those for influenza, Hepatitis B, Polio, and COVID-19 [6, 12–14]. Many of these studies have established a significant negative association between discount rates and vaccination attitudes, which is typically explained by the immediate costs of a vaccination and the long-term benefits of the vaccine’s protection. This paper presents the findings of a comprehensive multi-country empirical study that expands the current research trajectory in several crucial ways. Firstly, we investigate time preferences by disentangling the roles of present bias and discount rate in COVID-19 vaccination behavior implementing two intertemporal choice tasks. This allows recognizing these two constructs of time preference as distinct entities with varied properties. Specifically, the present bias captures the amount to which someone favors the present to the future irrespective of how distant this future is, and can result in impulsivity [6, 12–15], while the discount rate captures the amount of discounting per period which reveals individual impatience. These two concepts may justify different policy measures to encourage healthy behavior. For instance, a present bias (β) may be reduced by commitment devices (e.g., scheduling vaccination appointments in advance) or by framing vaccination to emphasize immediate benefits (e.g., ‘protect yourself today against getting sick’ or ‘regain access to social activities more quickly’). This distinction is important, because a present bias may result in people not being able to stick to their plans, causing preference reversals that reduce long-term welfare, while a high discount rate reflects a low weight given to the future, but does not conflict with rational economic preferences, and hence, does not per se reduce welfare. As has been found in other applications, it may be that only one of these two constructs is related to a particular trait or behavior, but the other is not, or in the opposite direction [16], for example because the immediacy effect is more influential for one type of behavior, such as getting a vaccine, than another. For instance, Madsen and Kjaer [17] found a significant positive relation between discount rates and body mass index (BMI), but an unexpected negative relation between present bias and BMI. Furthermore, some studies found both variables to be related to smoking behavior and obesity, in the predicted direction [18–20]. Hence, to formulate more effective health promotion policies, it is essential to disentangle them. For the case of COVID-19, we expect a present bias to lead to postponement of vaccinations, i.e. people wanting to eventually get a vaccine but delaying it because the current costs of getting it get disproportionally more weight than the discounted benefits, while for a high discount rate we expect this leads to a lower intention to get the vaccine.

Secondly, this study employs the choice list methodology to quantify time preferences, a method extensively used in health economic research [21–24]. Notably, this research stands as the first to investigate the relationship between time preference and vaccination attitudes/ behavior within an extensive worldwide sample of 50,242 respondents collected through an online survey [25, 26]. In addition to time and risk preferences, the study also collected a range of other variables, including socioeconomic characteristics, such as age, gender, educational and income levels, and whether respondents have children. The richness of this information facilitates a comprehensive analysis of the phenomena under investigation.

Thirdly, this study crucially differentiates among three categories of respondents: vaccine accepters, hesitant respondents, and outright vaccine refusers. The distinction between hesitant and outright refusers, along with terminological precision to identify the problem, is pivotal for delving into the underlying reasons behind the observed delay in vaccine acceptance [27]. Specifically, outright refusers are respondents who categorically reject vaccination, independently of objective data on safety and effectiveness, e.g. because they perceive vaccines as dangerous. On the other hand, vaccine hesitant individuals are likely to be open to accepting the vaccine if their concerns are addressed. Targeting this group is particularly relevant for policy interventions aimed at augmenting vaccination coverage.

Lastly, the present study undertakes a comparative analysis between outcomes derived from revealed vaccination preferences (RP) and stated vaccination preferences (SP). The former refers to the current COVID-19 vaccination status at the time of the data collection. While the latter derives from data collected via a Discrete Choice Experiment (DCE), wherein respondents were asked to state their willingness to undergo vaccination against a future pandemic similar to COVID by navigating through twelve hypothetical choice scenarios. DCEs are commonly used in the literature for studying preferences of consumers in health and other settings [28, 29]. The distinction between RP and SP decisions is relevant to investigate vaccination attitudes. The current vaccination status may not fully capture respondents’ preferences in certain countries, reflecting mandates or government-implemented public policies. Similarly, relying solely on SP data may introduce hypothetical bias, where individuals may exhibit different behavior in a hypothetical setting compared to real decisions [28–34]. Consequently, the inclusion of both RP and SP information serves also as a robustness check for our results. Furthermore, incorporating SP data holds an additional advantage as it pertains to hypothetical vaccination programs. The insights derived can be instrumental in formulating effective long-term vaccination and promotion policies, not only for COVID-19 but also for other diseases.

## Related literature

Numerous recent studies have investigated COVID-19-related preferences, particularly the link between time preferences and the demand for COVID-19 vaccines. In this section, we review these studies, starting with those that demonstrated a negative association between time preferences and vaccination.

First, Blondel et al. [35] explored the French context, incorporating risk, time, and altruism into their analysis of vaccination intentions. They reported that higher discount rates could reduce vaccine acceptance rates among women and the elderly. In the United States, Strickland et al. [13] used the monetary choice questionnaire [33] to assess discounting, and identified a negative relationship between increased discounting and the likelihood of vaccination. Similarly, Halilova et al. [36] explored time preferences among non-vaccinated individuals across 13 countries in Europe, North America and Australasia. Employing the framework established by Green and Myerson [37], they utilized 42 choices to measure discounting. Their results demonstrated steeper delay discounting among the unvaccinated, with this factor independently predicting vaccination status, even after accounting for demographics and mental health. In Canada, Hudson et al. [38] analyzed predictors of vaccine hesitancy and COVID-19 mitigation behaviors. They utilized the 5-trial adjusting delay discounting task of Koffarnus and Bickel [39] to estimate time preferences. Their study revealed that individuals displaying less discounting and a more future-oriented perspective were more likely to be double-vaccinated against COVID-19. In Japan, Okamoto et al. [40] conducted an online study encompassing 5000 adults, using a single question to gauge time preferences by assessing the compensation individuals required for delaying hypothetical funds for one year. Their study indicated a positive association between time preferences and vaccine hesitancy, i.e. respondents with a higher discount rate were less inclined to be vaccinated.

We found one study reporting a significant positive association between discounting and vaccination. Guillon et al. [9] used a validated self-stated preference measure of patience [41], and the Convex Time Budget method [42] to estimate non-constant discounting in France during the late summer 2021. They found a positive association between individuals’ discounting and their COVID-19 vaccine uptake. This study is the closest to our analysis in that it studies the relationship between time preferences and COVID-19 vaccination using a quasi-hyperbolic discounting framework, and it highlights the importance to consider discounting as a key lever to design effective vaccination campaigns. The study differs from the current analysis in that they used other time preference measurement tasks, one incentivized and one not incentivized, for only French respondents. Additionally, they categorized respondents as time-consistent, present-biased and future-biased, but did not estimate the parameters of the discount function.

Finally, some other studies were inconclusive, or reported contradictory findings for the present bias and the discount rate. First, Yue et al. [43] explored the timing of vaccination and the associated role of time preference through a DCE in Hong Kong. They employed a rating task allowing respondents to indicate their preferred timing for receiving vaccine injections. They found that the desire for early vaccination was not significantly affected by the perceived severity of the pandemic or personal risk, while those individuals confident in their preventive measures opted for delayed vaccination. Using the same task as [40], Okubo et al. [44], in a separate large-scale survey in Japan involving approximately 10,000 participants, found that time preferences were not a decisive factor in vaccination decisions. Prior to COVID-19, findings from a representative German sample were also reported by Nuscheler and Roeder [45]. They developed a model of individual vaccination decisions for seasonal influenza in 2010-11, incorporating risk aversion and information about the flu and the flu shot. They measured the discounting parameters through two time preference questions, using matching questions and time horizons of 1 year, 10 years and 11 years. Although their theoretical model suggested a negative association between discounting and vaccination decisions, their econometric analysis yielded mixed evidence. Notably, gender played a significant role, with men less (more) likely to align with the theoretical model’s hypothesis for the discount rate (future bias), while no significant effect of time preferences was observed in women’s vaccination decisions.

## Method

The discounted utility model is the standard vehicle to study intertemporal preferences and can be evaluated by:

1 $${\rm{DU}}({\rm{x}},{\rm{t}}) = {\rm{D}}({\rm{t}}){\rm{U}}({\rm{x}}),$$

where DU(x, t) is the discounted utility of outcome x received at time point t, D(t) is the discount function, which is decreasing in t, and U(x) is the utility of x. The most common forms of D(t) are the constant (or exponential) discounting model [46] and the quasi-hyperbolic (or beta-delta) discounting model [47, 48]. The quasi-hyperbolic model is given by D(t)= β*δ^t^, with the constant discounting model being a special case for β = 1, and β < 1 (> 1) indicating a present (future) bias. We estimate both these models in our study.

To elicit intertemporal preferences and gather information on vaccination behaviors and attitudes, we devised a comprehensive online survey across multiple countries. The survey incorporated a choice list methodology and DCE. In the following subsections, we provide a detailed explanation of the methodology and the design employed to capture the two fundamental dimensions of our study.

### Time preferences

We used the choice list methodology to elicit intertemporal preferences while disentangling two constructs: present bias and discount rate. To estimate the two-parameter quasi-hyperbolic discounting model, we implemented two hypothetical choice lists: one including a present option and a future option, and one including two future options. Table A1 in the Supplementary Material shows the stimuli of these choice lists. To maintain consistency across countries, the monetary values were converted using the purchasing power parities (PPPs) conversion rate retrieved from the OECD database for countries with a rate of conversion above (below) 1.5 (0.5).

With these choice lists we could test for the occurrence of a present bias by comparing the switching rows. Specifically, because the only difference between the two lists is that the delay has increased by three months for both options, constant discounters are predicted to switch in the same row twice. This is caused by the stationarity axiom underlying the constant discounting model, stating that only the time difference between two options is relevant, whereas a common delay should not affect preferences. Instead, a present bias (i.e., β < 1) will on average result in a lower switching row for Choice list 2 than for Choice list 1 in Table A1 in Supplementary Material A, because the present bias only affects Choice list 1, because β is cancelled out in Choice list 2, due to both options being realized in the future.

We also estimated the parameters of the β-δ model by first computing the discount rate from Choice list 2 and then using this estimate to solve for β using the indifference of Choice list 1.^1^

### Vaccine attitudes and behaviors

When considering vaccine hesitancy, clarifying the distinction between refusers and hesitant individuals is key. Vaccine refusing individuals are defined as those who would never take the vaccine independently of the type of vaccine, its safety and effectiveness. On the contrary, vaccine hesitant respondents are those who are open to get the vaccine if given conditions are met. From a policy perspective, this is the most important group to target to increase population coverage and tend towards herd immunity.

#### Vaccination behaviors (RP)

Our rich dataset allowed us to create a more general classification of respondents’ vaccination attitudes looking at their revealed vaccination status. Specifically, we asked respondents for their current COVID-19 vaccination status at the time of the survey. Respondents were presented with a range of options, including having already received the first dose and awaiting the second, planning to get vaccinated, or not intending to receive the vaccine. The survey question and corresponding response options are detailed in the Supplementary Material. Following the terminology of vaccine hesitancy proposed by Bedford et al. [24], we identified four categories separating those who face logistic issues to get the vaccines or that deliberately reject the vaccination from those *whose deliberations demonstrate something akin to indecision*. Specifically: (a) vaccinated (received at least one dose), (b) hesitant (those who did not get any dose and would get the vaccine only when they are sure it is effective or when they know more about the potential side effects), (c) vaccine refusers (those who did not receive any dose and have no intention to get the vaccine), and (d) finally those who cannot access vaccines for (i) medical reasons or (ii) accessibility issues (financial access or geographical access).

#### Vaccination attitudes (SP)

DCEs present participants with a series of hypothetical alternatives that resemble real-life scenarios and ask them to select their preferred option. The DCE included seven characteristics describing different vaccination programs, including features of both the vaccine and societal restrictions (see Supplementary Material). The attributes and levels were selected following best practices indicated in the literature [49]. Attributes and levels were combined into pairwise choice tasks using a D-efficient design [50]. The resulting 36 choice tasks were blocked into three versions to minimize the cognitive burden for the respondents. The design was optimized for the estimation of a multinomial logit (MNL) model and was created using NGENE software (ChoiceMetrics [51]). A set of three candidate designs were created using a modified Fedorov algorithm combined with a swapping algorithm [52]. We selected the design with the lowest D-error and lowest Pearson correlations between characteristics.

Respondents were asked to choose between two vaccination programs. The order of the 12 tasks was randomized for each participant to minimize ordering effects [53]. We adopted a dual format. At first, respondents were asked to indicate their preferred option: “Which option would you choose?”. Second, respondents were asked to choose between the selected vaccination program or the no vaccination scenario: “Suppose you now can choose not to be vaccinated. Which option would you choose?”. The subsequent follow-up question with an optout option was included to mimic real-world scenarios and account for vaccine refusers or hesitant respondents. Additionally, as respondents learn from answering forced-choice tasks, a dual response design might result in higher data quality than offering a direct opt-out option [54].

By calculating the number of times that respondents have chosen the optout option across the twelve choice tasks, we can create an ordinal variable that displays vaccine uptake intentions based on the SP data $\:{Y}_{n,t}\in\:\:\left[0,\frac{1}{12},\dots\:1\right]$ [55]. Following Schwarzinger [56] and Wang [57], refusers are identified as those that opted out in all the choice tasks (i.e.,, $\:{\sum\:}_{t=1}^{12}\frac{0}{12}$). Hesitant respondents are identified as those who chose at least one vaccination option. The lower the number of vaccination choices, the more pronounced is the hesitancy of the individual. These respondents will accept vaccination only if given conditions (vaccination and social restrictions attributes) are met. To match the three categories that we identified in the RP data, we categorize respondents with 10 or more vaccine choices as *vaccine accepters*. We use a multinomial logistic regression to investigate the role of time preference on the probability of accepting the vaccination option, controlling for risk aversion.

### Analysis

We report descriptive statistics to show the distribution of respondents in the three categories for both RP and SP data. Similarly, we report the average value of the discount rates and the β’s at the country level. A Wilcoxon signed-rank test was conducted at the country level to determine whether the distribution of impulsivity differed from 1 across vaccination status, while t-tests of means were performed at the country level to assess whether impulsivity and impatience differed by vaccination status (see Supplementary Material for results).

To examine the relationship between discount rates and present bias on the one hand, and vaccination attitudes on the other hand, we employ multinomial logistic regressions, both with and without country fixed effects. We commence with simple regressions that include only the discount rate and present bias variables. Subsequently, we augment the model with a set of demographic control variables and a covariate accounting for the inclination to take health-related risks. The latter variable was determined using the direct method proposed by Yang et al. [58].

The multinomial logistic regression is of the following form:

2 $$\eqalign{& In\left( {{{P\left( {refuser} \right)} \over {P\left( {accepters} \right)}}} \right) = {\alpha _{10}} + {\beta _{11}}rho2 + {\beta _{12}}beta +   \cr & {\beta _{13}}Riskhealth + {\beta _{14}}female + {\beta _{15}}age + {\beta _{16}}ag{e^2}  \cr &  + {\beta _{17}}bachelor + {\beta _{18}}highincome + {\beta _{19}}children + 1 \cr} $$

3 $$\eqalign{& In\left( {{{P\left( {{\rm{hesitant}}} \right)} \over {P\left( {{\rm{accepters}}} \right)}}} \right) = {\alpha _{20}} + {\beta _{21}}\rm{rho2} + {\beta _{22}}\rm{beta} +   \cr & {\beta _{23}}\rm{Riskhealth} + {\beta _{24}}\rm{female} + {\beta _{25}}\rm{age} +   \cr & {\beta _{26}}\rm{ag{e^2}} + {\beta _{27}}bachelor +   \cr & {\beta _{28}}\rm{highincome} + {\beta _{29}}\rm{children} + {\varepsilon _2}n \cr} $$.

In these expressions, *rho2* represents the discount rate in the quasi-hyperbolic discounting model, and *beta* the present bias. We repeated this regression while setting *beta* equal to 1, i.e., testing the constant discounting model, where the average discount rate $\:\widehat{\rho\:}$ replaces the discount rate *(rho2)* and present bias, and we utilize the Accepters group as the baseline category. Here, α represents the constant term, β_n_ signifies the estimated coefficient, and ε denotes the error term. Specifically, the variable ‘female’ is a binary variable taking the value of 1 if the respondent is female and 0 otherwise. ‘Age’ is a continuous variable, ‘bachelor’ is a binary variable taking the value of 1 if the respondent holds at least a bachelor’s degree, and ‘high income’ is a binary variable taking the value of 1 if the respondent’s income is at least twice the median household income level in their respective country. Lastly, ‘children’ is a covariate taking the value of 1 if the respondent has one or more children and 0 otherwise. Consistent with the existing literature [27], we excluded respondents who did not receive the vaccine due to medical or accessibility reasons from the regression analysis (*N* = 1,015: medical problems = 764; accessibility issues = 251).

### Countries involved, sample size and recruitment

50,242 adult respondents (≥ 18 years old) from 22 countries across 6 different continents anonymously completed the survey (see Table 1). Participants received a fixed monetary compensation for their participation, independent of their responses in the time preference task. Countries were chosen to provide variation on the overall impact of COVID-19, including epidemiological outcomes and policy responses as well as different cultural, socioeconomic and demographic backgrounds to maximize the generalizability of the multi-country comparison. An additional criterion for country inclusion was the presence of researchers in our team who are familiar with the country context, the language, and the COVID-19 experience in each country, or our access to such individuals through our networks. A specialized market research company (DemetraOpinioni.net) circulated the online survey adopting Computer Assisted Web Interviewing (CAWI) methodology. Quota sampling was based on age, gender, and location to ensure the demographic representativeness of countries populations. The sample size in each nation was dependent on that nation’s population. Countries with a population of more than 15 million people had a sample size of 3,000 respondents; those with a population of between 5.1 million and 15 million had 1,500 respondents; and those with a population of less than 5 million had 1,000 respondents. These thresholds were established by the research team to enhance the representation of the samples collected in each country by maximizing respondents’ heterogeneity within the constraints of the available budget. Table 1 reports the total number of respondents in each country. More information about the sampling procedure is documented in the Supplementary Material and in Antonini et al. [25].

**Table 1:** Countries included in the project and sample size

Country	Total respondents
Australia	3,004
Brazil	3,001
Chile	3,004
Croatia	1,062
France	3,165
India	3,128
Israel	1,513
Italy	3,001
Latvia	1,109
Norway	1,033
Lithuania	1,010
Russia	3,010
Singapore	1,002
Slovakia	1,009
Slovenia	1,061
South Africa	3,002
South Korea	3,000
Spain	3,266
Sweden	1,503
Turkey	3,086
UK	3,115
USA	3,185
Total	50,242

## Results

### Descriptive statistics

Around 13% of our sample did not get any vaccine at the time of survey. Of these, 8.7% were outright refusers, while 4.7% remained hesitant about the vaccines and had not yet been vaccinated for that reason. A small fraction of the sample (0.5%) did not receive the vaccine due to accessibility problems. Similarly, a small proportion of the respondents (1.5%) did not get the vaccine for medical reasons. (Although these respondents ‘deliberately’ did not get vaccinated, it is customary to regard medical exemptions to vaccination as distinct from vaccine refusal.)

Table 2 presents summary statistics categorized by vaccination status within each country, for both revealed and stated vaccination statuses. Differences are evident in the percentages across groups, with a significantly larger proportion of refusers and hesitant individuals in the stated preference data obtained from the DCE. In certain instances, these distinctions are substantial, particularly in the Israeli, Latvian, and Lithuanian samples, where the disparities between the two sets of figures exceed 30% points. These findings highlight the importance of considering both datasets to mitigate potential biases stemming from mandatory vaccination policies and the presence of hypothetical bias. It is worth mentioning that the percentage of refusers in India is minimal for both SP and RP data, which is also the case for revealed vaccination status in Brazil, Chile, and Singapore.

**Table 2:** Vaccination status by country (revealed and stated vaccination status) (%)

	Refuser			Hesitant			Accepters		
Country	Revealed	Stated	Diff.	Revealed	Stated	Diff.	Revealed	Stated	Diff.
Australia	6	10	-4	2	18	-16	92	72	20
Brazil	2	5	-3	2	9	-7	97	86	11
Chile	2	12	-10	1	12	-11	96	76	20
Croatia	21	33	-12	10	23	-13	69	44	25
France	9	21	-12	4	26	-22	87	54	33
India	1	2	-1	2	17	-15	97	81	16
Israel	6	40	-34	3	44	-41	91	16	75
Italy	5	14	-9	1	21	-20	94	65	29
Latvia	19	46	-27	7	20	-13	75	34	41
Lithuania	17	40	-23	6	21	-15	77	40	37
Norway	5	8	-3	2	23	-21	93	68	25
Russia	23	34	-11	21	23	-2	55	43	12
Singapore	1	11	-10	1	21	-20	98	68	30
Slovakia	21	29	-8	8	18	-10	71	52	19
Slovenia	22	34	-12	9	20	-11	69	46	23
South Africa	16	23	-7	12	18	-6	73	59	14
South Korea	4	11	-7	4	30	-26	93	59	34
Spain	5	11	-6	2	17	-15	94	71	23
Sweden	8	9	-1	4	36	-32	88	55	33
Turkey	8	18	-10	3	21	-18	89	61	28
UK	7	11	-4	3	20	-17	90	69	21
USA	15	17	-2	6	18	-12	78	65	13
Total	9	17	-8	5	21	-16	86	62	24

In relation to time preferences detailed in Table 3, we observe the highest discount rates in India, Turkey, the USA, and South Africa, whereas the lowest levels of impatience are evident in Sweden, Norway, Spain, and Chile.

In the context of the hyperbolic discounting model, the mean estimates as reported in Table 3 point toward a future bias (i.e., β > 1) rather than a present bias (β < 1) for most countries. However, the median value for β equals 1 in all countries and we did not find statistically significant evidence that they were higher than 1. Across areas, statistically significantly greater patience is observed among Western European respondents compared to those from Anglo-Saxon and Emerging countries. However, no statistically significant differences are observed between Western European respondents and those from Eastern European or East Asian countries (see Figure A1 in the Supplementary Material).

**Table 3:** Time preferences– descriptive statistics

	Constant discounting			Hyperbolic Discounting								
**Area/group**	Discount rate (average list 1 & 2)			Discount rate choice list 2			Beta			Discount rate choice list 1		
**Anglo-Saxon**	Median	Mean	SD	Median	Mean	SD	Median	Mean	SD	Median	Mean	SD
Australia	0.19	0.39	0.43	0.18	0.38	0.44	1.00	1.62	4.06	0.21	0.41	0.19
UK	0.19	0.36	0.39	0.18	0.35	0.41	1.00	1.77	4.43	0.18	0.37	0.19
USA	0.37	0.51	0.45	0.25	0.5	0.47	1.00	1.91	4.85	0.31	0.52	0.37
**East Asia**												
Singapore	0.2	0.4	0.43	0.21	0.39	0.45	1.00	1.78	4.26	0.18	0.4	0.2
South Korea	0.23	0.36	0.38	0.18	0.33	0.39	1.00	1.46	3.62	0.25	0.4	0.23
**Eastern Europe**												
Croatia	0.13	0.33	0.4	0.13	0.34	0.43	1.00	2.25	5.91	0.11	0.33	0.13
Latvia	0.19	0.37	0.4	0.18	0.38	0.45	1.00	2.49	6.08	0.16	0.35	0.19
Lithuania	0.13	0.31	0.39	0.11	0.33	0.42	1.00	2.26	5.75	0.08	0.3	0.13
Slovakia	0.15	0.32	0.39	0.16	0.33	0.41	1.00	2.15	5.61	0.11	0.32	0.15
Slovenia	0.13	0.32	0.4	0.13	0.32	0.42	1.00	1.96	4.97	0.08	0.32	0.13
**Western Europe**												
France	0.13	0.36	0.43	0.11	0.36	0.45	1.00	1.92	4.98	0.08	0.37	0.13
Israel	0.18	0.39	0.43	0.13	0.38	0.45	1.00	1.75	4.34	0.13	0.39	0.18
Italy	0.13	0.31	0.4	0.13	0.32	0.42	1.00	1.89	4.89	0.08	0.31	0.13
Norway	0.08	0.29	0.39	0.06	0.28	0.41	1.00	1.63	3.73	0.06	0.29	0.08
Spain	0.08	0.28	0.4	0.08	0.29	0.42	1.00	2.02	5.3	0.04	0.27	0.08
Sweden	0.1	0.31	0.4	0.08	0.3	0.41	1.00	1.64	3.98	0.08	0.32	0.1
**Emerging**												
Brazil	0.18	0.39	0.43	0.18	0.41	0.47	1.00	2.49	6.03	0.08	0.37	0.18
Chile	0.05	0.26	0.38	0.04	0.3	0.45	1.00	3.13	7.71	0.03	0.23	0.05
India	0.64	0.63	0.47	0.5	0.61	0.49	1.00	2.05	5.18	0.58	0.64	0.64
Russia	0.16	0.34	0.4	0.13	0.34	0.43	1.00	1.77	4.43	0.11	0.34	0.16
South Africa	0.25	0.41	0.42	0.21	0.42	0.46	1.00	2.37	5.92	0.18	0.4	0.25
Turkey	0.54	0.6	0.44	0.5	0.61	0.47	1.00	2.32	5.9	0.43	0.6	0.54

**Fig. 1:**
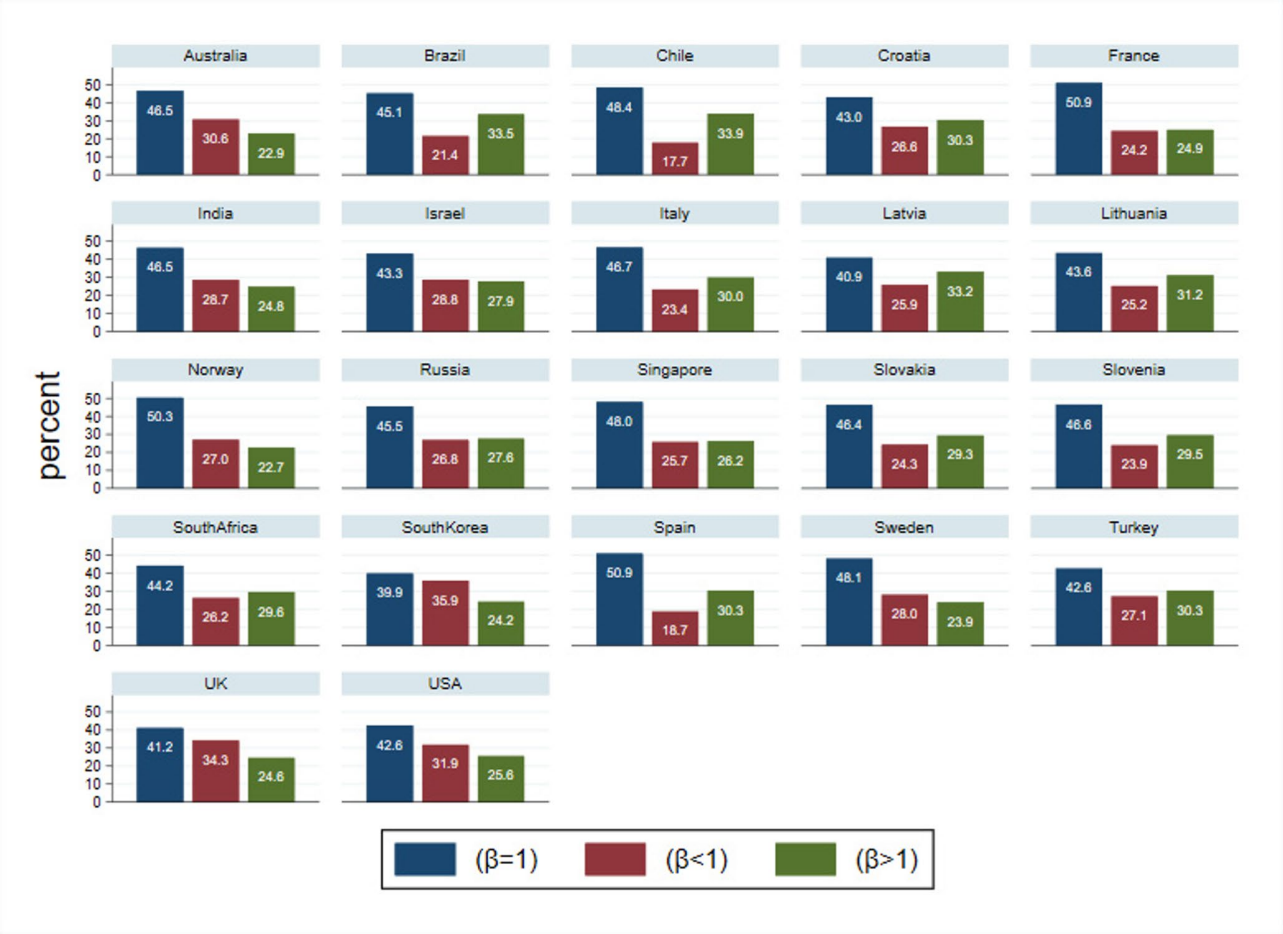
Beta distribution by country

To gain more insights on the presence of present, constant, or future bias, we reported the distribution of the respondents in Fig. 1. In all countries, the majority of the respondents reports β = 1. Differences arise only in terms of the percentages of present and future biases, with the latter being relatively more visible across countries. To test if these proportions vary across countries, we ran a chi-square test of independence, which revealed significant variation in the future bias proportion across countries (χ² = 295.20, *p* < 0.05). The Wilcoxon signed rank tests confirmed these findings. For all countries and vaccination status, results were unvaried (i.e., constant discounting was the most reported). Only a few countries reported diverging results. In Israel, the hesitant group showed a larger proportion of respondents reporting present bias (44% of the sample). The refusers among the South Korean respondents reported the majority of people with a β < 1 and β > 1 (38% and 34% of the sample, respectively). Results were confirmed when considering the stated vaccination status.

Looking at the results of our study in more general terms, we found mixed evidence across countries and compared to the previous literature. A comparison between our estimates of risk and time preferences and the validated survey conducted by Falk et al. [59] reveals intriguing insights. For instance, Falk et al. observed that populations of European ancestry tend to display greater patience, particularly in Scandinavian countries. Our data corroborates these findings for Western European populations, particularly among Scandinavian respondents. However, we observe significantly higher impatience among respondents in what we categorize as Anglo-Saxon countries. Similarly, our data confirms that East and South Asian countries, including India, Singapore, and South Korea, tend to report relatively higher levels of impatience, with the only “Confucian country” in our sample (South Korea) reporting lower levels. Falk et al. [59] identified relatively high levels of impatience in South American countries, and our data also provides mixed evidence, with respondents from Brazil displaying higher levels of impatience relative to the mean of the full set of countries, while Chile reports very low levels of impatience.

Additionally, we note a convergence in risk preferences for Anglo-Saxon and Eastern European countries, alongside distinctive patterns observed for African and Latin American countries. Moreover, our analysis of time preferences highlights a parallel trend for Scandinavian and East and South Asian countries, juxtaposed with diverging results for Anglo-Saxon counties. Nevertheless, it is important to consider that Falk et al. [59] employed distinct elicitation methods, introducing a potential source of variation in the comparative analysis. Additionally, their study did not encompass the exact same set of countries as our current investigation, thus limiting the direct comparability between the two studies.

Furthermore, an analysis of the global elicitation of time preferences conducted by Wang et al. [60] revealed similar trends, with respondents from Northern and Western European countries demonstrating the highest levels of patience, followed by (from most to least patient) Anglo-Saxon, Asian, Middle Eastern, Eastern European, Latin American, Southern European, and African countries. Notably, the findings in Wang et al.‘s study indicate a more pronounced manifestation of present bias, potentially attributed to their utilization of a matching procedure, exclusive comparison between present and future rewards, and longer delay periods of up to 10 years. These methodological disparities emphasize the nuanced nature of time preference elicitation and the necessity for careful consideration of the specific methodologies employed when interpreting and comparing findings across different studies.

### Time preferences and attitudes towards vaccinations

Table 4 presents the results from a multinomial logistic regression on a combined dataset, accounting for hyperbolic and constant discounting in both RP and SP data. Additional regression results at the country level can be found in the Supplementary Material.

We notice similar patterns across RP and SP data. Hesitant respondents are more impatient and impulsive compared to accepters, and respondents who refuse vaccination are more patient and less impulsive compared to accepters, even though the latter result is statistically significant only for stated vaccination status. The results for the SP data are robust to variations in the threshold used to define the three groups (see Table A4 in the Supplementary Material). We used the constant discounting model as our main specification, focusing on impatience (δ) as the key time preference parameter. This choice was motivated by the observation that the modal and median response across countries was β = 1, and Wilcoxon signed-rank tests generally failed to reject the hypothesis that β equals 1. To assess robustness, we additionally estimated models based on quasi-hyperbolic discounting, accounting for both impatience (δ) and present/future bias (β); these results are reported in the Supplementary Material (see Tables A21-A28). They indicate that there were only minor differences compared to the constant discounting model, with very few countries where β became statistically significant.

**Table 4:** Multinomial logistic regression on vaccination status

		Revealed Vaccination Status				Stated Vaccination Status			
		Hyperbolic		Constant		Hyperbolic		Constant	
		(1)	(2)	(3)	(4)	(5)	(6)	(7)	(8)
Refuser	rho2	-0.06	-0.001	-	-	-0.32**	-0.25***	-	-
		(0.04)	(0.04)			(0.127)	(0.03)		
	Beta	< 0.01	< 0.01	-	-	0.01*	0.01***	-	-
		(0.01)	(< 0.01)			(< 0.01)	(< 0.01)		
	Average rho	-	-	-0.02	0.04	-	-	-0.32***	− 0.25***
				(0.11)	(0.04)			(0.13)	(0.03)
	Cons	-2.26***	-2.81***	-2.27***	-2.82***	-1.19***	-1.87***	-1.17***	-1.85***
		(0.021)	(0.02)	(0.21)	(0.08)	(0.17)	(0.06)	(0.17)	(0.06)
Hesitant	rho2	0.20	0.27***	-	-	0.103	0.15***	-	-
		(0.13)	(0.05)			(0.07)	(0.03)		
	Beta	-0.01	-0.01*	-	-	-0.01***	-0.01**	-	-
		(0.01)	(0.01)			(< 0.01)	(< 0.01)		
	Average rho	-	-	0.18	0.26***	-	-	0.10	0.15***
				(0.14)	(0.05)			(0.07)	(0.03)
	Cons	-2.94***	-3.77***	-2.95***	-3.78***	-1.12***	-1.4***	-1.14***	-1.44***
		(0.31)	(0.12)	(0.32)	(0.12)	(0.02)	(0.05)	(0.12)	(0.05)
Vaccinated	(Baseline)	-	-	-	-	-	-	-	-
	Country FE	No	Yes	No	Yes	No	Yes	No	Yes
	Observations	49,227	49,227	49,227	49,227	49,227	49,227	49,227	49,227
	Pseudo R2	< 0.01	0.11	< 0.01	0.11	< 0.01	0.07	< 0.01	0.07
	Log-Likelihood	-24016.35	-21416.33	-24018.8	-21417.66	-45403.879	-42330.819	-45402.35	-42329.48
	Akaike’s Crit	48044.70	42928.67	48045.6	42927.31	90819.758	84757.638	90812.69	84750.96
	Bayesian Crit	48097.53	43351.27	48080.81	43332.3	90872.583	85180.24	90847.91	85155.95

Note: *** *p* < 0.01, ** *p* < 0.05, * *p* < 0.1. Standard errors are in parentheses. In column (1), (3), (5) and (7) standard errors are clustered at the country level

### Time preferences, risk, and sociodemographic characteristics

**Fig. 2:**
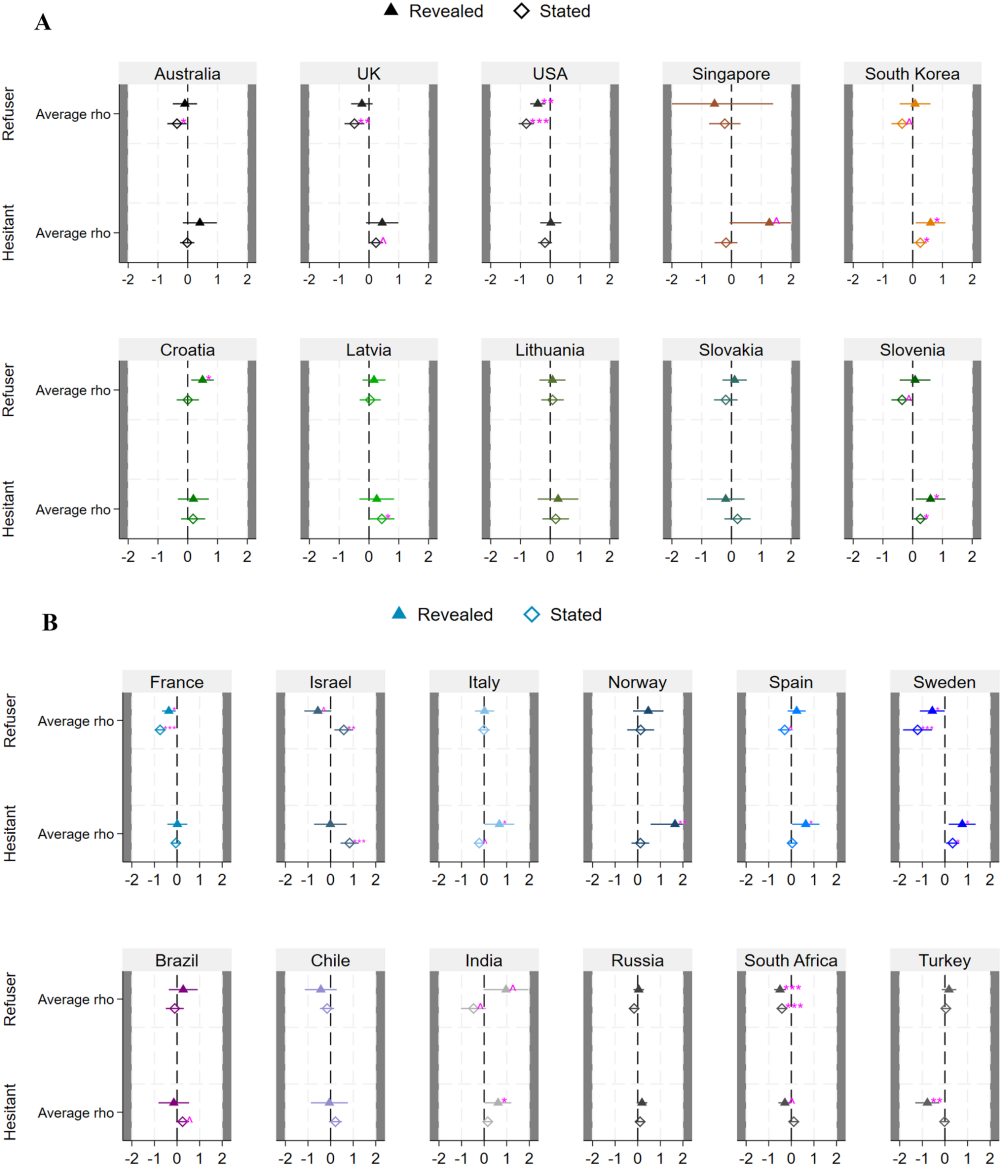
Panel A - Results from the multinomial logistic regression models on vaccination status (revealed and stated) Notes: Full regression output is reported in Tables A13, A14, A17, and A18 of the Supplementary Material. ^*p* < 0.1; *p < 0.5; **p < 0.01; ***p < 0.001. Panel B - Results from the multinomial logistic regression models on vaccination status (revealed and stated) Notes: Full regression output is reported in Table A15, A16, A19, A20 of the Supplementary Material. ^*p* < 0.1; *p < 0.5; **p < 0.01; ***p < 0.001

In this section, we present the findings derived from our regression models. Figure 2A and B depict the coefficients pertaining to various countries categorized by groups (see Supplementary Material for full regression results). Our analysis reveals minimal differences between RP and SP data, consistent with prior regression analyses. We find that individuals identified as refusers tend to exhibit lower discount rates compared to their vaccinated counterparts. However, this pattern is reversed for the hesitant group.

Significant statistical differences in discount rates were noted in the United States, France, Sweden, and South Africa, with refusers consistently demonstrating lower discount rates than vaccinated individuals in both revealed and stated preferences (*p* < 0.05). In Australia and the United Kingdom, significant differences were observed in stated vaccination preferences (*p* < 0.05). Conversely, in Croatia and Israel, refusers had higher discount rates (*p* < 0.05), with Israel showing conflicting results between revealed (positive coefficient, *p* < 0.1) and stated preferences (negative coefficient, *p* < 0.05), potentially attributed to a low number of refusers according to their revealed vaccination status.

Regarding hesitant respondents, statistically significant and positive coefficients for stated preferences are evident in Latvia and Israel (*p* < 0.05), while positive coefficients are identified in Italy, Norway, and Spain for revealed vaccination status (*p* < 0.05). Positive and statistically significant discount rate coefficients were observed for both vaccination statuses in Slovenia, Sweden, and South Korea (*p* < 0.05). Turkey was unique, with hesitant respondents showing negative coefficients for revealed vaccination status.

We also explore the impact of willingness to take risks concerning one’s health on the likelihood of being a refuser or hesitant. Overall, this factor exhibits a marginal but negative influence on the probability of being classified as either a vaccine refuser or hesitant. Notably, statistically significant results were found for vaccine refuser respondents in India, Russia, Singapore, Slovenia, and Turkey (*p* < 0.05). Turkish respondents, along with their Slovenian and South Korean counterparts, report similar results regarding the probability of being vaccine hesitant. Latvia showed an increased willingness to take health risks among the hesitant group (*p* < 0.05).

Regarding risk tolerance, our findings diverge from previous research, possible due to different risk scales and the timing of the surveys. Notably, the results differ from those reported by Massin et al. [61, 62], who identified a positive correlation between risk aversion and the influenza vaccination rates of general practitioners in France, as well as their inclination to recommend such vaccinations to patients. Similarly, Lepinteur et al. [63] observed a decline in vaccine hesitancy corresponding to increased risk aversion across a survey conducted in France, Germany, Italy, Spain, and Sweden. One potential explanation for these contrasting outcomes could be attributed to the use of a general risk scale in the study by Lepinteur et al. [63], whereas our study employed a health risk scale. Additionally, the disparities may arise from the timing of the surveys, with Lepinteur et al.‘s research conducted earlier than our own, specifically in June 2021. Furthermore, in a study employing a similar general risk scale, Okubo et al. [44] reported no significant impact of risk aversion on vaccination behaviors in Japan.

Furthermore, we explored how sociodemographic characteristics affect vaccination decisions. In Anglo-Saxon and Eastern European countries, women were more likely to refuse vaccines than men, the evidence being mixed for other groups of countries. Older individuals generally displayed a higher probability of being vaccinated. Individuals with higher education and income levels tend to be more inclined to be vaccinated across all countries. Sweden stands out as the only country reporting a positive probability of being a refuser at the 95% confidence level for revealed vaccination status.

## Discussion

This study sought to empirically investigate the effect of time preferences on vaccination attitudes and behaviors, utilizing choice list methodology in 22 countries. In this context, we disentangled and tested the role of present bias and the discount rate using a quasi-hyperbolic discounting function. Our findings revealed no significant differences in present or future bias among hesitant, refusing, and vaccinated respondents based on RP data on vaccination decisions. This confirms previous findings from Guillon et al. [9]. However, in hypothetical scenarios hesitant respondents showed lower present bias, while refusers exhibited a higher present bias compared to the vaccinated respondents.

On the contrary, significant differences in the discount rates were observed among these groups, both under constant and hyperbolic discounting assumptions. Hesitant respondents revealed larger discount rates compared to the vaccinated respondents aligning with the findings of Hudson et al. [38] and Okamoto et al. [40], indicating a stronger preference for immediate outcomes, while refusers showed lower discount rates, indicating stronger preferences for future outcomes. These patterns persisted even after accounting for risk preferences and other sociodemographic characteristics, though with reduced significance across countries. The observed differences in discounting between vaccine refusers and hesitant individuals may reflect different motivational and cognitive patterns. Vaccine refusers may display a lower discount rate due to increased focus on potential negative future consequences of vaccination. Conversely, vaccine-hesitant individuals exhibit higher discount rates, which may reflect that relatively more weight is given to the immediate costs and less to the reduced infection risk in the future. These findings highlight the importance of distinguishing between hesitant and refusing individuals, as the underlying psychological mechanisms differ and require tailored public health interventions. This distinction, often overlooked in empirical studies, is crucial for designing effective vaccination campaigns and public health messages [27]. Future research should further explore these differences by investigating the interplay between time preferences, perceived risk, and trust in public health institutions. To this end, using alternative methods, such as qualitative interviews or longitudinal studies, could provide better insights into the motivations underlying these patterns.

The existing literature generally suggests that individuals with higher discount rates are more inclined to delay vaccinations or exhibit vaccine hesitancy [13, 35, 36, 38, 40], except for Okubo et al. [44], who reported non-significant results. These findings align with our results regarding the vaccine-hesitant population but contrast with those pertaining to vaccine refusers. This discrepancy may be attributed to several factors, including different measurements of time preferences, which may capture distinct cognitive or behavioral aspects of time discounting, differences in the timing of data collection, and the lack of a clear distinction between hesitant and refusing individuals. For instance, if a disproportionately large number of hesitant individuals participated in these studies, the results could be skewed. By contrast, our findings align with those of Guillon et al. [9] who found a positive association between respondents’ discounting and their COVID-19 vaccination status. This reinforces our findings as they implemented a methodological approach more closely aligned with our analysis. While our study relies on a specific approach, the systematic exploration of how various methods influence observed relationships between time preferences and vaccination decisions is currently underdeveloped in the literature. Future research could address this gap by systematically comparing measurement approaches and their implications, thereby enhancing our understanding of these complex interactions.

As an additional control, we conducted fixed-effect logistic regressions on the binary dependent variable vaccinated/not vaccinated for both RP and SP, following the approach of Halilova et al. [36]. In the case of revealed vaccination status, we found that higher discount rates were associated with non-vaccination decisions, but the coefficient did not attain statistical significance (b = 0.03, 95% C.I: -0.03; 0.09, *p* = 0.40). Interestingly, when transitioning to the hypothetical scenario, we observed the opposite outcome: lower levels of discounting were linked to a higher likelihood of delaying or refusing vaccination (b= -0.05, 95% C.I: -0.10; -0.01, *p* = 0.02). Although this difference is challenging to reconcile with the prior literature, it could be attributed to the proportions of hesitant respondents (21%) and refuser respondents (18%). When we ran the same regression while excluding the refuser segment, we obtained significant results consistent with the literature (b = 0.08, 95% C.I.: 0.02; 0.14, *p* < 0.01). Conversely, when we excluded the hesitant segment, we found a statistically significant negative relationship between discounting and non-vaccination behaviors (b= -0.26, 95% C.I.: -0.32; -0.19, *p* < 0.001). This substantial difference lends support to the needs of differentiating hesitant individuals from outright refusers and the possibility of a larger proportion of hesitant respondents completing the questionnaire in the studies mentioned earlier [64–66].

From a policy perspective, our findings suggest that understanding time preferences can help tailor vaccination policies/strategies. Compared to refusers, hesitant respondents may be more likely to get the vaccine if their specific concerns about the vaccination (i.e., vaccine features, cost, mode of administration and contextual policies) are addressed. Therefore, to promote vaccine acceptance, sustainable policy strategies can employ techniques or targeted communication that emphasize the future consequences more prominently [36, 64]. These techniques are well established in the literature given that large discount rates have been found to be associated with a set of other health-related issues, particularly addictive behavior [65–68] and obesity [69, 70]. Communication campaigns could also focus more on the short-term benefits of vaccination, for instance by stressing the improved access it may bring to social events, the reduced chances of getting sick and the increased safety for significant others.

While our estimate of present bias (i.e., β) did not significantly differ from 1, we could not reject the constant discounting model. This result aligns with some previous studies, especially with Guillon at al. [9], but contrasts with others that have identified significant present bias effects in intertemporal health decisions, highlighting the heterogeneity in this area [42, 71–73]. In addition, our finding that a large proportion of respondents exhibited future-biased preferences (β > 1) is consistent with observations from recent empirical studies using similar elicitation methods [9, 74–76]. This recurring pattern suggests that future bias may be a common feature in stated time preference data, particularly when hypothetical choice lists with relatively small monetary stakes are used. One possible explanation is that, in such contexts, individuals may overweight delayed rewards relative to immediate ones, possibly reflecting a desire to appear patient or due to reduced salience of immediate benefits in hypothetical tasks. Alternatively, part of the observed future bias could stem from noise or inconsistencies in individual responses, a phenomenon well-documented in the broader literature on time preference elicitation [2]. Although this limits the interpretation of absolute β estimates, the relative comparisons across vaccination groups in our study remain informative for understanding behavioral patterns.

Several limitations of the study should be taken into consideration when interpreting the results. Firstly, it is important to note that the time preference task was not incentivized, which may have influenced respondent engagement, accuracy and reliability of reported time preferences. The absence of real monetary stakes could reduce motivation, potentially leading to response biases or lower data quality. However, empirical evidence suggests that hypothetical incentives and real incentives generate similar time preferences [77–80]. Given this empirical support, we believe that our elicited beta and delta parameters remain valid for analyzing intertemporal vaccination decisions, though we acknowledge that future studies should further investigate potential biases introduced by hypothetical tasks. Furthermore, the measure of present bias employed in this study, while providing valuable insights, may be considered relatively crude, potentially lacking the precision required to capture the full complexity of present-biased preferences accurately. In particular, choice list methodology may be affected by several distortions, such as a tendency for switching in the middle, the confounding influence of interest rates, and the bias caused by utility curvature [24, 81–84]. As a result, the estimation of present bias and its implications for vaccination behavior should be interpreted with caution. Our assumption of linear utility simplifies the estimation process, but it does not account for potential influence of risk aversion, which could influence individuals’ intertemporal preferences. Incorporating a CRRA utility function would likely result in lower estimated discount rates, as risk-averse individuals tend to discount future outcomes more steeply [42, 81]. However, given the relatively small monetary stakes involved in our survey, we anticipate that the impact of utility curvature on our findings is minimal, and previous literature suggests that utility curvature effects are less pronounced in such contexts [85, 86]. As such, we do not anticipate it materially affecting the robustness of our results. Future research could explore the implications of different utility functions on intertemporal preferences and vaccination behavior to further validate the robustness of our results. Furthermore, the use of an online survey for data collection introduces concerns about data quality and may limit the representativeness of the sample due to selection bias. Online surveys, although convenient and accessible, may not fully capture the diversity of the broader population, e.g. missing people that lack internet access and migrants, and are susceptible to response biases and inaccuracies, which can impact the generalizability of the findings. Lastly, our study offers insights into vaccination and time preferences from upper-middle/high-income countries. Consequently, the lessons and findings derived from this dataset may not be directly applicable to low- or middle-income countries. Future research endeavors should aim to address these limitations by incorporating more refined measurement tools, implementing diverse data collection strategies, and employing rigorous sampling techniques to enhance the comprehensiveness and validity of the study’s outcomes. Future studies might also consider applying simpler elicitation tasks, especially when using online studies. For instance, Rafai et al. [87] found that stated preferences on willingness to take risks and patience as measured by 0–10 Likert scales [88] outperformed empirically elicited preferences such as the convex time budget task [42] in predicting reported compliance with COVID-19 guidelines, whilst Guillon et al. [9] used both an incentivized choice task and a hypothetical scaling question and found the association between discounting and vaccination to be robust to this measure. Future research could also replicate our analysis controlling for measures of risk perceptions related to COVID-19 (or infectious diseases in general), as these have been found to be an important determinant of vaccination decisions [89–92].

## Conclusion

Overall, this study contributes to the existing literature by providing detailed insights into the influence of time preferences, particularly present bias and discount rates, on vaccination behaviors. By examining these dynamics across diverse countries and populations, the study not only enhances our understanding of the underlying mechanisms influencing vaccination decisions but also offers valuable implications for the design and implementation of tailored interventions and policies to improve vaccination uptake and public health outcomes.

## Electronic supplementary material

Below is the link to the electronic supplementary material.

Supplementary Material 1

Supplementary Material 2

## Footnotes

1 See Sect. Related literature of Supplementary Material for a demonstration of this procedure.
