# Supplementary material for: Time preferences and COVID-19 vaccination uptake
Source: Eur J Health Econ. 2025 Jun 14;27(1):47–63. doi: 10.1007/s10198-025-01801-7 (PMC12929247; doi:10.1007/s10198-025-01801-7)
Supplement: Supplementary file 1 — Supplementary Material 1 [file 10198_2025_1801_MOESM1_ESM.docx]

**Supplementary Materials B**

The following tables report the Wilcoxon signed-rank tests conducted at the country level to determine whether the distribution of impulsivity differed from 1 across vaccination status, and the t-tests of means performed at the country level to assess whether impulsivity and impatience differed by vaccination status.

**Table B1** Wilcoxon signed-rank test for impulsivity across countries and vaccination groups

|  | Overall | | Refuser | | Hesitant | | Accepters | |
| --- | --- | --- | --- | --- | --- | --- | --- | --- |
| Country | Z-score (beta=1) | P-value | Z-score (beta=1) | P-value | Z-score (beta=1) | P-value | Z-score (beta=1) | P-value |
| Australia | -4.50 | 0.00 | -2.46 | 0.01 | 0.18 | 0.86 | -3.75 | 0.00 |
| Brazil | 11.08 | 0.00 | -0.74 | 0.47 | 2.19 | 0.03 | 11.12 | 0.00 |
| Chile | 14.06 | 0.00 | -0.34 | 0.73 | 0.99 | 0.33 | 14.14 | 0.00 |
| Croatia | 2.28 | 0.02 | 1.75 | 0.08 | 0.09 | 0.93 | 1.85 | 0.06 |
| France | 1.56 | 0.12 | 0.92 | 0.36 | -0.71 | 0.48 | 1.63 | 0.10 |
| India | -0.73 | 0.47 | 1.08 | 0.29 | -0.30 | 0.77 | -1.17 | 0.24 |
| Israel | 0.49 | 0.63 | -1.26 | 0.21 | -0.62 | 0.54 | 0.84 | 0.40 |
| Italy | 6.35 | 0.00 | 2.71 | 0.01 | 0.75 | 0.46 | 5.90 | 0.00 |
| Latvia | 4.55 | 0.00 | 2.35 | 0.02 | 0.39 | 0.70 | 3.95 | 0.00 |
| Lithuania | 4.02 | 0.00 | 1.67 | 0.10 | 0.90 | 0.37 | 3.41 | 0.00 |
| Norway | -1.58 | 0.11 | -0.56 | 0.58 | 0.68 | 0.52 | -1.49 | 0.14 |
| Russia | 1.99 | 0.05 | -0.49 | 0.63 | 2.44 | 0.01 | 0.68 | 0.50 |
| Singapore | 1.27 | 0.20 | -1.99 | 0.13 | -0.42 | 0.72 | 1.66 | 0.10 |
| Slovakia | 2.95 | 0.00 | 1.15 | 0.25 | 3.36 | 0.00 | 1.78 | 0.08 |
| Slovenia | 2.95 | 0.01 | 0.82 | 0.41 | 1.07 | 0.29 | 2.57 | 0.01 |
| South Africa | 4.85 | 0.00 | 1.69 | 0.09 | 1.41 | 0.16 | 4.06 | 0.00 |
| South Korea | -7.98 | 0.00 | 0.06 | 0.95 | -0.97 | 0.33 | -8.31 | 0.00 |
| Spain | 10.33 | 0.00 | 3.52 | 0.00 | 1.79 | 0.07 | 9.60 | 0.00 |
| Sweden | -1.53 | 0.13 | -0.37 | 0.71 | 0.05 | 0.96 | -1.69 | 0.09 |
| Turkey | 4.77 | 0.00 | -1.98 | 0.05 | 2.03 | 0.04 | 5.13 | 0.00 |
| UK | -5.07 | 0.00 | -1.38 | 0.17 | -0.07 | 0.95 | -4.94 | 0.00 |
| USA | -2.46 | 0.01 | -0.99 | 0.32 | -1.96 | 0.05 | -1.75 | 0.08 |

**Table B2** t-tests of means for impulsivity (beta) with unequal variance between vaccination groups within each country (Refuser vs hesitant)

| **Refuser vs hesitant** | N. Refuser | N. Hesitant | Mean Refuser | Mean hesitant | Diff. | St. error | t-value | p-value |
| --- | --- | --- | --- | --- | --- | --- | --- | --- |
| Australia | 164 | 69 | 1.30 | 2.11 | -0.81 | 0.46 | -1.75 | 0.08 |
| Brazil | 52 | 49 | 1.62 | 4.06 | -2.43 | 1.38 | -1.75 | 0.08 |
| Chile | 65 | 43 | 2.20 | 1.92 | 0.28 | 1.00 | 0.30 | 0.78 |
| Croatia | 219 | 107 | 3.35 | 2.37 | 0.98 | 0.81 | 1.20 | 0.23 |
| France | 293 | 115 | 2.14 | 1.95 | 0.18 | 0.56 | 0.35 | 0.74 |
| India | 22 | 61 | 3.15 | 1.11 | 2.05 | 1.28 | 1.60 | 0.12 |
| Israel | 85 | 45 | 1.32 | 1.27 | 0.05 | 0.44 | 0.10 | 0.90 |
| Italy | 139 | 42 | 2.13 | 2.92 | -0.79 | 1.14 | -0.70 | 0.49 |
| Latvia | 200 | 72 | 2.40 | 2.15 | 0.25 | 0.67 | 0.35 | 0.71 |
| Lithuania | 167 | 55 | 1.97 | 1.60 | 0.36 | 0.44 | 0.85 | 0.41 |
| Norway | 52 | 16 | 1.34 | 4.19 | -2.85 | 2.40 | -1.20 | 0.25 |
| Russia | 659 | 611 | 1.63 | 1.78 | -0.14 | 0.23 | -0.60 | 0.54 |
| Singapore | 12 | 11 | 0.94 | 1.17 | -0.23 | 0.34 | -0.65 | 0.52 |
| Slovakia | 206 | 79 | 2.45 | 2.13 | 0.31 | 0.80 | 0.40 | 0.70 |
| Slovenia | 231 | 91 | 1.63 | 2.29 | -0.66 | 0.63 | -1.05 | 0.30 |
| South Africa | 453 | 335 | 2.17 | 1.98 | 0.19 | 0.37 | 0.50 | 0.61 |
| South Korea | 115 | 104 | 2.10 | 1.60 | 0.51 | 0.56 | 0.90 | 0.36 |
| Spain | 150 | 52 | 3.10 | 3.72 | -0.62 | 1.41 | -0.45 | 0.66 |
| Sweden | 115 | 64 | 1.20 | 2.40 | -1.20 | 0.86 | -1.40 | 0.17 |
| Turkey | 257 | 91 | 1.34 | 2.65 | -1.31 | 0.75 | -1.75 | 0.08 |
| UK | 229 | 82 | 1.98 | 1.20 | 0.78 | 0.35 | 2.20 | 0.03 |
| US | 464 | 192 | 2.55 | 1.36 | 1.19 | 0.38 | 3.15 | 0.00 |

**Table B3** t-tests of means for impulsivity (beta) with unequal variance between vaccination groups within each country (Refuser vs accepters)

| **Refusers vs accepters** | N. Refuser | N. Hesitant | Mean Refuser | Mean hesitant | Diff. | St. error | t-value | p-value |
| --- | --- | --- | --- | --- | --- | --- | --- | --- |
| Australia | 164 | 2720 | 1.30 | 1.64 | -0.34 | 0.26 | -1.30 | 0.19 |
| Brazil | 52 | 2882 | 1.62 | 2.47 | -0.85 | 0.59 | -1.45 | 0.16 |
| Chile | 65 | 2876 | 2.20 | 3.17 | -0.97 | 0.84 | -1.15 | 0.25 |
| Croatia | 219 | 710 | 3.35 | 1.84 | 1.51 | 0.57 | 2.65 | 0.01 |
| France | 293 | 2708 | 2.14 | 1.90 | 0.24 | 0.36 | 0.65 | 0.51 |
| India | 22 | 3014 | 3.15 | 2.06 | 1.10 | 1.27 | 0.85 | 0.40 |
| Israel | 85 | 1354 | 1.32 | 1.79 | -0.47 | 0.42 | -1.10 | 0.27 |
| Italy | 139 | 2811 | 2.13 | 1.87 | 0.26 | 0.47 | 0.55 | 0.59 |
| Latvia | 200 | 795 | 2.40 | 2.59 | -0.19 | 0.46 | -0.40 | 0.68 |
| Lithuania | 167 | 764 | 1.97 | 2.40 | -0.43 | 0.43 | -1.00 | 0.31 |
| Norway | 52 | 945 | 1.34 | 1.60 | -0.26 | 0.29 | -0.90 | 0.37 |
| Russia | 659 | 1581 | 1.63 | 1.69 | -0.05 | 0.18 | -0.30 | 0.76 |
| Singapore | 12 | 970 | 0.94 | 1.81 | -0.86 | 0.14 | -6.05 | 0.00 |
| Slovakia | 206 | 681 | 2.45 | 2.07 | 0.37 | 0.47 | 0.80 | 0.43 |
| Slovenia | 231 | 720 | 1.63 | 1.98 | -0.35 | 0.30 | -1.15 | 0.24 |
| South Africa | 453 | 2084 | 2.17 | 2.42 | -0.26 | 0.29 | -0.85 | 0.38 |
| South Korea | 115 | 2728 | 2.10 | 1.41 | 0.70 | 0.46 | 1.55 | 0.13 |
| Spain | 150 | 3038 | 3.10 | 1.95 | 1.15 | 0.66 | 1.75 | 0.09 |
| Sweden | 115 | 1302 | 1.20 | 1.56 | -0.36 | 0.18 | -2.00 | 0.04 |
| Turkey | 257 | 2714 | 1.34 | 2.39 | -1.05 | 0.21 | -5.10 | 0.00 |
| UK | 229 | 2747 | 1.98 | 1.76 | 0.22 | 0.35 | 0.65 | 0.53 |
| US | 464 | 2348 | 2.55 | 1.85 | 0.71 | 0.33 | 2.15 | 0.03 |

**Table B4** t-tests of means for impulsivity (beta) with unequal variance between vaccination groups within each country (Hesitant vs accepters)

| **Hesitant vs accepters** | N. Hesitant | N. Accepters | Mean hesitant | Mean Accepters | Diff. | St. error | t-value | p-value |
| --- | --- | --- | --- | --- | --- | --- | --- | --- |
| Australia | 69 | 2720 | 2.11 | 1.64 | 0.47 | 0.40 | 1.20 | 0.24 |
| Brazil | 49 | 2882 | 4.06 | 2.47 | 1.58 | 1.25 | 1.25 | 0.21 |
| Chile | 43 | 2876 | 1.92 | 3.17 | -1.25 | 0.58 | -2.15 | 0.03 |
| Croatia | 107 | 710 | 2.37 | 1.84 | 0.54 | 0.63 | 0.85 | 0.39 |
| France | 115 | 2708 | 1.95 | 1.90 | 0.05 | 0.45 | 0.10 | 0.90 |
| India | 61 | 3014 | 1.11 | 2.06 | -0.95 | 0.14 | -6.75 | 0.00 |
| Israel | 45 | 1354 | 1.27 | 1.79 | -0.52 | 0.21 | -2.45 | 0.02 |
| Italy | 42 | 2811 | 2.92 | 1.87 | 1.05 | 1.05 | 1.00 | 0.32 |
| Latvia | 72 | 795 | 2.15 | 2.59 | -0.44 | 0.59 | -0.75 | 0.46 |
| Lithuania | 55 | 764 | 1.60 | 2.40 | -0.80 | 0.33 | -2.40 | 0.02 |
| Norway | 16 | 945 | 4.19 | 1.60 | 2.58 | 2.39 | 1.10 | 0.30 |
| Russia | 611 | 1581 | 1.78 | 1.69 | 0.09 | 0.21 | 0.40 | 0.67 |
| Singapore | 11 | 970 | 1.17 | 1.81 | -0.64 | 0.37 | -1.70 | 0.11 |
| Slovakia | 79 | 681 | 2.13 | 2.07 | 0.06 | 0.71 | 0.10 | 0.93 |
| Slovenia | 91 | 720 | 2.29 | 1.98 | 0.31 | 0.62 | 0.50 | 0.62 |
| South Africa | 335 | 2084 | 1.98 | 2.42 | -0.44 | 0.29 | -1.50 | 0.13 |
| South Korea | 104 | 2728 | 1.60 | 1.41 | 0.19 | 0.34 | 0.55 | 0.58 |
| Spain | 52 | 3038 | 3.72 | 1.95 | 1.78 | 1.25 | 1.40 | 0.16 |
| Sweden | 64 | 1302 | 2.40 | 1.56 | 0.84 | 0.85 | 1.00 | 0.33 |
| Turkey | 91 | 2714 | 2.65 | 2.39 | 0.26 | 0.74 | 0.35 | 0.72 |
| UK | 82 | 2747 | 1.20 | 1.76 | -0.56 | 0.14 | -4.00 | 0.00 |
| US | 192 | 2348 | 1.36 | 1.85 | -0.48 | 0.24 | -2.05 | 0.04 |

**Table B5** t-tests of means for impatience (rho2) with unequal variance between vaccination groups within each country (Refuser vs hesitant)

| **Refusers vs hesitant** | N. Hesitant | N. Accepters | Mean hesitant | Mean Accepters | Diff. | St. error | t-value | p-value |
| --- | --- | --- | --- | --- | --- | --- | --- | --- |
| Australia | 164 | 69 | 0.32 | 0.52 | -0.20 | 0.07 | -2.85 | 0.01 |
| Brazil | 52 | 49 | 0.40 | 0.44 | -0.04 | 0.10 | -0.45 | 0.64 |
| Chile | 65 | 43 | 0.23 | 0.28 | -0.05 | 0.08 | -0.60 | 0.56 |
| Croatia | 219 | 107 | 0.43 | 0.37 | 0.06 | 0.05 | 1.20 | 0.24 |
| France | 293 | 115 | 0.33 | 0.38 | -0.05 | 0.05 | -0.95 | 0.34 |
| India | 22 | 61 | 0.83 | 0.76 | 0.07 | 0.13 | 0.60 | 0.57 |
| Israel | 85 | 45 | 0.28 | 0.44 | -0.16 | 0.08 | -1.95 | 0.05 |
| Italy | 139 | 42 | 0.35 | 0.50 | -0.15 | 0.09 | -1.75 | 0.08 |
| Latvia | 200 | 72 | 0.42 | 0.41 | 0.01 | 0.06 | 0.25 | 0.82 |
| Lithuania | 167 | 55 | 0.35 | 0.38 | -0.03 | 0.07 | -0.45 | 0.67 |
| Norway | 52 | 16 | 0.36 | 0.68 | -0.32 | 0.14 | -2.35 | 0.03 |
| Russia | 659 | 611 | 0.32 | 0.36 | -0.04 | 0.02 | -1.65 | 0.10 |
| Singapore | 12 | 11 | 0.18 | 0.65 | -0.48 | 0.18 | -2.65 | 0.02 |
| Slovakia | 206 | 79 | 0.35 | 0.34 | 0.01 | 0.06 | 0.15 | 0.87 |
| Slovenia | 231 | 91 | 0.35 | 0.35 | 0.00 | 0.06 | 0.05 | 0.96 |
| South Africa | 453 | 335 | 0.35 | 0.39 | -0.04 | 0.03 | -1.15 | 0.25 |
| South Korea | 115 | 104 | 0.35 | 0.43 | -0.08 | 0.06 | -1.50 | 0.13 |
| Spain | 150 | 52 | 0.35 | 0.41 | -0.06 | 0.08 | -0.75 | 0.46 |
| Sweden | 115 | 64 | 0.24 | 0.50 | -0.25 | 0.07 | -3.70 | 0.00 |
| Turkey | 257 | 91 | 0.67 | 0.48 | 0.19 | 0.06 | 3.40 | 0.00 |
| UK | 229 | 82 | 0.34 | 0.49 | -0.15 | 0.06 | -2.45 | 0.02 |
| US | 464 | 192 | 0.44 | 0.54 | -0.10 | 0.04 | -2.45 | 0.02 |

**Table B6** t-tests of means for impatience (rho2) with unequal variance between vaccination groups within each country (Refuser vs accepters)

| **Refusers vs accepters** | N. Refuser | N. Hesitant | Mean Refuser | Mean hesitant | Diff. | St. error | t-value | p-value |
| --- | --- | --- | --- | --- | --- | --- | --- | --- |
| Australia | 164 | 2720 | 0.32 | 0.37 | -0.05 | 0.03 | -1.45 | 0.14 |
| Brazil | 52 | 2882 | 0.40 | 0.41 | -0.01 | 0.07 | -0.10 | 0.91 |
| Chile | 65 | 2876 | 0.23 | 0.30 | -0.07 | 0.05 | -1.50 | 0.14 |
| Croatia | 219 | 710 | 0.43 | 0.30 | 0.14 | 0.04 | 3.70 | 0.00 |
| France | 293 | 2708 | 0.33 | 0.35 | -0.02 | 0.03 | -0.80 | 0.43 |
| India | 22 | 3014 | 0.83 | 0.61 | 0.22 | 0.11 | 1.95 | 0.06 |
| Israel | 85 | 1354 | 0.28 | 0.38 | -0.11 | 0.04 | -2.45 | 0.02 |
| Italy | 139 | 2811 | 0.35 | 0.32 | 0.03 | 0.04 | 0.75 | 0.45 |
| Latvia | 200 | 795 | 0.42 | 0.37 | 0.05 | 0.04 | 1.35 | 0.18 |
| Lithuania | 167 | 764 | 0.35 | 0.32 | 0.03 | 0.04 | 0.70 | 0.50 |
| Norway | 52 | 945 | 0.36 | 0.26 | 0.10 | 0.06 | 1.55 | 0.12 |
| Russia | 659 | 1581 | 0.32 | 0.33 | 0.00 | 0.02 | -0.15 | 0.87 |
| Singapore | 12 | 970 | 0.18 | 0.39 | -0.22 | 0.10 | -2.15 | 0.05 |
| Slovakia | 206 | 681 | 0.35 | 0.32 | 0.03 | 0.03 | 0.90 | 0.36 |
| Slovenia | 231 | 720 | 0.35 | 0.30 | 0.06 | 0.03 | 1.65 | 0.10 |
| South Africa | 453 | 2084 | 0.35 | 0.43 | -0.08 | 0.02 | -3.50 | 0.00 |
| South Korea | 115 | 2728 | 0.35 | 0.32 | 0.03 | 0.04 | 0.70 | 0.49 |
| Spain | 150 | 3038 | 0.35 | 0.29 | 0.07 | 0.04 | 1.75 | 0.08 |
| Sweden | 115 | 1302 | 0.24 | 0.29 | -0.04 | 0.03 | -1.30 | 0.20 |
| Turkey | 257 | 2714 | 0.67 | 0.61 | 0.06 | 0.03 | 2.00 | 0.04 |
| UK | 229 | 2747 | 0.34 | 0.33 | 0.01 | 0.03 | 0.30 | 0.78 |
| US | 464 | 2348 | 0.44 | 0.50 | -0.06 | 0.02 | -2.45 | 0.01 |

**Table B7** t-tests of means for impatience (rho2) with unequal variance between vaccination groups within each country (Hesitant vs accepters)

| **Hesitant vs accepters** | N. Hesitant | N. Accepters | Mean hesitant | Mean Accepters | Diff. | St. error | t-value | p-value |
| --- | --- | --- | --- | --- | --- | --- | --- | --- |
| Australia | 69 | 2720 | 0.52 | 0.37 | 0.14 | 0.06 | 2.40 | 0.02 |
| Brazil | 49 | 2882 | 0.44 | 0.41 | 0.04 | 0.07 | 0.55 | 0.60 |
| Chile | 43 | 2876 | 0.28 | 0.30 | -0.03 | 0.07 | -0.35 | 0.72 |
| Croatia | 107 | 710 | 0.37 | 0.30 | 0.07 | 0.05 | 1.60 | 0.12 |
| France | 115 | 2708 | 0.38 | 0.35 | 0.03 | 0.04 | 0.60 | 0.54 |
| India | 61 | 3014 | 0.76 | 0.61 | 0.15 | 0.06 | 2.45 | 0.02 |
| Israel | 45 | 1354 | 0.44 | 0.38 | 0.06 | 0.07 | 0.80 | 0.44 |
| Italy | 42 | 2811 | 0.50 | 0.32 | 0.18 | 0.08 | 2.35 | 0.02 |
| Latvia | 72 | 795 | 0.41 | 0.37 | 0.03 | 0.06 | 0.60 | 0.55 |
| Lithuania | 55 | 764 | 0.38 | 0.32 | 0.05 | 0.06 | 0.90 | 0.37 |
| Norway | 16 | 945 | 0.68 | 0.26 | 0.42 | 0.12 | 3.50 | 0.00 |
| Russia | 611 | 1581 | 0.36 | 0.33 | 0.04 | 0.02 | 1.80 | 0.08 |
| Singapore | 11 | 970 | 0.65 | 0.39 | 0.26 | 0.15 | 1.70 | 0.12 |
| Slovakia | 79 | 681 | 0.34 | 0.32 | 0.02 | 0.05 | 0.45 | 0.67 |
| Slovenia | 91 | 720 | 0.35 | 0.30 | 0.05 | 0.05 | 1.05 | 0.29 |
| South Africa | 335 | 2084 | 0.39 | 0.43 | -0.04 | 0.03 | -1.65 | 0.10 |
| South Korea | 104 | 2728 | 0.43 | 0.32 | 0.11 | 0.04 | 2.65 | 0.01 |
| Spain | 52 | 3038 | 0.41 | 0.29 | 0.12 | 0.07 | 1.85 | 0.07 |
| Sweden | 64 | 1302 | 0.50 | 0.29 | 0.21 | 0.06 | 3.45 | 0.00 |
| Turkey | 91 | 2714 | 0.48 | 0.61 | -0.13 | 0.05 | -2.65 | 0.01 |
| UK | 82 | 2747 | 0.49 | 0.33 | 0.16 | 0.05 | 2.90 | 0.00 |
| US | 192 | 2348 | 0.54 | 0.50 | 0.04 | 0.03 | 1.05 | 0.29 |

**Table B8** t-tests of means for impatience (average rho) with unequal variance between vaccination groups within each country (Refuser vs hesitant)

| **Refusers vs hesitant** | N. Hesitant | N. Accepters | Mean hesitant | Mean Accepters | Diff. | St. error | t-value | p-value |
| --- | --- | --- | --- | --- | --- | --- | --- | --- |
| Australia | 164 | 69 | 0.36 | 0.52 | -0.16 | 0.06 | -2.60 | 0.01 |
| Brazil | 52 | 49 | 0.42 | 0.38 | 0.04 | 0.09 | 0.45 | 0.64 |
| Chile | 65 | 43 | 0.23 | 0.25 | -0.03 | 0.08 | -0.35 | 0.73 |
| Croatia | 219 | 107 | 0.40 | 0.35 | 0.05 | 0.05 | 1.05 | 0.29 |
| France | 293 | 115 | 0.33 | 0.40 | -0.07 | 0.05 | -1.40 | 0.16 |
| India | 22 | 61 | 0.82 | 0.78 | 0.04 | 0.11 | 0.35 | 0.72 |
| Israel | 85 | 45 | 0.31 | 0.45 | -0.14 | 0.08 | -1.70 | 0.09 |
| Italy | 139 | 42 | 0.33 | 0.48 | -0.15 | 0.08 | -1.85 | 0.07 |
| Latvia | 200 | 72 | 0.40 | 0.42 | -0.02 | 0.06 | -0.30 | 0.77 |
| Lithuania | 167 | 55 | 0.34 | 0.37 | -0.03 | 0.06 | -0.45 | 0.66 |
| Norway | 52 | 16 | 0.37 | 0.66 | -0.29 | 0.12 | -2.45 | 0.02 |
| Russia | 659 | 611 | 0.34 | 0.36 | -0.02 | 0.02 | -0.90 | 0.37 |
| Singapore | 12 | 11 | 0.19 | 0.70 | -0.52 | 0.18 | -2.95 | 0.01 |
| Slovakia | 206 | 79 | 0.34 | 0.31 | 0.03 | 0.05 | 0.60 | 0.55 |
| Slovenia | 231 | 91 | 0.36 | 0.35 | 0.02 | 0.05 | 0.30 | 0.76 |
| South Africa | 453 | 335 | 0.35 | 0.38 | -0.03 | 0.03 | -1.10 | 0.26 |
| South Korea | 115 | 104 | 0.37 | 0.46 | -0.09 | 0.05 | -1.70 | 0.09 |
| Spain | 150 | 52 | 0.32 | 0.40 | -0.08 | 0.07 | -1.15 | 0.25 |
| Sweden | 115 | 64 | 0.27 | 0.50 | -0.24 | 0.07 | -3.50 | 0.00 |
| Turkey | 257 | 91 | 0.69 | 0.45 | 0.24 | 0.05 | 4.60 | 0.00 |
| UK | 229 | 82 | 0.37 | 0.50 | -0.13 | 0.06 | -2.20 | 0.03 |
| US | 464 | 192 | 0.45 | 0.58 | -0.13 | 0.04 | -3.35 | 0.00 |

**Table B9** t-tests of means for impatience (average rho) with unequal variance between vaccination groups within each country (Refuser vs accepters)

| **Refusers vs accepters** | N. Refuser | N. Hesitant | Mean Refuser | Mean hesitant | Diff. | St. error | t-value | p-value |
| --- | --- | --- | --- | --- | --- | --- | --- | --- |
| Australia | 164 | 2720 | 0.36 | 0.39 | -0.03 | 0.03 | -0.85 | 0.41 |
| Brazil | 52 | 2882 | 0.42 | 0.39 | 0.04 | 0.07 | 0.55 | 0.58 |
| Chile | 65 | 2876 | 0.23 | 0.26 | -0.04 | 0.04 | -0.80 | 0.42 |
| Croatia | 219 | 710 | 0.40 | 0.30 | 0.10 | 0.03 | 3.20 | 0.00 |
| France | 293 | 2708 | 0.33 | 0.36 | -0.03 | 0.03 | -1.20 | 0.22 |
| India | 22 | 3014 | 0.82 | 0.62 | 0.20 | 0.10 | 2.05 | 0.05 |
| Israel | 85 | 1354 | 0.31 | 0.39 | -0.07 | 0.05 | -1.60 | 0.11 |
| Italy | 139 | 2811 | 0.33 | 0.31 | 0.03 | 0.04 | 0.70 | 0.50 |
| Latvia | 200 | 795 | 0.40 | 0.35 | 0.05 | 0.03 | 1.40 | 0.16 |
| Lithuania | 167 | 764 | 0.34 | 0.31 | 0.04 | 0.03 | 1.05 | 0.29 |
| Norway | 52 | 945 | 0.37 | 0.27 | 0.10 | 0.06 | 1.60 | 0.12 |
| Russia | 659 | 1581 | 0.34 | 0.33 | 0.01 | 0.02 | 0.55 | 0.59 |
| Singapore | 12 | 970 | 0.19 | 0.40 | -0.21 | 0.10 | -2.10 | 0.06 |
| Slovakia | 206 | 681 | 0.34 | 0.32 | 0.03 | 0.03 | 0.85 | 0.41 |
| Slovenia | 231 | 720 | 0.36 | 0.30 | 0.06 | 0.03 | 1.90 | 0.06 |
| South Africa | 453 | 2084 | 0.35 | 0.42 | -0.07 | 0.02 | -3.50 | 0.00 |
| South Korea | 115 | 2728 | 0.37 | 0.36 | 0.01 | 0.03 | 0.40 | 0.70 |
| Spain | 150 | 3038 | 0.32 | 0.28 | 0.04 | 0.03 | 1.15 | 0.24 |
| Sweden | 115 | 1302 | 0.27 | 0.30 | -0.03 | 0.04 | -0.90 | 0.36 |
| Turkey | 257 | 2714 | 0.69 | 0.60 | 0.09 | 0.03 | 3.00 | 0.00 |
| UK | 229 | 2747 | 0.37 | 0.35 | 0.02 | 0.03 | 0.85 | 0.39 |
| US | 464 | 2348 | 0.45 | 0.51 | -0.07 | 0.02 | -2.90 | 0.00 |

**Table B9** t-tests of means for impatience (average rho) with unequal variance between vaccination groups within each country (Hesitant vs accepters)

| **Hesitant vs accepters** | N. Hesitant | N. Accepters | Mean hesitant | Mean Accepters | Diff. | St. error | t-value | p-value |
| --- | --- | --- | --- | --- | --- | --- | --- | --- |
| Australia | 69 | 2720 | 0.52 | 0.39 | 0.13 | 0.05 | 2.50 | 0.02 |
| Brazil | 49 | 2882 | 0.38 | 0.39 | 0.00 | 0.06 | -0.05 | 0.94 |
| Chile | 43 | 2876 | 0.25 | 0.26 | -0.01 | 0.06 | -0.15 | 0.88 |
| Croatia | 107 | 710 | 0.35 | 0.30 | 0.05 | 0.04 | 1.25 | 0.21 |
| France | 115 | 2708 | 0.40 | 0.36 | 0.04 | 0.04 | 0.85 | 0.40 |
| India | 61 | 3014 | 0.78 | 0.62 | 0.16 | 0.06 | 2.70 | 0.01 |
| Israel | 45 | 1354 | 0.45 | 0.39 | 0.06 | 0.07 | 0.95 | 0.35 |
| Italy | 42 | 2811 | 0.48 | 0.31 | 0.17 | 0.07 | 2.40 | 0.02 |
| Latvia | 72 | 795 | 0.42 | 0.35 | 0.07 | 0.05 | 1.30 | 0.20 |
| Lithuania | 55 | 764 | 0.37 | 0.31 | 0.06 | 0.06 | 1.15 | 0.26 |
| Norway | 16 | 945 | 0.66 | 0.27 | 0.40 | 0.10 | 3.85 | 0.00 |
| Russia | 611 | 1581 | 0.36 | 0.33 | 0.03 | 0.02 | 1.60 | 0.11 |
| Singapore | 11 | 970 | 0.70 | 0.40 | 0.31 | 0.15 | 2.10 | 0.06 |
| Slovakia | 79 | 681 | 0.31 | 0.32 | -0.01 | 0.05 | -0.10 | 0.91 |
| Slovenia | 91 | 720 | 0.35 | 0.30 | 0.04 | 0.05 | 1.00 | 0.33 |
| South Africa | 335 | 2084 | 0.38 | 0.42 | -0.04 | 0.03 | -1.65 | 0.10 |
| South Korea | 104 | 2728 | 0.46 | 0.36 | 0.10 | 0.04 | 2.55 | 0.01 |
| Spain | 52 | 3038 | 0.40 | 0.28 | 0.12 | 0.06 | 2.00 | 0.05 |
| Sweden | 64 | 1302 | 0.50 | 0.30 | 0.20 | 0.06 | 3.45 | 0.00 |
| Turkey | 91 | 2714 | 0.45 | 0.60 | -0.15 | 0.04 | -3.40 | 0.00 |
| UK | 82 | 2747 | 0.50 | 0.35 | 0.15 | 0.05 | 2.90 | 0.00 |
| US | 192 | 2348 | 0.58 | 0.51 | 0.06 | 0.03 | 1.85 | 0.06 |
