# Supplementary material for: Time preferences and COVID-19 vaccination uptake
Source: Eur J Health Econ. 2025 Jun 14;27(1):47–63. doi: 10.1007/s10198-025-01801-7 (PMC12929247; doi:10.1007/s10198-025-01801-7)
Supplement: Supplementary file 2 — Supplementary Material 2 [file 10198_2025_1801_MOESM2_ESM.docx]

**Supplementary Materials A**

1. **Choice list methodology**

***Table A1*** *Screenshot of the choice lists*

*Choice list 1: “Imagine that you have won a lottery and have to choose whether to receive the prize immediately or in three months. Which of the options reported below do you prefer - Option A or Option B?”*

|  | **Option A** | **Option B** | **Implied monthly discount rate when switching from B to A in each row (if utility is linear)** |
| --- | --- | --- | --- |
| 1. | □ $5 today | □ $100 in 3 months | 1.229626 |
| 2. | □ $10 today | □ $100 in 3 months | 0.863422 |
| 3. | □ $15 today | □ $100 in 3 months | 0.693147 |
| 4. | □ $20 today | □ $100 in 3 months | 0.58099 |
| 5. | □ $25 today | □ $100 in 3 months | 0.497218 |
| 6. | □ $30 today | □ $100 in 3 months | 0.430328 |
| 7. | □ $35 today | □ $100 in 3 months | 0.374643 |
| 8. | □ $40 today | □ $100 in 3 months | 0.326943 |
| 9. | □ $45 today | □ $100 in 3 months | 0.285222 |
| 10. | □ $50 today | □ $100 in 3 months | 0.248147 |
| 11. | □ $55 today | □ $100 in 3 months | 0.214786 |
| 12. | □ $60 today | □ $100 in 3 months | 0.184462 |
| 13. | □ $65 today | □ $100 in 3 months | 0.156668 |
| 14. | □ $70 today | □ $100 in 3 months | 0.131014 |
| 15. | □ $75 today | □ $100 in 3 months | 0.107195 |
| 16. | □ $80 today | □ $100 in 3 months | 0.084964 |
| 17. | □ $85 today | □ $100 in 3 months | 0.064124 |
| 18. | □ $90 today | □ $100 in 3 months | 0.04451 |
| 19. | □ $95 today | □ $100 in 3 months | 0.025987 |
| 20. | □ $100 today | □ $100 in 3 months | 0.008439 |

*Choice list 2: “Imagine now that you have to choose whether to receive the prize in three months or in six months. Which of the options reported below do you prefer - Option A or Option B?”*

|  | **Option A** | **Option B** | **Implied monthly discount rate when switching from B to A in each row (if utility is linear)** |
| --- | --- | --- | --- |
| 1. | □ $5 in 3 months | □ $100 in 6 months | 1.229626 |
| 2. | □ $10 in 3 months | □ $100 in 6 months | 0.863422 |
| 3. | □ $15 in 3 months | □ $100 in 6 months | 0.693147 |
| 4. | □ $20 in 3 months | □ $100 in 6 months | 0.58099 |
| 5. | □ $25 in 3 months | □ $100 in 6 months | 0.497218 |
| 6. | □ $30 in 3 months | □ $100 in 6 months | 0.430328 |
| 7. | □ $35 in 3 months | □ $100 in 6 months | 0.374643 |
| 8. | □ $40 in 3 months | □ $100 in 6 months | 0.326943 |
| 9. | □ $45 in 3 months | □ $100 in 6 months | 0.285222 |
| 10. | □ $50 in 3 months | □ $100 in 6 months | 0.248147 |
| 11. | □ $55 in 3 months | □ $100 in 6 months | 0.214786 |
| 12. | □ $60 in 3 months | □ $100 in 6 months | 0.184462 |
| 13. | □ $65 in 3 months | □ $100 in 6 months | 0.156668 |
| 14. | □ $70 in 3 months | □ $100 in 6 months | 0.131014 |
| 15. | □ $75 in 3 months | □ $100 in 6 months | 0.107195 |
| 16. | □ $80 in 3 months | □ $100 in 6 months | 0.084964 |
| 17. | □ $85 in 3 months | □ $100 in 6 months | 0.064124 |
| 18. | □ $90 in 3 months | □ $100 in 6 months | 0.04451 |
| 19. | □ $95 in 3 months | □ $100 in 6 months | 0.025987 |
| 20. | □ $100 in 3 months | □ $100 in 6 months | 0.008439 |

1. **Derivation of the parameter estimates of the quasi-hyperbolic discounting model (**$\beta$ **and** $\delta$**).**

In the first choice list, respondents had to choose between a varying immediate reward and a reward of $100 in t=3 months. According to the quasi-hyperbolic discounting model with linear utility, the obtained indifference point *x_1_* can be evaluated as follows:

$x_{1}=\beta\delta^{3}\times100$. (A1)

Solving for $\beta$ yields:

$\beta=(100\delta^{3})/x_{1}$. (A2)

We now use the second choice list to estimate the discount rate delta, which is then plugged into Eq. A2 to solve for $\beta$. That is, the second choice list involved a choice between a varying reward in 3 months and a reward of $100 in 6 months. Indifference at *x_2_* in 3 months can be evaluated by:

$\beta\delta^{3}\times x_{2}=\beta\delta^{6}\times100$. (A3)

Because $\beta$ cancels out here, we can solve for $\delta$:

$\delta=\left( \frac{x_{2}}{100} \right)^{1/3}$. (A4)

1. **Identification of the revealed vaccination status from the VaxPref database.**

To collect information about respondents’ COVID-19 vaccination status, the VaxPref questionnaire asked the following question to respondents:

1. Which of the following best represents your COVID-19 vaccination status?

- I already received the booster (3^rd^ dose)
- I already received the 2 vaccine shots and waiting for the booster
- I already received the 2 vaccine shots
- I already received the 1st shot and I am waiting for the 2^nd^ one
- I already received the 1st shot and not intend to get more doses
- I want to receive the vaccine, but it is difficult for me to get it
- I am planning to get vaccinated
- I will be vaccinated only when I am sure it is effective
- I will be vaccinated only when I know more about the possible long-term side-effects
- I have no intention to get the vaccine
- Due to a health condition, the vaccine is strongly not recommended for me
- Other, please specify in the box below


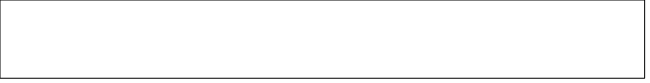


1. **Data and variables included in the analysis**

In the next Table, we report and describe the explanatory variables used in the analysis. All the data are retrieved from the VaxPref database [1].

***Table A2*** *Descriptive statistics of the explanatory variables included in the analysis*

| Explanatory variable | Definition | Modality |
| --- | --- | --- |
| Rho2 | Individual impatience (discount rate) under the quasi-hyperbolic (or beta-delta) discounting model | Continuous variable:  Mean = 0.39  Min = 0  Max = 1.23 |
| Beta | Impulsivity (Present bias - β) under the quasi-hyperbolic (or beta-delta) discounting model | Continuous variable:  Mean = 2.04  Min = 0.03  Max = 40.0 |
| Average rho | Individual average impatience (discount rate) considering the constant discounting model | Continuous variable:  Mean = 0.39  Min = 0  Max = 1.23 |
| Risk in health | Willingness to take risk in the health domain, retrieved from the direct method proposed by Yang et al. [61]. Respondents were asked: *How would you rate your willingness to take risks with your health? Please write a number between 0 and 10 in the box below, where 0 means ‘Not at all prepared to take risks’ and 10 means ‘Fully prepared to take risks’.”* | Ordinal variable:  Mean = 3.69  Min = 0  Max = 10 |
| Female | Gender of the respondent | Dummy variable:  Female = 1 (51.3%)  Other = 0 (48.7%) |
| Age | Age of the respondent at the time of the survey | Continuous variable:  Mean = 45.56  Min = 18  Max = 99 |
| Bachelor | Highest educational attainment at the time of the survey | Dummy variable:  Bachelor and over = 1 (50.5%)  Other = 0 (49.5%) |
| High income | Household income at the time of the survey, obtained following the OECD income classes classification, where high income groups are defined as those >200% of the median national income | Dummy variable:  High income = 1 (25.5%)  Other = 0 (74.5%) |
| Children | Whether the respondent has one or more children at the time of the survey | Dummy variable:  Yes = 1 (68.5%)  No =0 (32.5%) |

The correlation analysis among the explanatory variables is presented in the following correlation matrix.

***Table A3*** *Correlation matrix among the explanatory variables used in the analysis*

|  | Rho2 | Beta | Average rho | Risk in health | Female | Age | Bachelor | High Income | Children |
| --- | --- | --- | --- | --- | --- | --- | --- | --- | --- |
| Rho2 | 1 |  |  |  |  |  |  |  |  |
| Beta | 0.36 | 1 |  |  |  |  |  |  |  |
| Average rho | 0.93 | 0.12 | 1 |  |  |  |  |  |  |
| Risk in health | 0.19 | 0.02 | 0.21 | 1 |  |  |  |  |  |
| Female | 0.01 | 0.01 | 0.01 | -0.12 | 1 |  |  |  |  |
| Age | -0.14 | -0.06 | -0.14 | -0.22 | 0.04 | 1 |  |  |  |
| Bachelor | -0.03 | -0.02 | -0.03 | 0.08 | -0.02 | -0.04 | 1 |  |  |
| High Income | -0.01 | 0.00 | -0.02 | 0.09 | -0.07 | -0.08 | 0.25 | 1 |  |
| Children | 0.02 | -0.01 | 0.02 | -0.03 | 0.07 | 0.32 | 0.05 | 0.12 | 1 |

***Figure A1*** *Predicted differences in patience (rho2) across the identified areas*


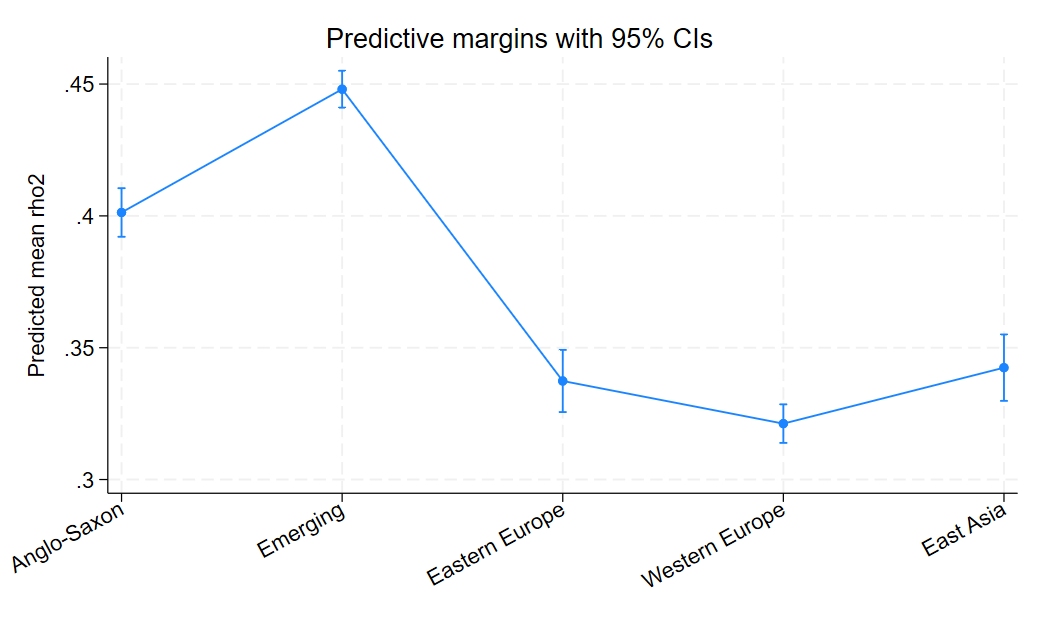


Note: The predictive margins are estimated using a GLM model. The GLM model was preferred to account for the different sample sizes and the unequal variances across areas

1. **Robustness checks**

To test the robustness of our findings, we modified the threshold used to define the vaccine accepters group. In the original analysis, respondents who chose the vaccination option 10 or more times were classified as vaccine accepters. In the robustness check, we increased the threshold to 12, considering only respondents who consistently chose the vaccination option across all scenarios as vaccine accepters. Table A4 presents a comparison of the results under these two thresholds. The findings remain consistent with those of the original specification.

***Table A4*** *Multinomial logistic regression on stated vaccination status* – robustness checks

|  |  | Stated Vaccination status (baseline) | | | | |  | Stated Vaccination Status (robustness check) | | | | |
| --- | --- | --- | --- | --- | --- | --- | --- | --- | --- | --- | --- | --- |
|  |  | Hyperbolic | |  | Constant | |  | Hyperbolic | |  | Constant | |
|  |  | (1) | (2) |  | (3) | (4) |  | (5) | (6) |  | (7) | (8) |
| Refuser | rho2 | -0.32** | -0.25*** |  | - | - |  | -0.25** | -0.18*** |  | - | - |
|  |  | (.127) | (0.03) |  |  |  |  | (0.13) | (0.03) |  |  |  |
|  | beta | 0.01* | 0.01*** |  | - | - |  | .007 | 0.01** |  | - | - |
|  |  | (<0.01) | (<0.01) |  |  |  |  | (0.01) | (0.003) |  |  |  |
|  | Average rho | - | - |  | -0.32*** | -.25*** |  | - | - |  | -0.25** | -0.18*** |
|  |  |  |  |  | (0.13) | (.03) |  |  |  |  | (0.12) | (0.03) |
|  | cons | -1.19*** | -1.87*** |  | -1.17*** | -1.85*** |  | -1.028*** | -1.729*** |  | -1.01*** | -1.72*** |
|  |  | (0.17) | (0.06) |  | (0.17) | (0.06) |  | (0.18) | (0.06) |  | (0.18) | (0.06) |
| Hesitant | rho2 | 0.103 | 0.15*** |  | - | - |  | 0.24*** | 0.28*** |  | - | - |
|  |  | (0.07) | (0.03) |  |  |  |  | (0.06) | (0.025) |  |  |  |
|  | beta | -0.01*** | -0.01** |  | - | - |  | -0.01*** | -0.01*** |  | - | - |
|  |  | (<0.01) | (<0.01) |  |  |  |  | (0.003) | (0.002) |  |  |  |
|  | Average rho | - | - |  | 0.10 | 0.15*** |  | - | - |  | 0.24*** | 0.28*** |
|  |  |  |  |  | (0.07) | (0.03) |  |  |  |  | (.06) | (.02) |
|  | cons | -1.12*** | -1.40*** |  | -1.14*** | -1.44*** |  | -0.57*** | -0.85*** |  | -0.60*** | -0.87*** |
|  |  | (0.02) | (0.05) |  | (0.12) | (0.05) |  | (0.10) | (0.04) |  | (0.10) | (0.04) |
| Vaccinated | (Baseline) | - | - |  | - | - |  | - | - |  | - | - |
|  | Country FE | No | Yes |  | No | Yes |  | No | Yes |  | No | Yes |
|  | Observations | 49227 | 49227 |  | 49227 | 49227 |  | 49227 | 49227 |  | 49227 | 49227 |
|  | Pseudo R2 | <0.01 | 0.07 |  | <0.01 | 0.07 |  | <0.01 | 0.06 |  | <0.01 | 0.06 |
|  | Log-Likelihood | -45403.879 | -42330.819 |  | -45402.35 | -42329.48 |  | -49450.448 | -46586.183 |  | -49444.55 | -46581.22 |
|  | Akaike's Crit | 90819.758 | 84757.638 |  | 90812.69 | 84750.96 |  | 98912.896 | 93268.367 |  | 98897.09 | 93254.43 |
|  | Bayesian Crit | 90872.583 | 85180.24 |  | 90847.91 | 85155.95 |  | 98965.721 | 93690.968 |  | 98932.31 | 93659.43 |

Note: In this Table we report the robustness check we conducted for the stated preference data by considering vaccine accepters only those who accepted the vaccine in all the 12 choice tasks (columns (5)-(8). *** p<.01, ** p<.05, * p<.1. Standard errors are in parentheses. In column (1), (3), (5) and (7) standard errors are clustered at the country level.

1. **Time preferences and attitudes towards vaccinations – Baseline regressions**

In the following Tables, we report the outcomes of our baseline multinomial logistic regressions accounting for hyperbolic and constant discounting in both revealed and stated preferences.

***6.1 Revealed preferences***

***Table A5*** *Basic multinomial logistic regressions at the country level – Anglo-Saxon and East Asian countries*

|  |  | Australia | UK | USA |  | Singapore | South Korea |
| --- | --- | --- | --- | --- | --- | --- | --- |
|  |  |  |  |  |  |  |  |
| Refuser | rho2 | -0.22 | <0.01 | -.42*** |  | -1.42 | -.44*** |
|  |  | (0.20) | (0.19) | (0.12) |  | (1.06) | (0.13) |
|  | beta | -0.02 | 0.01 | .03*** |  | -0.33 | 0.01 |
|  |  | (0.03) | (0.02) | (0.01) |  | (0.63) | (0.01) |
|  | cons | -2.7*** | -2.5*** | -1.5*** |  | -3.67*** | -1.37*** |
|  |  | (0.10) | (0.09) | (0.07) |  | (0.68) | (0.07) |
| Hesitant | rho2 | .66** | 1.06*** | 0.25 |  | 1.31** | -0.17 |
|  |  | (0.26) | (0.25) | (0.16) |  | (0.62) | (0.14) |
|  | beta | <0.01 | -.12* | -.05* |  | -0.19 | -0.01 |
|  |  | (0.02) | (0.07) | (0.03) |  | (0.25) | (0.01) |
|  | cons | -3.97*** | -3.77*** | -2.56*** |  | -4.89*** | -1.74*** |
|  |  | (0.18) | (0.17) | (0.11) |  | (0.54) | (0.08) |
| Vaccinated | (baseline) | - | - | - |  |  |  |
|  | Observations | 2953 | 3058 | 3004 |  | 993 | 2872 |
|  | Pseudo R2 | 0.01 | 0.01 | 0.01 |  | 0.03 | <0.01 |
|  | ll | -952.03 | -1175.57 | -1959.87 |  | -120.89 | -2217.6 |
|  | Akaike's Crit | 1916.06 | 2363.14 | 3931.73 |  | 253.77 | 4447.2 |
|  | Bayesian Crit | 1952 | 2399.29 | 3967.78 |  | 283.18 | 4482.97 |
| Standard errors are in parentheses | | | | | | | |
| *** p<.01, ** p<.05, * p<.1 | | | | | | | |

***Table A6*** *Basic multinomial logistic regressions at the country level – Eastern European countries*

|  |  | Croatia | Latvia | Lithuania | Slovakia | Slovenia |
| --- | --- | --- | --- | --- | --- | --- |
|  |  |  |  |  |  |  |
| Refuser | rho2 | 0.55*** | 0.33* | 0.26 | 0.13 | 0.43** |
|  |  | (0.19) | (0.19) | (0.22) | (0.21) | (0.19) |
|  | beta | 0.02 | -0.02 | -0.02 | 0.01 | -0.03* |
|  |  | (0.01) | (0.01) | (0.02) | (0.01) | (0.02) |
|  | cons | -1.42*** | -1.47*** | -1.56*** | -1.26*** | -1.22*** |
|  |  | (0.10) | (0.11) | (0.11) | (0.10) | (0.10) |
| Hesitant | rho2 | 0.35 | 0.29 | 0.51 | 0.14 | 0.28 |
|  |  | (0.26) | (0.29) | (0.33) | (0.31) | (0.28) |
|  | beta | 0.01 | -0.02 | -0.06 | <0.01 | <0.01 |
|  |  | (0.02) | (0.03) | (0.04) | (0.02) | (0.02) |
|  | cons | -2.02*** | -2.46*** | -2.70*** | -2.19*** | -2.16*** |
|  |  | (0.13) | (0.16) | (0.19) | (0.15) | (0.14) |
| Vaccinated | (baseline) |  |  |  |  |  |
|  | Observations | 1036 | 1067 | 986 | 966 | 1042 |
|  | Pseudo R2 | 0.01 | <0.01 | 0.004 | <0.01 | <0.01 |
|  | ll | -842.08 | -760.88 | -647.59 | -753.61 | -832.49 |
|  | Akaike's Crit | 1696.16 | 1533.77 | 1307.18 | 1519.23 | 1676.97 |
|  | Bayesian Crit | 1725.82 | 1563.6 | 1336.54 | 1548.47 | 1706.67 |
| Standard errors are in parentheses | | | | | | |
| *** p<.01, ** p<.05, * p<.1 | | | | | | |

***Table A7*** *Basic multinomial logistic regressions at the country level – Western European countries*

|  |  | France | Israel | Italy | Norway | Spain | Sweden |
| --- | --- | --- | --- | --- | --- | --- | --- |
|  |  |  |  |  |  |  |  |
| Refuser | rho2 | -0.17 | -0.58* | 0.13 | 0.7** | 0.2 | -0.21 |
|  |  | (0.15) | (0.31) | (0.22) | (0.33) | (0.21) | (0.27) |
|  | beta | 0.01 | -0.01 | 0.01 | -0.06 | 0.02* | -0.03 |
|  |  | (0.01) | (0.04) | (0.02) | (0.06) | (0.01) | (0.05) |
|  | cons | -2.19*** | -2.56*** | -3.06*** | -3.02*** | -3.13*** | -2.32*** |
|  |  | (0.08) | (0.14) | (0.11) | (0.19) | (0.11) | (0.12) |
| Hesitant | rho2 | 0.14 | 0.4 | 0.8** | 1.73*** | 0.43 | 0.98*** |
|  |  | (0.22) | (0.33) | (0.33) | (0.52) | (0.33) | (0.28) |
|  | beta | <0.01 | -0.07 | 0.01 | 0.02 | 0.02 | <0.01 |
|  |  | (0.02) | (0.08) | (0.02) | (0.03) | (0.02) | (0.02) |
|  | cons | -3.2*** | -3.46*** | -4.54*** | -4.91*** | -4.28*** | -3.4*** |
|  |  | (0.12) | (0.22) | (0.22) | (0.41) | (0.18) | (0.18) |
| Vaccinated | (baseline) |  |  |  |  |  |  |
|  | Observations | 3116 | 1484 | 2992 | 1013 | 3240 | 1481 |
|  | Pseudo R2 | <0.01 | 0.01 | <0.01 | 0.03 | 0.01 | 0.01 |
|  | ll | -1450.98 | -520.78 | -777.62 | -277.75 | -865.75 | -654.59 |
|  | Akaike's Crit | 2913.97 | 1053.57 | 1567.25 | 567.5 | 1743.5 | 1321.18 |
|  | Bayesian Crit | 2950.23 | 1085.38 | 1603.27 | 597.03 | 1780 | 1352.99 |
| Standard errors are in parentheses | | | | |  |  |  |
| *** p<.01, ** p<.05, * p<.1 | | | | |  |  |  |

***Table A8*** *Basic multinomial logistic regressions at the country level – Emerging countries*

|  |  | Brazil | Chile | India | Russia | South Africa | Turkey |
| --- | --- | --- | --- | --- | --- | --- | --- |
|  |  |  |  |  |  |  |  |
| Refuser | rho2 | 0.12 | -0.33 | 0.9* | -0.01 | -0.44*** | 0.44*** |
|  |  | (0.32) | (0.37) | (0.47) | (0.12) | (0.13) | (0.14) |
|  | beta | -0.05 | -0.01 | 0.01 | <0.01 | 0.01 | -0.08*** |
|  |  | (0.04) | (0.03) | (0.03) | (0.01) | (0.01) | (0.03) |
|  | cons | -3.97*** | -3.68*** | -5.59*** | -0.87*** | -1.37*** | -2.49*** |
|  |  | (0.19) | (0.15) | (0.43) | (0.06) | (0.07) | (0.12) |
| Hesitant | rho2 | -0.06 | 0.12 | 0.79*** | 0.21* | -0.17 | -0.73*** |
|  |  | (0.35) | (0.40) | (0.27) | (0.12) | (0.14) | (0.26) |
|  | beta | 0.03 | -0.04 | -0.16 | <0.01 | -0.01 | 0.02 |
|  |  | (0.02) | (0.04) | (0.11) | (0.01) | (0.01) | (0.02) |
|  | cons | -4.14*** | -4.15*** | -4.21*** | -1.02*** | -1.74*** | -3.06*** |
|  |  | (0.19) | (0.18) | (0.25) | (0.06) | (0.08) | (0.16) |
| Vaccinated | (baseline) |  |  |  |  |  |  |
|  | Observations | 2983 | 2984 | 3097 | 2851 | 2872 | 3062 |
|  | Pseudo R2 | <0.01 | <0.01 | 0.02 | <0.01 | <0.01 | 0.01 |
|  | ll | -509.12 | -535.28 | -421.97 | -2836.65 | -2217.6 | -1268.76 |
|  | Akaike's Crit | 1030.24 | 1082.57 | 855.94 | 5685.29 | 4447.2 | 2549.51 |
|  | Bayesian Crit | 1066.24 | 1118.58 | 892.16 | 5721.02 | 4482.97 | 2585.67 |
| Standard errors are in parentheses | | | | |  |  |  |
| *** p<.01, ** p<.05, * p<.1 | | | | |  |  |  |

***6.2 Stated preferences***

***Table A9*** *Basic multinomial logistic regressions at the country level – Anglo-Saxon and East Asian countries*

|  |  | Australia | UK | USA |  | Singapore | South Korea |
| --- | --- | --- | --- | --- | --- | --- | --- |
|  |  |  |  |  |  |  |  |
| Refuser | rho2 | -0.46*** | -0.33* | -0.91*** |  | -0.52** | -0.21 |
|  |  | (0.16) | (0.18) | (0.12) |  | (0.26) | (0.21) |
|  | beta | <0.01 | 0.01 | 0.04*** |  | -0.01 | 0.01 |
|  |  | (0.02) | (0.02) | (0.01) |  | (0.03) | (0.01) |
|  | cons | -1.78*** | -1.78*** | -1.03*** |  | -1.59*** | -0.53*** |
|  |  | (0.08) | (0.08) | (0.07) |  | (0.13) | (0.09) |
| Hesitant | rho2 | 0.08 | 0.5*** | -0.02 |  | -0.16 | 0.25 |
|  |  | (0.11) | (0.12) | (0.11) |  | (0.19) | (0.22) |
|  | beta | -0.02 | -0.02 | -0.01 |  | -0.01 | <0.01 |
|  |  | (0.02) | (0.01) | (0.01) |  | (0.02) | (0.02) |
|  | cons | -1.37*** | -1.4*** | -1.25*** |  | -1.11*** | -1.13*** |
|  |  | (0.06) | (0.06) | (0.07) |  | (0.11) | (0.11) |
| Vaccinated | (baseline) | - | - | - |  | - | - |
|  | Observations | 2953 | 3058 | 3004 |  | 993 | 966 |
|  | Pseudo R2 | <0.01 | .01 | .01 |  | <0.01 | <0.01 |
|  | ll | -2300.77 | -2478.45 | -2640.82 |  | -825.91 | -974.91 |
|  | Akaike's Crit | 4613.54 | 4968.91 | 5293.65 |  | 1663.82 | 1961.82 |
|  | Bayesian Crit | 4649.48 | 5005.06 | 5329.7 |  | 1693.22 | 1991.06 |
| Standard errors are in parentheses | | | | | | | |
| *** p<.01, ** p<.05, * p<.1 | | | | | | | |

***Table A10*** *Basic multinomial logistic regressions at the country level – Eastern European countries*

|  |  | Croatia | Latvia | Lithuania | Slovakia | Slovenia |
| --- | --- | --- | --- | --- | --- | --- |
|  |  |  |  |  |  |  |
| Refuser | rho2 | 0.12 | -0.57*** | -0.68** | 0.28 | 0.11 |
|  |  | (0.18) | (0.12) | (0.28) | (0.2) | (0.13) |
|  | beta | 0.02 | 0.02 | 0.03 | <0.01 | 0.01 |
|  |  | (0.01) | (0.01) | (0.02) | (0.02) | (0.01) |
|  | cons | -0.37*** | -0.81*** | -3.29*** | 0.83*** | -1.58*** |
|  |  | (0.09) | (0.06) | (0.17) | (0.1) | (0.07) |
| Hesitant | rho2 | 0.26 | 0.09 | 0.11 | 0.88*** | -0.18 |
|  |  | (0.2) | (0.1) | (0.1) | (0.2) | (0.12) |
|  | beta | <0.01 | <0.01 | -0.03** | -0.02 | <0.01 |
|  |  | (0.02) | (0.01) | (0.01) | (0.02) | (0.01) |
|  | cons | -0.76*** | -0.77*** | -1.61*** | 0.73*** | -1.07*** |
|  |  | (0.1) | (0.06) | (0.08) | (0.1) | (0.06) |
| Vaccinated | (baseline) | - | - | - | - | - |
|  | Observations | 1036 | 3116 | 3097 | 1484 | 2992 |
|  | Pseudo R2 | <0.01 | <0.01 | <0.01 | .01 | <0.01 |
|  | ll | -1098.29 | -3128.45 | -1700.25 | -1497.77 | -2651.32 |
|  | Akaike's Crit | 2208.57 | 6268.91 | 3412.5 | 3007.55 | 5314.63 |
|  | Bayesian Crit | 2238.23 | 6305.17 | 3448.73 | 3039.36 | 5350.65 |
| Standard errors are in parentheses | | | | | | |
| *** p<.01, ** p<.05, * p<.1 | | | | | | |

***Table A11*** *Basic multinomial logistic regressions at the country level – Western European countries*

|  |  | France | Israel | Italy | Norway | Spain | Sweden |
| --- | --- | --- | --- | --- | --- | --- | --- |
|  |  |  |  |  |  |  |  |
| Refuser | rho2 | -0.57*** | -0.68** | 0.28 | 0.11 | 0.09 | 0.27 |
|  |  | (0.12) | (0.28) | (0.2) | (0.13) | (0.18) | (0.19) |
|  | beta | 0.02 | 0.03 | <0.01 | 0.01 | 0.01 | -0.01 |
|  |  | (0.01) | (0.02) | (0.02) | (0.01) | (0.01) | (0.01) |
|  | cons | -0.81*** | -3.29*** | 0.83*** | -1.58*** | 0.25*** | -0.07 |
|  |  | (0.06) | (0.17) | (0.1) | (0.07) | (0.09) | (0.09) |
| Hesitant | rho2 | 0.09 | 0.11 | 0.88*** | -0.18 | 0.36* | 0.12 |
|  |  | (0.1) | (0.1) | (0.2) | (0.12) | (0.21) | (0.23) |
|  | beta | <0.01 | -0.03** | -0.02 | <0.01 | <0.01 | 0.01 |
|  |  | (0.01) | (0.01) | (0.02) | (0.01) | (0.02) | (0.02) |
|  | cons | -0.77*** | -1.61*** | 0.73*** | -1.07*** | -0.65*** | -0.72*** |
|  |  | (0.06) | (0.08) | (0.1) | (0.06) | (0.11) | (0.11) |
| Vaccinated | (baseline) | - | - | - | - | - | - |
|  | Observations | 3116 | 3097 | 1484 | 2992 | 1067 | 986 |
|  | Pseudo R2 | <0.01 | <0.01 | .01 | <0.01 | <0.01 | <0.01 |
|  | ll | -3128.45 | -1700.25 | -1497.77 | -2651.32 | -1114.99 | -1042.36 |
|  | Akaike's Crit | 6268.91 | 3412.5 | 3007.55 | 5314.63 | 2241.98 | 2096.72 |
|  | Bayesian Crit | 6305.17 | 3448.73 | 3039.36 | 5350.65 | 2271.82 | 2126.08 |
| Standard errors are in parentheses | | | | |  |  |  |
| *** p<.01, ** p<.05, * p<.1 | | | | |  |  |  |

***Table A12*** *Basic multinomial logistic regressions at the country level – Emerging countries*

|  |  | Brazil | Chile | India | Russia | South Africa | Turkey |
| --- | --- | --- | --- | --- | --- | --- | --- |
|  |  |  |  |  |  |  |  |
| Refuser | rho2 | 0.12 | -0.33 | 0.9* | -0.01 | -0.44*** | 0.44*** |
|  |  | (0.32) | (0.37) | (0.47) | (0.12) | (0.13) | (0.14) |
|  | beta | -0.05 | -0.01 | 0.01 | <0.01 | 0.01 | -0.08*** |
|  |  | (0.04) | (0.03) | (0.03) | (0.01) | (0.01) | (0.03) |
|  | cons | -3.97*** | -3.68*** | -5.59*** | -0.87*** | -1.37*** | -2.49*** |
|  |  | (0.19) | (0.15) | (0.43) | (0.06) | (0.07) | (0.12) |
| Hesitant | rho2 | -0.06 | 0.12 | 0.79*** | 0.21* | -0.17 | -0.73*** |
|  |  | (0.35) | (0.40) | (0.27) | (0.12) | (0.14) | (0.26) |
|  | beta | 0.03 | -0.04 | -0.16 | <0.01 | -0.01 | 0.02 |
|  |  | (0.02) | (0.04) | (0.11) | (0.01) | (0.01) | (0.02) |
|  | cons | -4.14*** | -4.15*** | -4.21*** | -1.02*** | -1.74*** | -3.06*** |
|  |  | (0.19) | (0.18) | (0.25) | (0.06) | (0.08) | (0.16) |
| Vaccinated | (baseline) |  |  |  |  |  |  |
|  | Observations | 2983 | 2984 | 3097 | 2851 | 2872 | 3062 |
|  | Pseudo R2 | <0.01 | <0.01 | 0.02 | <0.01 | <0.01 | 0.01 |
|  | ll | -509.12 | -535.28 | -421.97 | -2836.65 | -2217.6 | -1268.76 |
|  | Akaike's Crit | 1030.24 | 1082.57 | 855.94 | 5685.29 | 4447.2 | 2549.51 |
|  | Bayesian Crit | 1066.24 | 1118.58 | 892.16 | 5721.02 | 4482.97 | 2585.67 |
| Standard errors are in parentheses | | | | |  |  |  |
| *** p<.01, ** p<.05, * p<.1 | | | | |  |  |  |

1. **Time preferences and attitudes towards vaccinations – Full regression**

In this section, we present the results of the full multinomial logistic regressions obtained using the current vaccination status as the dependent variable and assuming constant discounting. The coefficients correspond to those shown in Figure 3.

- 1. **Revealed preferences – constant discounting**

***Table A13*** *Multinomial logistic regression with constant discounting – Anglo-Saxon and East Asian countries*

|  |  | Australia | UK | USA |  | Singapore | South Korea |
| --- | --- | --- | --- | --- | --- | --- | --- |
|  |  |  |  |  |  |  |  |
| Refuser | Average Rho | -0.10 | -0.24 | -0.42*** |  | -0.57 | 0.08 |
|  |  | (0.21) | (0.18) | (0.13) |  | (1.00) | (0.26) |
|  | Risk in health | -0.05* | 0.03 | -0.01 |  | -0.49*** | -0.05 |
|  |  | (0.03) | (0.03) | (0.02) |  | (0.19) | (0.04) |
|  | Female | 0.37** | -0.06 | 0.32*** |  | -0.42 | -0.18 |
|  |  | (0.17) | (0.14) | (0.11) |  | (0.69) | (0.19) |
|  | Age | 0.05 | 0.09*** | 0.04* |  | 0.46** | -0.08** |
|  |  | (0.03) | (0.03) | (0.02) |  | (0.23) | (0.04) |
|  | Age^2^ | <0.01** | <0.01*** | <0.01*** |  | -0.01** | <0.01* |
|  |  | (0.00) | (0.00) | (0.00) |  | (0.00) | (0.00) |
|  | Bachelor | -0.90*** | -0.50*** | -1.20*** |  | -1.87** | -0.38* |
|  |  | (0.20) | (0.16) | (0.13) |  | (0.87) | (0.21) |
|  | High income | -0.56* | -0.40 | -1.32*** |  | 2.32*** | -0.88*** |
|  |  | (0.29) | (0.27) | (0.36) |  | (0.80) | (0.28) |
|  | With children | 0.06 | -0.07 | -0.17 |  | -2.11** | -0.79*** |
|  |  | (0.18) | (0.15) | (0.11) |  | (0.85) | (0.25) |
|  | Constant | -2.87*** | -2.93*** | -1.27*** |  | -11.20** | -0.34 |
|  |  | (0.77) | (0.60) | (0.42) |  | (5.09) | (0.78) |
| Hesitant | Average Rho | 0.41 | 0.44 | 0.02 |  | 1.27* | 0.60** |
|  |  | (0.29) | (0.28) | (0.18) |  | (0.68) | (0.25) |
|  | Risk in health | -0.02 | 0.00 | 0.02 |  | 0.11 | -0.09** |
|  |  | (0.04) | (0.04) | (0.02) |  | (0.11) | (0.04) |
|  | Female | 0.43* | -0.07 | -0.05 |  | -0.21 | -0.35* |
|  |  | (0.26) | (0.23) | (0.16) |  | (0.62) | (0.20) |
|  | Age | 0.05 | -0.10** | 0.02 |  | -0.03 | -0.10** |
|  |  | (0.06) | (0.04) | (0.03) |  | (0.11) | (0.04) |
|  | Age^2^ | <0.01* | <0.01 | <0.01 |  | <0.01 | <0.01* |
|  |  | (<0.01) | (<0.01) | (<0.01) |  | (<0.01) | (<0.01) |
|  | Bachelor | -0.52* | 0.01 | -1.08*** |  | 0.09 | -0.13 |
|  |  | (0.28) | (0.24) | (0.18) |  | (0.63) | (0.23) |
|  | High income | 0.30 | -0.99* | 0.17 |  | -14.63 | -0.16 |
|  |  | (0.31) | (0.53) | (0.27) |  | (1179.08) | (0.23) |
|  | With children | 0.73** | 0.09 | 0.08 |  | -0.54 | 0.35 |
|  |  | (0.31) | (0.25) | (0.17) |  | (0.67) | (0.26) |
|  | Constant | -4.41*** | -0.74 | -2.15*** |  | -4.72* | -0.54 |
|  |  | (1.24) | (0.81) | (0.61) |  | (2.70) | (0.85) |
| Vaccinated | (baseline) | - | - | - |  |  |  |
|  | Observations | 2,953 | 3,058 | 3,004 |  | 993 | 2,947 |
|  | Pseudo R2 | 0.05 | 0.07 | 0.07 |  | 0.24 | 0.04 |
|  | ll | -905.02 | -1,098.39 | -1,829.77 |  | -94.86 | -890.95 |
|  | Akaike's Crit | 103.63 | 172.99 | 286.89 |  | 60.79 | 81.02 |
|  | Bayesian Crit | 1,846.03 | 2,232.78 | 3,695.53 |  | 225.72 | 1,817.90 |
| Standard errors are in parentheses | | | | |  |  |  |
| *** p<.01, ** p<.05, * p<.1 | | | | |  |  |  |

**Table A14** Multinomial logistic regression on revealed preferences with constant discounting –Eastern European countries

|  |  | Croatia | Latvia | Lithuania | Slovakia | Slovenia |
| --- | --- | --- | --- | --- | --- | --- |
|  |  |  |  |  |  |  |
| Refuser | Average Rho | 0.50** | 0.17 | 0.07 | 0.11 | 0.27 |
|  |  | (0.19) | (0.20) | (0.22) | (0.21) | (0.19) |
|  | Risk in health | 0.03 | -0.02 | 0.05 | -0.04 | 0.04 |
|  |  | (0.03) | (0.03) | (0.03) | (0.03) | (0.03) |
|  | Female | -0.06 | 0.16 | -0.04 | 0.09 | 0.30* |
|  |  | (0.16) | (0.17) | (0.18) | (0.17) | (0.15) |
|  | Age | 0.04 | -0.02 | 0.05 | 0.00 | 0.02 |
|  |  | (0.03) | (0.03) | (0.03) | (0.03) | (0.03) |
|  | Age^2^ | 0.00** | 0.00 | 0.00* | 0.00 | 0.00 |
|  |  | (0.00) | (0.00) | (0.00) | (0.00) | (0.00) |
|  | Bachelor | -0.30* | -0.87*** | -0.41** | -0.65*** | -0.16 |
|  |  | (0.17) | (0.20) | (0.19) | (0.19) | (0.16) |
|  | High income | -0.01 | -0.44* | 0.12 | -0.34 | 0.03 |
|  |  | (0.21) | (0.24) | (0.32) | (0.23) | (0.19) |
|  | With children | -0.32 | 0.01 | 0.00 | 0.01 | 0.03 |
|  |  | (0.20) | (0.19) | (0.20) | (0.19) | (0.20) |
|  | Constant | -1.19* | -0.63 | -1.89** | -0.47 | -1.56** |
|  |  | (0.67) | (0.64) | (0.74) | (0.69) | (0.64) |
| Hesitant | Average Rho | 0.19 | 0.26 | 0.26 | -0.19 | 0.15 |
|  |  | (0.27) | (0.30) | (0.35) | (0.32) | (0.28) |
|  | Risk in health | 0.05 | -0.01 | -0.01 | 0.02 | 0.06 |
|  |  | (0.04) | (0.05) | (0.05) | (0.05) | (0.04) |
|  | Female | 0.20 | 0.42 | 0.14 | -0.14 | 0.17 |
|  |  | (0.22) | (0.26) | (0.29) | (0.25) | (0.23) |
|  | Age | 0.06 | 0.05 | 0.02 | -0.05 | 0.03 |
|  |  | (0.04) | (0.05) | (0.05) | (0.04) | (0.04) |
|  | Age^2^ | 0.00** | 0.00 | 0.00 | 0.00 | 0.00 |
|  |  | (0.00) | (0.00) | (0.00) | (0.00) | (0.00) |
|  | Bachelor | -0.04 | -0.71** | -0.38 | -0.25 | -0.33 |
|  |  | (0.22) | (0.30) | (0.30) | (0.27) | (0.24) |
|  | High income | -0.22 | -0.69* | -0.71 | -0.87** | 0.22 |
|  |  | (0.29) | (0.42) | (0.74) | (0.36) | (0.26) |
|  | With children | 0.12 | 0.05 | -0.02 | 0.27 | -0.11 |
|  |  | (0.26) | (0.29) | (0.33) | (0.29) | (0.28) |
|  | Constant | -2.54*** | -3.14*** | -2.52** | -0.35 | -2.56*** |
|  |  | (0.92) | (1.08) | (1.17) | (0.96) | (0.93) |
| Vaccinated | (baseline) | - | - | - |  |  |
|  | Observations | 1036 | 1067 | 986 | 966 | 1042 |
|  | Pseudo R2 | 0.05 | 0.03 | 0.03 | 0.03 | 0.02 |
|  | ll | -807.57 | -737.73 | -632.22 | -730.31 | -820.63 |
|  | Akaike's Crit | 87.93 | 50.34 | 35.91 | 47.77 | 30.75 |
|  | Bayesian Crit | 1651.15 | 1511.47 | 1300.43 | 1496.63 | 1677.25 |
| Standard errors are in parentheses | | | | |  |  |
| *** p<.01, ** p<.05, * p<.1 | | | | |  |  |

**Table A15** Multinomial logistic regression on revealed preferences with constant discounting – Western European countries

|  |  | France | Israel | Italy | Norway | Spain | Sweden |
| --- | --- | --- | --- | --- | --- | --- | --- |
|  |  |  |  |  |  |  |  |
| Refuser | Average Rho | -0.36** | -0.56* | 0.02 | 0.47 | 0.24 | -0.56** |
|  |  | (0.15) | (0.30) | (0.22) | (0.34) | (0.20) | (0.28) |
|  | Risk in health | 0.03 | -0.06 | 0.06* | -0.04 | 0.04 | -0.01 |
|  |  | (0.02) | (0.04) | (0.03) | (0.05) | (0.03) | (0.04) |
|  | Female | 0.29** | -0.34 | 0.32* | 0.08 | -0.52*** | -0.06 |
|  |  | (0.13) | (0.23) | (0.18) | (0.29) | (0.17) | (0.20) |
|  | Age | 0.07*** | 0.10** | 0.08** | 0.09 | 0.06 | 0.16*** |
|  |  | (0.02) | (0.05) | (0.04) | (0.06) | (0.03) | (0.04) |
|  | Age^2^ | 0.00*** | 0.00** | 0.00** | 0.00** | 0.00 | 0*** |
|  |  | (0.00) | (0.00) | (0.00) | (0.00) | (0.00) | (0) |
|  | Bachelor | -0.52*** | -0.67*** | -0.37* | -0.15 | -0.12 | 0.03 |
|  |  | (0.17) | (0.24) | (0.20) | (0.29) | (0.18) | (0.20) |
|  | High income | -0.33* | -0.69 | -0.62** | -0.27 | -0.26 | -0.64** |
|  |  | (0.20) | (0.48) | (0.31) | (0.76) | (0.40) | (0.32) |
|  | With children | -0.36*** | 0.01 | -0.08 | -0.11 | -0.46** | -0.24 |
|  |  | (0.14) | (0.27) | (0.20) | (0.32) | (0.18) | (0.21) |
|  | Constant | -2.80*** | -3.59*** | -4.87*** | -4.13*** | -3.82*** | -4.38*** |
|  |  | (0.54) | (0.97) | (0.94) | (1.23) | (0.78) | (0.97) |
| Hesitant | Average Rho | 0.01 | -0.01 | 0.67** | 1.65*** | 0.64** | 0.77** |
|  |  | (0.22) | (0.37) | (0.34) | (0.55) | (0.31) | (0.30) |
|  | Risk in health | 0.03 | -0.03 | 0.02 | 0.00 | 0.01 | 0.09* |
|  |  | (0.03) | (0.05) | (0.06) | (0.10) | (0.05) | (0.05) |
|  | Female | -0.03 | -0.42 | 0.82** | 0.28 | -0.30 | -0.32 |
|  |  | (0.20) | (0.31) | (0.36) | (0.52) | (0.28) | (0.27) |
|  | Age | -0.01 | 0.02 | -0.08 | 0.06 | 0.10 | 0.03 |
|  |  | (0.03) | (0.06) | (0.06) | (0.11) | (0.06) | (0.05) |
|  | Age^2^ | 0.00 | 0.00 | 0.00 | 0.00 | 0.00 | 0 |
|  |  | (0.00) | (0.00) | (0.00) | (0.00) | (0.00) | (0) |
|  | Bachelor | -0.35 | -0.44 | -0.41 | -0.41 | -0.40 | 0.62** |
|  |  | (0.26) | (0.33) | (0.37) | (0.53) | (0.31) | (0.27) |
|  | High income | -0.30 | -0.32 | -14.69 | -13.28 | -0.31 | -0.14 |
|  |  | (0.29) | (0.54) | (607.22) | (937.51) | (0.74) | (0.35) |
|  | With children | 0.32 | 0.26 | 0.37 | -0.32 | -0.26 | 0.13 |
|  |  | (0.23) | (0.36) | (0.40) | (0.55) | (0.32) | (0.28) |
|  | Constant | -2.69*** | -2.83** | -3.13** | -4.93** | -6.08*** | -4.18*** |
|  |  | (0.77) | (1.22) | (1.42) | (2.07) | (1.45) | (1.10) |
| Vaccinated | (baseline) | - | - | - | - | - | 1481 |
|  | Observations | 3116 | 1484 | 2992 | 1013 | 3240 | 0.06 |
|  | Pseudo R2 | 0.03 | 0.03 | 0.03 | 0.06 | 0.02 | -621.23 |
|  | ll | -1413.93 | -506.23 | -754.98 | -269.71 | -855.12 | 82.88 |
|  | Akaike's Crit | 76.45 | 36.61 | 52.46 | 33.47 | 32.44 | 1278.46 |
|  | Bayesian Crit | 2863.87 | 1048.45 | 1545.95 | 575.42 | 1746.24 | 1373.87 |
| Standard errors are in parentheses | | | | |  |  |  |
| *** p<.01, ** p<.05, * p<.1 | | | | |  |  |  |

**Table A16** Multinomial logistic regression on revealed preferences with constant discounting – Emerging countries

|  |  | Brazil | Chile | India | Russia | South Africa | Turkey |
| --- | --- | --- | --- | --- | --- | --- | --- |
|  |  |  |  |  |  |  |  |
| Refuser | Average Rho | 0.27 | -0.43 | 0.97* | 0.04 | -0.50*** | 0.18 |
|  |  | (0.32) | (0.36) | (0.51) | (0.12) | (0.13) | (0.16) |
|  | Risk in health | 0.08* | 0.07* | -0.03 | -0.06*** | -0.01 | -0.06*** |
|  |  | (0.04) | (0.04) | (0.06) | (0.02) | (0.02) | (0.02) |
|  | Female | -0.90*** | 0.17 | -0.13 | 0.01 | -0.34*** | 0.15 |
|  |  | (0.31) | (0.26) | (0.44) | (0.10) | (0.11) | (0.14) |
|  | Age | 0.11* | 0.05 | 0.02 | 0.06*** | 0.08*** | 0.03 |
|  |  | (0.06) | (0.06) | (0.09) | (0.02) | (0.02) | (0.03) |
|  | Age^2^ | 0.00 | 0.00 | 0.00 | 0.00*** | 0.00*** | 0.00 |
|  |  | (0.00) | (0.00) | (0.00) | (0.00) | (0.00) | (0.00) |
|  | Bachelor | 0.09 | -0.01 | -0.19 | -0.46*** | -0.52*** | -1.38*** |
|  |  | (0.31) | (0.29) | (0.51) | (0.10) | (0.11) | (0.15) |
|  | High income | -0.38 | -0.81*** | -0.72 | -0.38*** | -0.26** | -0.39** |
|  |  | (0.32) | (0.29) | (0.57) | (0.13) | (0.11) | (0.16) |
|  | With children | -0.48 | 1.01** | -0.86* | -0.53*** | -0.36*** | -1.53*** |
|  |  | (0.32) | (0.40) | (0.51) | (0.11) | (0.13) | (0.16) |
|  | Constant | -6.97*** | -5.52*** | -5.08*** | -1.15** | -2.16*** | -1.81*** |
|  |  | (1.47) | (1.19) | (1.71) | (0.47) | (0.48) | (0.53) |
| Hesitant | Average Rho | -0.14 | -0.05 | 0.61** | 0.19 | -0.28* | -0.78*** |
|  |  | (0.35) | (0.42) | (0.30) | (0.12) | (0.15) | (0.27) |
|  | Risk in health | 0.06 | -0.04 | 0.04 | 0.00 | -0.01 | -0.10*** |
|  |  | (0.04) | (0.05) | (0.04) | (0.02) | (0.02) | (0.03) |
|  | Female | -0.44 | -0.47 | 0.57** | -0.02 | -0.24** | -0.23 |
|  |  | (0.30) | (0.32) | (0.27) | (0.10) | (0.12) | (0.22) |
|  | Age | 0.05 | -0.08 | -0.02 | 0.03 | 0.08*** | -0.06 |
|  |  | (0.06) | (0.06) | (0.06) | (0.02) | (0.03) | (0.05) |
|  | Age^2^ | 0.00 | 0.00 | 0.00 | 0.00* | 0.00*** | 0.00 |
|  |  | (0.00) | (0.00) | (0.00) | (0.00) | (0.00) | (0.00) |
|  | Bachelor | -0.36 | -0.21 | 0.08 | -0.03 | -0.35*** | -0.81*** |
|  |  | (0.34) | (0.36) | (0.34) | (0.10) | (0.13) | (0.22) |
|  | High income | -0.46 | 0.28 | -0.63* | -0.37*** | -0.06 | -0.01 |
|  |  | (0.34) | (0.35) | (0.32) | (0.12) | (0.13) | (0.23) |
|  | With children | 0.19 | 0.60 | -0.66** | 0.01 | -0.16 | 0.01 |
|  |  | (0.35) | (0.43) | (0.31) | (0.13) | (0.15) | (0.28) |
|  | Constant | -5.03*** | -2.34* | -3.90*** | -1.55*** | -2.49*** | -0.31 |
|  |  | (1.30) | (1.24) | (1.06) | (0.48) | (0.56) | (0.89) |
| Vaccinated | (baseline) | - | - | - | - | - |  |
|  | Observations | 2983 | 2984 | 3097 | 2851 | 2872 | 3062 |
|  | Pseudo R2 | 0.04 | 0.03 | 0.04 | 0.02 | 0.03 | 0.14 |
|  | ll | -492.04 | -522.63 | -412.5 | -2791.26 | -2167.78 | -1105.99 |
|  | Akaike's Crit | 38.28 | 28.87 | 35.57 | 94.59 | 114.06 | 356.35 |
|  | Bayesian Crit | 1020.07 | 1081.26 | 860.99 | 5618.52 | 4371.55 | 2247.98 |
| Standard errors are in parentheses | | | | |  |  |  |
| *** p<.01, ** p<.05, * p<.1 | | | | |  |  |  |

- 1. **Stated preferences – constant discounting**

**Table A17** Multinomial logistic regression on stated preferences with constant discounting – Anglo-Saxon and East Asian countries

|  |  | Australia | UK | USA |  | Singapore | South Korea |
| --- | --- | --- | --- | --- | --- | --- | --- |
|  |  |  |  |  |  |  |  |
| Refuser | Average Rho | -0.36** | -0.49*** | -0.80*** |  | -0.22 | -0.36* |
|  |  | (0.17) | (0.17) | (0.13) |  | (0.27) | (0.18) |
|  | Risk in health | -0.06** | 0.01 | -0.02 |  | -0.16*** | -0.12*** |
|  |  | (0.02) | (0.02) | (0.02) |  | (0.04) | (0.02) |
|  | Female | 0.39*** | 0.16 | 0.33*** |  | 0.72*** | 0.27** |
|  |  | (0.13) | (0.12) | (0.11) |  | (0.23) | (0.12) |
|  | Age | 0.12*** | 0.12*** | 0.05** |  | 0.18*** | 0.06* |
|  |  | (0.03) | (0.03) | (0.02) |  | (0.06) | (0.03) |
|  | Age^2^ | 0.00*** | 0.00*** | 0.00*** |  | 0.00*** | 0.00** |
|  |  | (0.00) | (0.00) | (0.00) |  | (0.00) | (0.00) |
|  | Bachelor | -0.74*** | -0.45*** | -0.99*** |  | -0.35 | 0.24 |
|  |  | (0.15) | (0.14) | (0.12) |  | (0.23) | (0.15) |
|  | High income | -0.21 | -0.55** | -1.30*** |  | 0.83*** | -0.18 |
|  |  | (0.19) | (0.25) | (0.33) |  | (0.29) | (0.14) |
|  | With children | -0.02 | 0.00 | -0.11 |  | -0.80*** | -0.28* |
|  |  | (0.14) | (0.13) | (0.11) |  | (0.23) | (0.15) |
|  | Constant | -3.29*** | -3.19*** | -1.32*** |  | -5.47*** | -1.99*** |
|  |  | (0.65) | (0.55) | (0.43) |  | (1.36) | (0.64) |
| Hesitant | Average Rho | -0.02 | 0.24* | -0.18 |  | -0.18 | 0.26** |
|  |  | (0.12) | (0.12) | (0.12) |  | (0.20) | (0.11) |
|  | Risk in health | -0.03* | -0.02 | 0.00 |  | -0.05* | -0.06*** |
|  |  | (0.02) | (0.02) | (0.02) |  | (0.03) | (0.02) |
|  | Female | -0.01 | -0.14 | 0.09 |  | 0.05 | 0.06 |
|  |  | (0.10) | (0.10) | (0.10) |  | (0.16) | (0.08) |
|  | Age | 0.01 | -0.06*** | -0.02 |  | -0.03 | -0.07*** |
|  |  | (0.02) | (0.02) | (0.02) |  | (0.03) | (0.02) |
|  | Age^2^ | 0.00* | 0.00** | 0.00 |  | 0.00 | 0.00** |
|  |  | (0.00) | (0.00) | (0.00) |  | (0.00) | (0.00) |
|  | Bachelor | -0.16 | -0.11 | -0.39*** |  | -0.03 | 0.11 |
|  |  | (0.11) | (0.10) | (0.11) |  | (0.17) | (0.10) |
|  | High income | 0.02 | -0.06 | -0.32* |  | -0.36 | -0.06 |
|  |  | (0.14) | (0.16) | (0.17) |  | (0.26) | (0.09) |
|  | With children | -0.12 | 0.02 | -0.02 |  | 0.14 | 0.13 |
|  |  | (0.11) | (0.10) | (0.11) |  | (0.18) | (0.11) |
|  | Constant | -0.97** | 0.59* | -0.23 |  | 0.16 | 1.34*** |
|  |  | (0.45) | (0.35) | (0.38) |  | (0.76) | (0.40) |
| Vaccinated | (baseline) | - | - | - |  | - | - |
|  | Observations | 2953 | 3058 | 3004 |  | 993 | 2947 |
|  | Pseudo R2 | 0.03 | 0.04 | 0.05 |  | 0.06 | 0.03 |
|  | ll | -2234.09 | -2384.92 | -2534.33 |  | -780.18 | -2630.97 |
|  | Akaike's Crit | 146.94 | 212.2 | 276.39 |  | 97.6 | 174.42 |
|  | Bayesian Crit | 4504.17 | 4805.84 | 5104.66 |  | 1596.36 | 5297.94 |
| Standard errors are in parentheses | | | | |  |  |  |
| *** p<.01, ** p<.05, * p<.1 | | | | |  |  |  |

**Table A18** Multinomial logistic regression on stated preferences with constant discounting –Eastern European countries

|  |  | Croatia | Latvia | Lithuania | Slovakia | Slovenia |
| --- | --- | --- | --- | --- | --- | --- |
|  |  |  |  |  |  |  |
| Refuser | Average Rho | 0.00 | 0.04 | 0.08 | -0.19 | 0.15 |
|  |  | (0.19) | (0.18) | (0.19) | (0.20) | (0.18) |
|  | Risk in health | 0.07** | -0.04 | 0.02 | -0.02 | 0.00 |
|  |  | (0.03) | (0.03) | (0.03) | (0.03) | (0.02) |
|  | Female | 0.42*** | 0.64*** | 0.41*** | 0.20 | 0.46*** |
|  |  | (0.15) | (0.15) | (0.15) | (0.16) | (0.14) |
|  | Age | 0.06** | 0.02 | 0.08*** | 0.09*** | 0.06** |
|  |  | (0.03) | (0.03) | (0.03) | (0.03) | (0.03) |
|  | Age^2^ | 0.00*** | 0.00 | 0.00*** | 0.00*** | 0.00** |
|  |  | (0.00) | (0.00) | (0.00) | (0.00) | (0.00) |
|  | Bachelor | -0.27* | -0.54*** | -0.34** | -0.22 | -0.06 |
|  |  | (0.15) | (0.16) | (0.16) | (0.17) | (0.15) |
|  | High income | -0.03 | -0.39** | 0.02 | -0.28 | -0.06 |
|  |  | (0.20) | (0.20) | (0.29) | (0.21) | (0.18) |
|  | With children | 0.02 | -0.05 | 0.16 | 0.12 | -0.18 |
|  |  | (0.19) | (0.17) | (0.17) | (0.18) | (0.19) |
|  | Constant | -1.12* | 0.39 | -1.46** | -2.26*** | -1.44** |
|  |  | (0.66) | (0.62) | (0.64) | (0.70) | (0.62) |
| Hesitant | Average Rho | 0.18 | 0.43** | 0.18 | 0.20 | 0.27 |
|  |  | (0.21) | (0.22) | (0.23) | (0.23) | (0.22) |
|  | Risk in health | 0.01 | 0.06** | 0.01 | -0.03 | -0.02 |
|  |  | (0.03) | (0.03) | (0.03) | (0.03) | (0.03) |
|  | Female | -0.02 | -0.03 | 0.07 | -0.35* | 0.23 |
|  |  | (0.17) | (0.18) | (0.18) | (0.19) | (0.17) |
|  | Age | 0.00 | -0.05 | -0.03 | -0.03 | 0.05 |
|  |  | (0.03) | (0.03) | (0.03) | (0.03) | (0.03) |
|  | Age^2^ | 0.00 | 0.00 | 0.00 | 0.00 | 0.00** |
|  |  | (0.00) | (0.00) | (0.00) | (0.00) | (0.00) |
|  | Bachelor | -0.08 | 0.21 | 0.29 | 0.20 | -0.01 |
|  |  | (0.17) | (0.19) | (0.21) | (0.19) | (0.18) |
|  | High income | -0.09 | 0.12 | -0.15 | -0.64*** | 0.21 |
|  |  | (0.22) | (0.22) | (0.35) | (0.24) | (0.20) |
|  | With children | 0.18 | -0.26 | -0.07 | 0.24 | -0.37* |
|  |  | (0.21) | (0.21) | (0.20) | (0.21) | (0.21) |
|  | Constant | 0.06 | 0.81 | -0.03 | 0.49 | -1.22* |
|  |  | (0.70) | (0.68) | (0.69) | (0.70) | (0.70) |
| Vaccinated | (baseline) | - | - | - | - | - |
|  | Observations | 1036 | 1067 | 986 | 966 | 1042 |
|  | Pseudo R2 | 0.04 | 0.05 | 0.02 | 0.03 | 0.02 |
|  | ll | -1055.77 | -1064.97 | -1020.09 | -944.9 | -1063.21 |
|  | Akaike's Crit | 90.37 | 104.97 | 47.94 | 63.46 | 50.8 |
|  | Bayesian Crit | 2147.54 | 2165.93 | 2076.18 | 1925.81 | 2162.43 |
| Standard errors are in parentheses | | | | |  |  |
| *** p<.01, ** p<.05, * p<.1 | | | | |  |  |

**Table A19** Multinomial logistic regression on stated preferences with constant discounting – Western European countries

|  |  | France | Israel | Italy | Norway | Spain | Sweden |
| --- | --- | --- | --- | --- | --- | --- | --- |
|  |  |  |  |  |  |  |  |
| Refuser | Average Rho | -0.75*** | 0.58*** | -0.01 | 0.13 | -0.29* | -0.56** |
|  |  | (0.12) | (0.21) | (0.13) | (0.30) | (0.15) | (0.28) |
|  | Risk in health | 0.00 | -0.04 | -0.01 | -0.08* | 0.02 | -0.01 |
|  |  | (0.02) | (0.03) | (0.02) | (0.05) | (0.02) | (0.04) |
|  | Female | 0.52*** | 0.07 | 0.33*** | 0.50** | 0.04 | -0.06 |
|  |  | (0.10) | (0.16) | (0.11) | (0.24) | (0.11) | (0.20) |
|  | Age | 0.05*** | -0.02 | 0.12*** | 0.17*** | 0.05** | 0.16*** |
|  |  | (0.02) | (0.03) | (0.03) | (0.05) | (0.02) | (0.04) |
|  | Age^2^ | 0.00*** | 0.00 | 0.00*** | 0.00*** | 0.00** | 0*** |
|  |  | (0.00) | (0.00) | (0.00) | (0.00) | (0.00) | (0) |
|  | Bachelor | -0.30** | 0.54*** | -0.26** | -0.05 | -0.13 | 0.03 |
|  |  | (0.12) | (0.16) | (0.12) | (0.24) | (0.12) | (0.20) |
|  | High income | -0.57*** | 1.26*** | -0.47*** | -0.05 | -0.18 | -0.64** |
|  |  | (0.15) | (0.35) | (0.17) | (0.64) | (0.26) | (0.32) |
|  | With children | -0.20* | -0.31 | 0.16 | -0.35 | -0.29** | -0.24 |
|  |  | (0.11) | (0.19) | (0.13) | (0.26) | (0.13) | (0.21) |
|  | Constant | -1.31*** | 0.44 | -4.12*** | -5.14*** | -2.46*** | -4.38*** |
|  |  | (0.43) | (0.66) | (0.62) | (1.14) | (0.54) | (0.97) |
| Hesitant | Average Rho | -0.05 | 0.84*** | -0.22* | 0.12 | 0.04 | 0.77** |
|  |  | (0.10) | (0.20) | (0.12) | (0.20) | (0.12) | (0.30) |
|  | Risk in health | -0.01 | 0.03 | 0.00 | -0.02 | -0.01 | 0.09* |
|  |  | (0.02) | (0.03) | (0.02) | (0.03) | (0.02) | (0.05) |
|  | Female | 0.04 | -0.11 | 0.05 | -0.34** | -0.17* | -0.32 |
|  |  | (0.09) | (0.16) | (0.09) | (0.16) | (0.10) | (0.27) |
|  | Age | -0.03* | -0.07** | 0.01 | 0.01 | -0.02 | 0.03 |
|  |  | (0.02) | (0.03) | (0.02) | (0.03) | (0.02) | (0.05) |
|  | Age^2^ | 0.00 | 0.00** | 0.00 | 0.00 | 0.00 | 0 |
|  |  | (0.00) | (0.00) | (0.00) | (0.00) | (0.00) | (0) |
|  | Bachelor | 0.03 | 0.43*** | 0.15 | 0.10 | 0.13 | 0.62** |
|  |  | (0.11) | (0.16) | (0.10) | (0.16) | (0.10) | (0.27) |
|  | High income | 0.01 | 1.15*** | -0.20 | 0.55* | 0.16 | -0.14 |
|  |  | (0.12) | (0.35) | (0.13) | (0.32) | (0.19) | (0.35) |
|  | With children | -0.06 | -0.09 | 0.14 | -0.12 | -0.17 | 0.13 |
|  |  | (0.10) | (0.19) | (0.11) | (0.18) | (0.11) | (0.28) |
|  | Constant | 0.48 | 1.78*** | -0.99** | -0.68 | -0.42 | -4.18*** |
|  |  | (0.35) | (0.63) | (0.45) | (0.59) | (0.41) | (1.10) |
| Vaccinated | (baseline) | - | - | - | - | - | 1481 |
|  | Observations | 3116 | 1484 | 2992 | 1013 | 3240 | 0.06 |
|  | Pseudo R2 | 0.03 | 0.05 | 0.02 | 0.03 | 0.01 | -621.23 |
|  | ll | -3046.46 | -1444.15 | -2602.32 | -794.46 | -2533.78 | 82.88 |
|  | Akaike's Crit | 194.22 | 139.25 | 104.73 | 47.39 | 72.03 | 1278.46 |
|  | Bayesian Crit | 6128.92 | 2924.3 | 5240.64 | 1624.92 | 5103.56 | 1373.87 |
| Standard errors are in parentheses | | | | |  |  |  |
| *** p<.01, ** p<.05, * p<.1 | | | | |  |  |  |

**Table A20** Multinomial logistic regression on stated preferences with constant discounting – Emerging countries

|  |  | Brazil | Chile | India | Russia | South Africa | Turkey |
| --- | --- | --- | --- | --- | --- | --- | --- |
|  |  |  |  |  |  |  |  |
| Refuser | Average Rho | -0.10 | -0.15 | -0.47* | -0.16 | -0.42*** | 0.04 |
|  |  | (0.20) | (0.16) | (0.28) | (0.11) | (0.12) | (0.12) |
|  | Risk in health | 0.06** | 0.01 | -0.07** | -0.06*** | -0.02 | -0.10*** |
|  |  | (0.03) | (0.02) | (0.03) | (0.02) | (0.01) | (0.02) |
|  | Female | -0.49*** | 0.01 | 0.10 | 0.23** | -0.07 | -0.08 |
|  |  | (0.17) | (0.12) | (0.25) | (0.09) | (0.09) | (0.10) |
|  | Age | 0.08** | 0.06** | 0.04 | 0.10*** | 0.11*** | 0.03 |
|  |  | (0.04) | (0.03) | (0.05) | (0.02) | (0.02) | (0.02) |
|  | Age^2^ | 0.00 | 0.00*** | 0.00 | 0.00*** | 0.00*** | 0.00 |
|  |  | (0.00) | (0.00) | (0.00) | (0.00) | (0.00) | (0.00) |
|  | Bachelor | -0.01 | 0.02 | -0.04 | -0.21** | -0.32*** | -0.38*** |
|  |  | (0.18) | (0.14) | (0.32) | (0.09) | (0.10) | (0.11) |
|  | High income | -0.21 | -0.27** | -0.27 | -0.62*** | 0.07 | 0.08 |
|  |  | (0.19) | (0.13) | (0.28) | (0.12) | (0.10) | (0.11) |
|  | With children | -0.35* | 0.23 | -0.95*** | -0.39*** | -0.40*** | -0.96*** |
|  |  | (0.19) | (0.15) | (0.29) | (0.11) | (0.11) | (0.12) |
|  | Constant | -4.64*** | -2.70*** | -3.85*** | -1.71*** | -2.70*** | -1.06** |
|  |  | (0.80) | (0.55) | (1.05) | (0.46) | (0.44) | (0.44) |
| Hesitant | Average Rho | 0.25* | 0.22 | 0.16 | 0.10 | 0.11 | -0.02 |
|  |  | (0.15) | (0.15) | (0.11) | (0.12) | (0.12) | (0.11) |
|  | Risk in health | -0.01 | 0.01 | -0.03** | -0.01 | 0.01 | -0.06*** |
|  |  | (0.02) | (0.02) | (0.01) | (0.02) | (0.02) | (0.01) |
|  | Female | -0.17 | -0.20 | 0.07 | 0.06 | -0.09 | -0.23** |
|  |  | (0.13) | (0.12) | (0.10) | (0.10) | (0.10) | (0.09) |
|  | Age | -0.01 | -0.01 | -0.02 | 0.03 | -0.04** | -0.05** |
|  |  | (0.03) | (0.03) | (0.02) | (0.02) | (0.02) | (0.02) |
|  | Age^2^ | 0.00 | 0.00 | 0.00 | 0.00* | 0.00 | 0.00* |
|  |  | (0.00) | (0.00) | (0.00) | (0.00) | (0.00) | (0.00) |
|  | Bachelor | -0.16 | -0.04 | -0.06 | 0.02 | 0.23** | 0.14 |
|  |  | (0.15) | (0.14) | (0.13) | (0.11) | (0.11) | (0.10) |
|  | High income | -0.23 | -0.13 | -0.11 | -0.43*** | -0.17 | -0.05 |
|  |  | (0.15) | (0.13) | (0.11) | (0.12) | (0.11) | (0.10) |
|  | With children | -0.14 | 0.03 | -0.20* | -0.35*** | -0.26** | -0.33*** |
|  |  | (0.15) | (0.14) | (0.12) | (0.12) | (0.12) | (0.12) |
|  | Constant | -1.62*** | -0.79 | -1.06*** | -0.54 | 0.16 | 0.78* |
|  |  | (0.54) | (0.51) | (0.40) | (0.46) | (0.43) | (0.40) |
| Vaccinated | (baseline) | - | - | - | - | - |  |
|  | Observations | 2983 | 2984 | 3097 | 2851 | 2872 | 3062 |
|  | Pseudo R2 | 0.02 | 0.03 | 0.01 | 0.02 | 0.03 | 0.04 |
|  | ll | -1485.5 | -2055.3 | -1683.6 | -2983.05 | -2677.84 | -2733.14 |
|  | Akaike's Crit | 55.05 | 121.97 | 46.29 | 131.98 | 142.29 | 249.25 |
|  | Bayesian Crit | 3007.01 | 4146.61 | 3403.2 | 6002.09 | 5391.68 | 5502.29 |
| Standard errors are in parentheses | | | | |  |  |  |
| *** p<.01, ** p<.05, * p<.1 | | | | |  |  |  |

- 1. **Revealed preferences – quasi-hyperbolic discounting**

***Table A21*** *Multinomial logistic regression with quasi-hyperbolic discounting – Anglo-Saxon and East Asian countries*

|  |  | Australia | UK | USA |  | Singapore | South Korea |
| --- | --- | --- | --- | --- | --- | --- | --- |
|  |  |  |  |  |  |  |  |
| Refuser | Rho2 | -0.15 | -0.37* | -0.47*** |  | -0.37 | 0.05 |
|  |  | (0.21) | (0.19) | (0.13) |  | (1.03) | (0.27) |
|  | Beta | -0.03 | 0.01 | 0.03*** |  | -0.68 | 0.03 |
|  |  | (0.03) | (0.02) | (0.01) |  | (0.90) | (0.02) |
|  | Risk in health | -0.05* | 0.03 | -0.01 |  | -0.48*** | -0.06 |
|  |  | (0.03) | (0.03) | (0.02) |  | (0.18) | (0.04) |
|  | Female | 0.37** | -0.06 | 0.33*** |  | -0.44 | -0.19 |
|  |  | (0.17) | (0.14) | (0.11) |  | (0.69) | (0.19) |
|  | Age | 0.04 | 0.09*** | 0.04* |  | 0.45** | -0.08** |
|  |  | (0.03) | (0.03) | (0.02) |  | (0.23) | (0.04) |
|  | Age^2^ | 0.00** | 0.00*** | 0.00*** |  | -0.01** | 0.00* |
|  |  | (0.00) | (0.00) | (0.00) |  | (0.00) | (0.00) |
|  | Bachelor | -0.91*** | -0.50*** | -1.18*** |  | -1.88** | -0.37* |
|  |  | (0.20) | (0.16) | (0.13) |  | (0.87) | (0.21) |
|  | High income | -0.56* | -0.40 | -1.33*** |  | 2.45*** | -0.89*** |
|  |  | (0.29) | (0.27) | (0.36) |  | (0.84) | (0.28) |
|  | With children | 0.06 | -0.06 | -0.16 |  | -2.18** | -0.79*** |
|  |  | (0.18) | (0.15) | (0.11) |  | (0.87) | (0.25) |
|  | Constant | -2.73*** | -2.90*** | -1.32*** |  | -10.49** | -0.43 |
|  |  | (0.78) | (0.60) | (0.42) |  | (5.23) | (0.78) |
| Hesitant | Rho2 | 0.43 | 0.66** | -0.01 |  | 1.19* | 0.65*** |
|  |  | (0.29) | (0.27) | (0.18) |  | (0.65) | (0.25) |
|  | Beta | 0.00 | -0.12* | -0.05 |  | -0.20 | -0.01 |
|  |  | (0.03) | (0.07) | (0.03) |  | (0.24) | (0.03) |
|  | Risk in health | -0.02 | 0 | 0.02 |  | 0.13 | -0.09** |
|  |  | (0.04) | (0.04) | (0.03) |  | (0.11) | (0.04) |
|  | Female | 0.43 | -0.05 | -0.06 |  | -0.20 | -0.35* |
|  |  | (0.26) | (0.23) | (0.16) |  | (0.62) | (0.20) |
|  | Age | 0.06 | -0.10** | 0.02 |  | -0.03 | -0.10** |
|  |  | (0.06) | (0.04) | (0.03) |  | (0.11) | (0.04) |
|  | Age^2^ | 0.00* | 0 | 0 |  | 0 | 0* |
|  |  | (0.00) | (0) | (0) |  | (0) | (0) |
|  | Bachelor | -0.51* | 0.01 | -1.09*** |  | 0.10 | -0.13 |
|  |  | (0.28) | (0.24) | (0.18) |  | (0.63) | (0.23) |
|  | High income | 0.30 | -0.97* | 0.19 |  | -15.48 | -0.16 |
|  |  | (0.31) | (0.53) | (0.27) |  | (1729.05) | (0.23) |
|  | With children | 0.74** | 0.06 | 0.06 |  | -0.59 | 0.35 |
|  |  | (0.31) | (0.25) | (0.17) |  | (0.68) | (0.26) |
|  | Constant | -4.47*** | -0.57 | -2.03*** |  | -4.32 | -0.52 |
|  |  | (1.24) | (0.81) | (0.61) |  | (2.68) | (0.85) |
| Vaccinated | (baseline) | - | - | - |  | - | - |
|  | Observations | 2,953 | 3,058 | 3,004 |  | 993 | 2,947 |
|  | Pseudo R2 | 0.06 | 0.08 | 0.08 |  | 0.25 | 0.05 |
|  | ll | -903.50 | -1093.32 | -1824.13 |  | -94.19 | -889.36 |
|  | Akaike's Crit | 1,847.01 | 2226.65 | 3688.26 |  | 228.39 | 1818.71 |
|  | Bayesian Crit | 1,966.82 | 2347.16 | 3808.42 |  | 326.4 | 1938.48 |
| Standard errors are in parentheses | | | | |  |  |  |
| *** p<.01, ** p<.05, * p<.1 | | | | |  |  |  |

**Table A22** Multinomial logistic regression on revealed preferences with quasi-hyperbolic discounting – Eastern European countries

|  |  | Croatia | Latvia | Lithuania | Slovakia | Slovenia |
| --- | --- | --- | --- | --- | --- | --- |
|  |  |  |  |  |  |  |
| Refuser | Rho2 | 0.46** | 0.24 | 0.14 | 0.10 | 0.37* |
|  |  | (0.20) | (0.19) | (0.22) | (0.22) | (0.20) |
|  | Beta | 0.02 | -0.02 | -0.03 | 0.01 | -0.04* |
|  |  | (0.01) | (0.02) | (0.02) | (0.02) | (0.02) |
|  | Risk in health | 0.03 | -0.02 | 0.05 | -0.04 | 0.04 |
|  |  | (0.03) | (0.03) | (0.03) | (0.03) | (0.03) |
|  | Female | -0.06 | 0.16 | -0.04 | 0.09 | 0.30* |
|  |  | (0.16) | (0.17) | (0.18) | (0.17) | (0.16) |
|  | Age | 0.04 | -0.02 | 0.05 | 0.01 | 0.02 |
|  |  | (0.03) | (0.03) | (0.03) | (0.03) | (0.03) |
|  | Age^2^ | 0.00** | 0.00 | 0.00** | 0.00 | 0.00 |
|  |  | (0.00) | (0.00) | (0.00) | (0.00) | (0.00) |
|  | Bachelor | -0.29* | -0.88*** | -0.45** | -0.66*** | -0.16 |
|  |  | (0.17) | (0.20) | (0.19) | (0.19) | (0.16) |
|  | High income | 0.00 | -0.44* | 0.13 | -0.34 | 0.03 |
|  |  | (0.22) | (0.24) | (0.32) | (0.23) | (0.19) |
|  | With children | -0.34* | 0.02 | -0.02 | 0.00 | 0.02 |
|  |  | (0.20) | (0.19) | (0.20) | (0.20) | (0.20) |
|  | Constant | -1.24* | -0.52 | -1.84** | -0.51 | -1.50** |
|  |  | (0.67) | (0.65) | (0.74) | (0.69) | (0.64) |
| Hesitant | Rho2 | 0.24 | 0.19 | 0.41 | 0.04 | 0.18 |
|  |  | (0.27) | (0.30) | (0.34) | (0.32) | (0.29) |
|  | Beta | 0.00 | -0.03 | -0.06 | -0.01 | 0 |
|  |  | (0.02) | (0.03) | (0.04) | (0.02) | (0.02) |
|  | Risk in health | 0.04 | 0.00 | -0.01 | 0.02 | 0.05 |
|  |  | (0.04) | (0.05) | (0.05) | (0.05) | (0.04) |
|  | Female | 0.19 | 0.42 | 0.14 | -0.14 | 0.17 |
|  |  | (0.22) | (0.26) | (0.29) | (0.25) | (0.23) |
|  | Age | 0.06 | 0.05 | 0.02 | -0.05 | 0.03 |
|  |  | (0.04) | (0.05) | (0.05) | (0.04) | (0.04) |
|  | Age^2^ | 0.00** | 0.00 | 0.00 | 0 | 0 |
|  |  | (0.00) | (0.00) | (0.00) | (0) | (0) |
|  | Bachelor | -0.04 | -0.73** | -0.42 | -0.24 | -0.33 |
|  |  | (0.22) | (0.30) | (0.30) | (0.27) | (0.24) |
|  | High income | -0.22 | -0.70* | -0.70 | -0.86** | 0.22 |
|  |  | (0.29) | (0.42) | (0.74) | (0.36) | (0.26) |
|  | With children | 0.12 | 0.06 | -0.05 | 0.27 | -0.12 |
|  |  | (0.26) | (0.29) | (0.33) | (0.29) | (0.28) |
|  | Constant | -2.57*** | -2.95*** | -2.43** | -0.42 | -2.57*** |
|  |  | (0.92) | (1.09) | (1.18) | (0.95) | (0.93) |
| Vaccinated | (baseline) | - | - | - |  |  |
|  | Observations | 1,036 | 1,067 | 986 | 966 | 1042 |
|  | Pseudo R2 | 0.05 | 0.03 | 0.03 | 0.03 | 0.02 |
|  | ll | -804.89 | -736.67 | -629.6 | -730.31 | -818.75 |
|  | Akaike's Crit | 1,649.79 | 1,513.35 | 1299.2 | 1500.62 | 1677.5 |
|  | Bayesian Crit | 1,748.65 | 1,612.80 | 1397.08 | 1598.09 | 1776.48 |
| Standard errors are in parentheses | | | | |  |  |
| *** p<.01, ** p<.05, * p<.1 | | | | |  |  |

**Table A23** Multinomial logistic regression on revealed preferences with quasi-hyperbolic discounting – Western European countries

|  |  | France | Israel | Italy | Norway | Spain | Sweden |
| --- | --- | --- | --- | --- | --- | --- | --- |
|  |  |  |  |  |  |  |  |
| Refuser | Rho2 | -0.34** | 0.20 | 0.20 | 0.20 | 0.20 | -0.55* |
|  |  | (0.16) | (0.21) | (0.21) | (0.21) | (0.21) | (0.29) |
|  | Beta | 0.02 | 0.02* | 0.02* | 0.02* | 0.02* | -0.02 |
|  |  | (0.01) | (0.01) | (0.01) | (0.01) | (0.01) | (0.05) |
|  | Risk in health | 0.03 | 0.04 | 0.04 | 0.04 | 0.04 | -0.01 |
|  |  | (0.02) | (0.03) | (0.03) | (0.03) | (0.03) | (0.04) |
|  | Female | 0.29** | -0.53*** | -0.53*** | -0.53*** | -0.53*** | -0.07 |
|  |  | (0.13) | (0.17) | (0.17) | (0.17) | (0.17) | (0.20) |
|  | Age | 0.07*** | 0.05 | 0.05 | 0.05 | 0.05 | 0.16*** |
|  |  | (0.02) | (0.03) | (0.03) | (0.03) | (0.03) | (0.04) |
|  | Age^2^ | 0.00*** | 0.00 | 0.00 | 0.00 | 0.00 | 0.00*** |
|  |  | (0.00) | (0.00) | (0.00) | (0.00) | (0.00) | (0.00) |
|  | Bachelor | -0.52*** | -0.12 | -0.12 | -0.12 | -0.12 | 0.02 |
|  |  | (0.17) | (0.18) | (0.18) | (0.18) | (0.18) | (0.20) |
|  | High income | -0.32* | -0.25 | -0.25 | -0.25 | -0.25 | -0.63** |
|  |  | (0.20) | (0.40) | (0.40) | (0.40) | (0.40) | (0.32) |
|  | With children | -0.36*** | -0.45** | -0.45** | -0.45** | -0.45** | -0.24 |
|  |  | (0.14) | (0.19) | (0.19) | (0.19) | (0.19) | (0.21) |
|  | Constant | -2.87*** | -3.85*** | -3.85*** | -3.85*** | -3.85*** | -4.36*** |
|  |  | (0.54) | (0.78) | (0.78) | (0.78) | (0.78) | (0.96) |
| Hesitant | Rho2 | -0.03 | 0.43 | 0.43 | 0.43 | 0.43 | 0.67** |
|  |  | (0.23) | (0.33) | (0.33) | (0.33) | (0.33) | (0.31) |
|  | Beta | 0.00 | 0.02 | 0.02 | 0.02 | 0.02 | 0.01 |
|  |  | (0.02) | (0.02) | (0.02) | (0.02) | (0.02) | (0.03) |
|  | Risk in health | 0.03 | 0.01 | 0.01 | 0.01 | 0.01 | 0.09* |
|  |  | (0.03) | (0.05) | (0.05) | (0.05) | (0.05) | (0.05) |
|  | Female | -0.03 | -0.31 | -0.31 | -0.31 | -0.31 | -0.32 |
|  |  | (0.20) | (0.29) | (0.29) | (0.29) | (0.29) | (0.27) |
|  | Age | -0.01 | 0.09 | 0.09 | 0.09 | 0.09 | 0.03 |
|  |  | (0.03) | (0.06) | (0.06) | (0.06) | (0.06) | (0.05) |
|  | Age^2^ | 0.00 | 0 | 0 | 0 | 0 | 0 |
|  |  | (0.00) | (0) | (0) | (0) | (0) | (0) |
|  | Bachelor | -0.35 | -0.41 | -0.41 | -0.41 | -0.41 | 0.63** |
|  |  | (0.26) | (0.31) | (0.31) | (0.31) | (0.31) | (0.27) |
|  | High income | -0.30 | -0.30 | -0.30 | -0.30 | -0.30 | -0.20 |
|  |  | (0.29) | (0.74) | (0.74) | (0.74) | (0.74) | (0.35) |
|  | With children | 0.32 | -0.25 | -0.25 | -0.25 | -0.25 | 0.15 |
|  |  | (0.23) | (0.32) | (0.32) | (0.32) | (0.32) | (0.29) |
|  | Constant | -2.66*** | -6.01*** | -6.01*** | -6.01*** | -6.01*** | -4.12*** |
|  | Rho2 | (0.77) | (1.44) | (1.44) | (1.44) | (1.44) | (1.09) |
| Vaccinated | (baseline) | - | - | - | - | - | - |
|  | Observations | 3,116 | 3240 | 3240 | 3240 | 3240 | 1481 |
|  | Pseudo R2 | 0.03 | 0.02 | 0.02 | 0.02 | 0.02 | 0.06 |
|  | ll | -1,414.21 | -852.15 | -852.15 | -852.15 | -852.15 | -620.51 |
|  | Akaike's Crit | 2,868.41 | 1744.3 | 1744.3 | 1744.3 | 1744.3 | 1281.03 |
|  | Bayesian Crit | 2,989.30 | 1865.97 | 1865.97 | 1865.97 | 1865.97 | 1387.04 |
| Standard errors are in parentheses | | | | |  |  |  |
| *** p<.01, ** p<.05, * p<.1 | | | | |  |  |  |

**Table A24** Multinomial logistic regression on revealed preferences with quasi-hyperbolic discounting – Emerging countries

|  |  | Brazil | Chile | India | Russia | South Africa | Turkey |
| --- | --- | --- | --- | --- | --- | --- | --- |
|  |  |  |  |  |  |  |  |
| Refuser | Average Rho | 0.19 | -0.46 | 0.17 | 0.17 | 0.17 | 0.17 |
|  |  | (0.33) | (0.38) | (0.16) | (0.16) | (0.16) | (0.16) |
|  |  | -0.04 | -0.01 | -0.06** | -0.06** | -0.06** | -0.06** |
|  |  | (0.04) | (0.03) | (0.03) | (0.03) | (0.03) | (0.03) |
|  | Risk in health | 0.08* | 0.07* | -0.06** | -0.06** | -0.06** | -0.06** |
|  |  | (0.04) | (0.04) | (0.02) | (0.02) | (0.02) | (0.02) |
|  | Female | -0.88*** | 0.17 | 0.16 | 0.16 | 0.16 | 0.16 |
|  |  | (0.31) | (0.26) | (0.14) | (0.14) | (0.14) | (0.14) |
|  | Age | 0.11* | 0.05 | 0.03 | 0.03 | 0.03 | 0.03 |
|  |  | (0.06) | (0.06) | (0.03) | (0.03) | (0.03) | (0.03) |
|  | Age^2^ | 0.00 | 0.00 | 0.00 | 0.00 | 0.00 | 0.00 |
|  |  | (0.00) | (0.00) | (0.00) | (0.00) | (0.00) | (0.00) |
|  | Bachelor | 0.07 | -0.02 | -1.39*** | -1.39*** | -1.39*** | -1.39*** |
|  |  | (0.31) | (0.29) | (0.15) | (0.15) | (0.15) | (0.15) |
|  | High income | -0.38 | -0.82*** | -0.40** | -0.40** | -0.40** | -0.40** |
|  |  | (0.32) | (0.29) | (0.16) | (0.16) | (0.16) | (0.16) |
|  | With children | -0.48 | 1.02** | -1.52*** | -1.52*** | -1.52*** | -1.52*** |
|  |  | (0.32) | (0.40) | (0.16) | (0.16) | (0.16) | (0.16) |
|  | Constant | -6.80*** | -5.49*** | -1.72*** | -1.72*** | -1.72*** | -1.72*** |
|  |  | (1.47) | (1.19) | (0.53) | (0.53) | (0.53) | (0.53) |
| Hesitant | Average Rho | -0.16 | 0.13 | -0.66** | -0.66** | -0.66** | -0.66** |
|  |  | (0.35) | (0.40) | (0.27) | (0.27) | (0.27) | (0.27) |
|  |  | 0.03* | -0.04 | 0.03 | 0.03 | 0.03 | 0.03 |
|  |  | (0.02) | (0.04) | (0.02) | (0.02) | (0.02) | (0.02) |
|  | Risk in health | 0.06 | -0.04 | -0.10*** | -0.10*** | -0.10*** | -0.10*** |
|  |  | (0.04) | (0.05) | (0.03) | (0.03) | (0.03) | (0.03) |
|  | Female | -0.45 | -0.47 | -0.25 | -0.25 | -0.25 | -0.25 |
|  |  | (0.30) | (0.32) | (0.22) | (0.22) | (0.22) | (0.22) |
|  | Age | 0.05 | -0.08 | -0.07 | -0.07 | -0.07 | -0.07 |
|  |  | (0.06) | (0.06) | (0.05) | (0.05) | (0.05) | (0.05) |
|  | Age^2^ | 0.00 | 0.00 | 0 | 0 | 0 | 0 |
|  |  | (0.00) | (0.00) | (0) | (0) | (0) | (0) |
|  | Bachelor | -0.34 | -0.21 | -0.80*** | -0.80*** | -0.80*** | -0.80*** |
|  |  | (0.34) | (0.36) | (0.22) | (0.22) | (0.22) | (0.22) |
|  | High income | -0.45 | 0.27 | 0 | 0 | 0 | 0 |
|  |  | (0.34) | (0.35) | (0.23) | (0.23) | (0.23) | (0.23) |
|  | With children | 0.20 | 0.61 | 0 | 0 | 0 | 0 |
|  |  | (0.35) | (0.43) | (0.28) | (0.28) | (0.28) | (0.28) |
|  | Constant | -5.15*** | -2.35* | -0.38 | -0.38 | -0.38 | -0.38 |
|  |  | (1.31) | (1.24) | (0.88) | (0.88) | (0.88) | (0.88) |
| Vaccinated | (baseline) | - | - | - | - | - | - |
|  | Observations | 2,983 | 2,984 | 3062 | 3062 | 3062 | 3062 |
|  | Pseudo R2 | 0.04 | 0.03 | 0.14 | 0.14 | 0.14 | 0.14 |
|  | ll | -490.52 | -520.93 | -1103.36 | -1103.36 | -1103.36 | -1103.36 |
|  | Akaike's Crit | 1,021.05 | 1,081.87 | 2246.73 | 2246.73 | 2246.73 | 2246.73 |
|  | Bayesian Crit | 1,141.06 | 1,201.89 | 2367.27 | 2367.27 | 2367.27 | 2367.27 |
| Standard errors are in parentheses | | | | |  |  |  |
| *** p<.01, ** p<.05, * p<.1 | | | | |  |  |  |

- 1. **Stated preferences – quasi hyperbolic discounting**

***Table A25*** *Multinomial logistic regression with quasi-hyperbolic discounting – Anglo-Saxon and East Asian countries*

|  |  | Australia | UK | USA |  | Singapore | South Korea |
| --- | --- | --- | --- | --- | --- | --- | --- |
|  |  |  |  |  |  |  |  |
| Refuser | Rho2 | -0.44*** | -0.60*** | -0.83*** |  | -0.23 | -0.46** |
|  |  | (0.17) | (0.18) | (0.14) |  | (0.27) | (0.20) |
|  | Beta | -0.01 | 0.01 | 0.03*** |  | -0.01 | 0.03* |
|  |  | (0.02) | (0.02) | (0.01) |  | (0.04) | (0.02) |
|  | Risk in health | -0.05** | 0.01 | -0.02 |  | -0.16*** | -0.12*** |
|  |  | (0.02) | (0.02) | (0.02) |  | (0.04) | (0.02) |
|  | Female | 0.40*** | 0.15 | 0.33*** |  | 0.72*** | 0.26** |
|  |  | (0.13) | (0.12) | (0.11) |  | (0.23) | (0.12) |
|  | Age | 0.11*** | 0.12*** | 0.05** |  | 0.18*** | 0.06** |
|  |  | (0.03) | (0.03) | (0.02) |  | (0.06) | (0.03) |
|  | Age^2^ | 0*** | 0*** | 0*** |  | 0*** | 0** |
|  |  | (0) | (0) | (0) |  | (0) | (0) |
|  | Bachelor | -0.74*** | -0.45*** | -0.98*** |  | -0.34 | 0.24* |
|  |  | (0.15) | (0.14) | (0.12) |  | (0.23) | (0.15) |
|  | High income | -0.20 | -0.54** | -1.31*** |  | 0.82*** | -0.18 |
|  |  | (0.19) | (0.25) | (0.33) |  | (0.29) | (0.14) |
|  | With children | -0.02 | 0 | -0.11 |  | -0.81*** | -0.27* |
|  |  | (0.14) | (0.13) | (0.11) |  | (0.23) | (0.15) |
|  | Constant | -3.20*** | -3.16*** | -1.39*** |  | -5.43*** | -2.04*** |
|  |  | (0.66) | (0.55) | (0.43) |  | (1.36) | (0.64) |
| Hesitant | Rho2 | 0.01 | 0.29** | -0.18 |  | -0.17 | 0.22* |
|  |  | (0.12) | (0.12) | (0.12) |  | (0.20) | (0.12) |
|  | Beta | -0.03 | -0.02* | -0.01 |  | -0.01 | 0.01 |
|  |  | (0.02) | (0.01) | (0.01) |  | (0.02) | (0.01) |
|  | Risk in health | -0.03* | -0.02 | 0 |  | -0.05* | -0.06*** |
|  |  | (0.02) | (0.02) | (0.02) |  | (0.03) | (0.02) |
|  | Female | -0.01 | -0.13 | 0.09 |  | 0.06 | 0.06 |
|  |  | (0.10) | (0.10) | (0.10) |  | (0.16) | (0.09) |
|  | Age | 0.01 | -0.06*** | -0.02 |  | -0.03 | -0.07*** |
|  |  | (0.02) | (0.02) | (0.02) |  | (0.03) | (0.02) |
|  | Age^2^ | 0 | 0** | 0 |  | 0 | 0** |
|  |  | (0) | (0) | (0) |  | (0) | (0) |
|  | Bachelor | -0.16 | -0.11 | -0.40*** |  | -0.02 | 0.11 |
|  |  | (0.11) | (0.10) | (0.11) |  | (0.17) | (0.10) |
|  | High income | 0.03 | -0.07 | -0.31* |  | -0.37 | -0.06 |
|  |  | (0.14) | (0.16) | (0.18) |  | (0.26) | (0.09) |
|  | With children | -0.12 | 0.02 | -0.03 |  | 0.14 | 0.13 |
|  |  | (0.11) | (0.10) | (0.11) |  | (0.18) | (0.11) |
|  | Constant | -0.88* | 0.62* | -0.20 |  | 0.21 | 1.34*** |
|  |  | (0.46) | (0.35) | (0.38) |  | (0.76) | (0.40) |
| Vaccinated | (baseline) | - | - | - |  | - | - |
|  | Observations | 2953 | 3058 | 3004 |  | 993 | 2947 |
|  | Pseudo R2 | 0.03 | 0.04 | 0.05 |  | 0.06 | 0.03 |
|  | ll | -2230.43 | -2381.08 | -2531.82 |  | -779.68 | -2629.75 |
|  | Akaike's Crit | 4500.86 | 4802.17 | 5103.64 |  | 1599.37 | 5299.49 |
|  | Bayesian Crit | 4620.67 | 4922.68 | 5223.79 |  | 1697.38 | 5419.26 |
| Standard errors are in parentheses | | | | |  |  |  |
| *** p<.01, ** p<.05, * p<.1 | | | | |  |  |  |

**Table A26** Multinomial logistic regression on revealed preferences with quasi-hyperbolic discounting – Eastern European countries

|  |  | Croatia | Latvia | Lithuania | Slovakia | Slovenia |
| --- | --- | --- | --- | --- | --- | --- |
|  |  |  |  |  |  |  |
| Refuser | Rho2 | -0.03 | 0.01 | 0.15 | -0.20 | 0.23 |
|  |  | (0.19) | (0.18) | (0.19) | (0.21) | (0.19) |
|  | Beta | 0.01 | 0.01 | -0.01 | 0.01 | -0.01 |
|  |  | (0.01) | (0.01) | (0.01) | (0.02) | (0.02) |
|  | Risk in health | 0.07** | -0.04 | 0.02 | -0.02 | 0 |
|  |  | (0.03) | (0.03) | (0.03) | (0.03) | (0.02) |
|  | Female | 0.41*** | 0.65*** | 0.42*** | 0.20 | 0.46*** |
|  |  | (0.15) | (0.15) | (0.15) | (0.16) | (0.14) |
|  | Age | 0.06** | 0.02 | 0.08*** | 0.09*** | 0.06** |
|  |  | (0.03) | (0.03) | (0.03) | (0.03) | (0.03) |
|  | Age^2^ | 0*** | 0 | 0*** | 0*** | 0** |
|  |  | (0) | (0) | (0) | (0) | (0) |
|  | Bachelor | -0.27* | -0.53*** | -0.35** | -0.22 | -0.06 |
|  |  | (0.15) | (0.16) | (0.16) | (0.17) | (0.15) |
|  | High income | -0.02 | -0.39** | 0.02 | -0.28 | -0.07 |
|  |  | (0.20) | (0.20) | (0.29) | (0.21) | (0.18) |
|  | With children | 0.01 | -0.06 | 0.15 | 0.11 | -0.19 |
|  |  | (0.19) | (0.17) | (0.17) | (0.18) | (0.19) |
|  | Constant | -1.16* | 0.34 | -1.45** | -2.31*** | -1.44** |
|  |  | (0.66) | (0.62) | (0.64) | (0.71) | (0.62) |
| Hesitant | Rho2 | 0.16 | 0.36* | 0.11 | 0.21 | 0.34 |
|  |  | (0.21) | (0.21) | (0.23) | (0.23) | (0.22) |
|  | Beta | -0.01 | -0.01 | 0.01 | -0.01 | -0.01 |
|  |  | (0.02) | (0.02) | (0.02) | (0.02) | (0.02) |
|  | Risk in health | 0.01 | 0.07** | 0.01 | -0.03 | -0.02 |
|  |  | (0.03) | (0.03) | (0.03) | (0.03) | (0.03) |
|  | Female | -0.01 | -0.02 | 0.07 | -0.35* | 0.23 |
|  |  | (0.17) | (0.18) | (0.18) | (0.19) | (0.17) |
|  | Age | 0 | -0.05* | -0.03 | -0.03 | 0.05 |
|  |  | (0.03) | (0.03) | (0.03) | (0.03) | (0.03) |
|  | Age^2^ | 0 | 0 | 0 | 0 | 0** |
|  |  | (0) | (0) | (0) | (0) | (0) |
|  | Bachelor | -0.08 | 0.20 | 0.30 | 0.20 | -0.01 |
|  |  | (0.17) | (0.19) | (0.21) | (0.19) | (0.18) |
|  | High income | -0.10 | 0.10 | -0.16 | -0.65*** | 0.21 |
|  |  | (0.22) | (0.22) | (0.36) | (0.24) | (0.20) |
|  | With children | 0.18 | -0.26 | -0.06 | 0.25 | -0.38* |
|  |  | (0.21) | (0.21) | (0.20) | (0.21) | (0.21) |
|  | Constant | 0.09 | 0.87 | -0.05 | 0.54 | -1.23* |
|  |  | (0.70) | (0.69) | (0.69) | (0.71) | (0.70) |
| Vaccinated | (baseline) | - | - | - | - | - |
|  | Observations | 1036 | 1067 | 986 | 966 | 1042 |
|  | Pseudo R2 | 0.04 | 0.05 | 0.02 | 0.03 | 0.02 |
|  | ll | -1055.01 | -1065.16 | -1019.01 | -944.82 | -1062.54 |
|  | Akaike's Crit | 2150.02 | 2170.32 | 2078.02 | 1929.65 | 2165.07 |
|  | Bayesian Crit | 2248.88 | 2269.77 | 2175.89 | 2027.11 | 2264.05 |
| Standard errors are in parentheses | | | | |  |  |
| *** p<.01, ** p<.05, * p<.1 | | | | |  |  |

**Table A27** Multinomial logistic regression on revealed preferences with quasi-hyperbolic discounting – Western European countries

|  |  | France | Israel | Italy | Norway | Spain | Sweden |
| --- | --- | --- | --- | --- | --- | --- | --- |
|  |  |  |  |  |  |  |  |
| Refuser | Rho2 | -0.68*** | 0.73*** | 0 | 0.23 | -0.25 | -1.11*** |
|  |  | (0.13) | (0.22) | (0.14) | (0.31) | (0.16) | (0.34) |
|  | Beta | 0.02* | -0.01 | 0.01 | -0.03 | 0.02** | 0.02 |
|  |  | (0.01) | (0.02) | (0.01) | (0.05) | (0.01) | (0.03) |
|  | Risk in health | 0 | -0.04 | -0.01 | -0.08* | 0.02 | -0.01 |
|  |  | (0.02) | (0.03) | (0.02) | (0.05) | (0.02) | (0.04) |
|  | Female | 0.53*** | 0.08 | 0.31*** | 0.49** | 0.03 | -0.11 |
|  |  | (0.10) | (0.16) | (0.11) | (0.24) | (0.11) | (0.20) |
|  | Age | 0.05*** | -0.02 | 0.12*** | 0.17*** | 0.05** | 0.15*** |
|  |  | (0.02) | (0.03) | (0.03) | (0.05) | (0.02) | (0.04) |
|  | Age^2^ | 0*** | 0 | 0*** | 0*** | 0** | 0*** |
|  |  | (0) | (0) | (0) | (0) | (0) | (0) |
|  | Bachelor | -0.29** | 0.56*** | -0.26** | -0.04 | -0.12 | 0.12 |
|  |  | (0.12) | (0.16) | (0.12) | (0.24) | (0.12) | (0.19) |
|  | High income | -0.56*** | 1.28*** | -0.46*** | -0.04 | -0.17 | -0.51* |
|  |  | (0.15) | (0.35) | (0.17) | (0.64) | (0.26) | (0.29) |
|  | With children | -0.20* | -0.34* | 0.17 | -0.35 | -0.29** | -0.10 |
|  |  | (0.11) | (0.19) | (0.13) | (0.26) | (0.13) | (0.21) |
|  | Constant | -1.38*** | 0.32 | -4.16*** | -5.08*** | -2.51*** | -3.71*** |
|  |  | (0.43) | (0.66) | (0.62) | (1.15) | (0.54) | (0.96) |
| Hesitant | Rho2 | -0.02 | 0.93*** | -0.17 | 0.05 | 0.06 | 0.29* |
|  |  | (0.10) | (0.21) | (0.12) | (0.21) | (0.12) | (0.15) |
|  | Beta | 0 | -0.02 | 0 | 0.01 | -0.01 | -0.02 |
|  |  | (0.01) | (0.02) | (0.01) | (0.02) | (0.01) | (0.02) |
|  | Risk in health | -0.01 | 0.03 | 0 | -0.02 | -0.02 | -0.04 |
|  |  | (0.02) | (0.03) | (0.02) | (0.03) | (0.02) | (0.02) |
|  | Female | 0.04 | -0.11 | 0.05 | -0.34** | -0.16* | -0.15 |
|  |  | (0.09) | (0.16) | (0.09) | (0.16) | (0.10) | (0.12) |
|  | Age | -0.03* | -0.07** | 0.01 | 0.01 | -0.02 | -0.02 |
|  |  | (0.02) | (0.03) | (0.02) | (0.03) | (0.02) | (0.02) |
|  | Age^2^ | 0 | 0** | 0 | 0 | 0 | 0 |
|  |  | (0) | (0) | (0) | (0) | (0) | (0) |
|  | Bachelor | 0.04 | 0.45*** | 0.15 | 0.10 | 0.13 | 0.01 |
|  |  | (0.11) | (0.16) | (0.10) | (0.16) | (0.10) | (0.12) |
|  | High income | 0.01 | 1.16*** | -0.20 | 0.55* | 0.16 | -0.02 |
|  |  | (0.12) | (0.35) | (0.13) | (0.32) | (0.19) | (0.15) |
|  | With children | -0.06 | -0.11 | 0.14 | -0.11 | -0.17 | 0.34*** |
|  |  | (0.10) | (0.19) | (0.11) | (0.18) | (0.11) | (0.13) |
|  | Constant | 0.46 | 1.75*** | -0.99** | -0.67 | -0.40 | 0.65 |
|  | Rho2 | (0.35) | (0.63) | (0.45) | (0.60) | (0.41) | (0.48) |
| Vaccinated | (baseline) | - | - | - | - | - | - |
|  | Observations | 3116 | 1484 | 2992 | 1013 | 3240 | 1481 |
|  | Pseudo R2 | 0.03 | 0.05 | 0.02 | 0.03 | 0.01 | 0.05 |
|  | ll | -3050.44 | -1442.42 | -2601.73 | -794.19 | -2532.40 | -1287.88 |
|  | Akaike's Crit | 6140.89 | 2924.85 | 5243.47 | 1628.38 | 5104.80 | 2615.76 |
|  | Bayesian Crit | 6261.77 | 3030.90 | 5363.54 | 1726.80 | 5226.47 | 2721.77 |
| Standard errors are in parentheses | | | | |  |  |  |
| *** p<.01, ** p<.05, * p<.1 | | | | |  |  |  |

**Table A28** Multinomial logistic regression on revealed preferences with quasi-hyperbolic discounting – Emerging countries

|  |  | Brazil | Chile | India | Russia | South Africa | Turkey |
| --- | --- | --- | --- | --- | --- | --- | --- |
|  |  |  |  |  |  |  |  |
| Refuser | Average Rho | -0.10 | -0.25 | -0.48* | -0.17 | -0.41*** | 0.09 |
|  |  | (0.21) | (0.17) | (0.28) | (0.11) | (0.12) | (0.11) |
|  |  | 0.01 | 0.01 | 0.02 | 0 | 0 | -0.02* |
|  |  | (0.01) | (0.01) | (0.02) | (0.01) | (0.01) | (0.01) |
|  | Risk in health | 0.06** | 0.01 | -0.07** | -0.06*** | -0.02 | -0.10*** |
|  |  | (0.03) | (0.02) | (0.03) | (0.02) | (0.01) | (0.02) |
|  | Female | -0.49*** | 0.01 | 0.09 | 0.23** | -0.07 | -0.08 |
|  |  | (0.17) | (0.12) | (0.25) | (0.09) | (0.09) | (0.10) |
|  | Age | 0.08** | 0.06** | 0.04 | 0.10*** | 0.11*** | 0.03 |
|  |  | (0.04) | (0.03) | (0.05) | (0.02) | (0.02) | (0.02) |
|  | Age^2^ | 0 | 0*** | 0 | 0*** | 0*** | 0 |
|  |  | (0) | (0) | (0) | (0) | (0) | (0) |
|  | Bachelor | -0.01 | 0.02 | -0.03 | -0.22** | -0.32*** | -0.37*** |
|  |  | (0.18) | (0.14) | (0.32) | (0.09) | (0.10) | (0.11) |
|  | High income | -0.21 | -0.27** | -0.26 | -0.62*** | 0.07 | 0.07 |
|  |  | (0.19) | (0.13) | (0.28) | (0.12) | (0.10) | (0.11) |
|  | With children | -0.35* | 0.23 | -0.96*** | -0.39*** | -0.39*** | -0.96*** |
|  |  | (0.19) | (0.15) | (0.29) | (0.11) | (0.11) | (0.12) |
|  | Constant | -4.68*** | -2.67*** | -3.97*** | -1.70*** | -2.69*** | -1.07** |
|  |  | (0.80) | (0.55) | (1.04) | (0.46) | (0.44) | (0.44) |
| Hesitant | Average Rho | 0.26* | 0.18 | 0.13 | 0.16 | 0.12 | 0.02 |
|  |  | (0.15) | (0.15) | (0.10) | (0.12) | (0.12) | (0.11) |
|  |  | -0.01 | 0 | -0.03** | -0.01 | -0.01 | -0.01 |
|  |  | (0.01) | (0.01) | (0.01) | (0.01) | (0.01) | (0.01) |
|  | Risk in health | -0.01 | 0.01 | -0.03** | -0.01 | 0.01 | -0.06*** |
|  |  | (0.02) | (0.02) | (0.01) | (0.02) | (0.02) | (0.01) |
|  | Female | -0.17 | -0.19 | 0.08 | 0.06 | -0.09 | -0.23** |
|  |  | (0.13) | (0.12) | (0.10) | (0.10) | (0.10) | (0.09) |
|  | Age | -0.01 | -0.01 | -0.02 | 0.03 | -0.05** | -0.05** |
|  |  | (0.03) | (0.03) | (0.02) | (0.02) | (0.02) | (0.02) |
|  | Age^2^ | 0 | 0 | 0 | 0* | 0 | 0* |
|  |  | (0) | (0) | (0) | (0) | (0) | (0) |
|  | Bachelor | -0.16 | -0.04 | -0.07 | 0.02 | 0.22** | 0.14 |
|  |  | (0.15) | (0.14) | (0.13) | (0.11) | (0.11) | (0.10) |
|  | High income | -0.23 | -0.14 | -0.13 | -0.43*** | -0.17 | -0.06 |
|  |  | (0.15) | (0.13) | (0.11) | (0.12) | (0.11) | (0.10) |
|  | With children | -0.14 | 0.03 | -0.20 | -0.35*** | -0.26** | -0.33*** |
|  |  | (0.15) | (0.14) | (0.12) | (0.12) | (0.12) | (0.12) |
|  | Constant | -1.62*** | -0.77 | -0.97** | -0.52 | 0.19 | 0.75* |
|  |  | (0.54) | (0.51) | (0.40) | (0.46) | (0.43) | (0.40) |
| Vaccinated | (baseline) | - | - | - | - | - | - |
|  | Observations | 2983 | 2984 | 3097 | 2851 | 2872 | 3062 |
|  | Pseudo R2 | 0.02 | 0.03 | 0.02 | 0.02 | 0.03 | 0.04 |
|  | ll | -1485.11 | -2054.70 | -1681.00 | -2981.61 | -2676.95 | -2730.76 |
|  | Akaike's Crit | 3010.21 | 4149.41 | 3402.00 | 6003.21 | 5393.89 | 5501.53 |
|  | Bayesian Crit | 3130.23 | 4269.43 | 3522.77 | 6122.32 | 5513.15 | 5622.06 |
| Standard errors are in parentheses | | | | |  |  |  |
| *** p<.01, ** p<.05, * p<.1 | | | | |  |  |  |

1. **Data collection timeline and population quota sources**

**Table A29** Data collection period

| **Country** | **Data collection period** | | |
| --- | --- | --- | --- |
|  | Start | End | |
| Australia | 1/07/2022 | | 15/09/2022 |
| Brazil | 1/07/2022 | | 20/09/2022 |
| Brazil | 1/07/2022 | | 20/09/2022 |
| Chile | 1/07/2022 | | 10/10/2022 |
| Croatia | 1/07/2022 | | 15/09/2022 |
| France | 1/07/2022 | | 15/01/2023 |
| India | 16/12/2022 | | 30/03/2023 |
| Israel | 1/07/2022 | | 10/10/2022 |
| Italy | 1/07/2022 | | 14/09/2022 |
| Latvia | 10/10/2022 | | 6/03/2023 |
| Lithuania | 8/06/2023 | | 12/08/2023 |
| Norway | 1/07/2022 | | 21/01/2023 |
| Russia | 1/07/2022 | | 19/09/2022 |
| Singapore | 16/12/2022 | | 6/03/2023 |
| Slovakia | 1/07/2022 | | 15/09/2022 |
| Slovenia | 1/07/2022 | | 15/09/2022 |
| South Africa | 28/12/2022 | | 30/03/2023 |
| South Korea | 1/07/2022 | | 28/09/2022 |
| Spain | 1/07/2022 | | 21/01/2023 |
| Sweden | 28/12/2022 | | 7/03/2023 |
| Turkey | 16/12/2022 | | 30/03/2023 |
| UK | 1/07/2022 | | 21/01/2023 |
| USA | 1/07/2022 | | 31/01/2023 |

**Table A30** Sources for population quota (age, gender, geographical locations)

| **Country** | **Population sources** | **Data accessed** |
| --- | --- | --- |
| Australia | https://www.abs.gov.au/statistics/people/population/regional-population-age-and-sex/latest-release#data-download | 20/05/2022 |
| Brazil | [https://www.ibge.gov.br/en/statistics/social/population/18391-2010-population-census.html?=&t=resultados](https://protect-au.mimecast.com/s/xceiCp81n0U6Q27nf2jHNm?domain=ibge.gov.br) | 20/05/2022 |
| Chile | [https://stat.ine.cl/](https://protect-au.mimecast.com/s/n3w1CwV1xBfr0j2GIlooW0?domain=stat.ine.cl/) | 20/05/2022 |
| Croatia | https://web.dzs.hr/PXWeb/Menu.aspx?px_tableid=SP23_1.px&px_path=&px_language=en&px_db=Stanovni%c5%a1tvo&rxid=bc001af1-6eab-4e6c-983c-63b268b6caf9 | 18/06/2022 |
| France | https://www.insee.fr/fr/statistiques/6327222?sommaire=6327254 | 13/07/2022 |
| India | https://censusindia.gov.in/census.website/data/census-tables | 13/12/2022 |
| Israel | [https://www.cbs.gov.il/en/Pages/Social-Survey-Generator-new.aspx](https://protect-au.mimecast.com/s/Lj9xCvl1wAUVO9R7cwloXr?domain=cbs.gov.il) | 20/05/2022 |
| Italy | [https://demo.istat.it/](https://protect-au.mimecast.com/s/jegMClx10nU4Orj2i9oAhb?domain=demo.istat.it/) | 20/05/2022 |
| Latvia | https://stat.gov.lv/en/statistics-themes/population/population/tables/ird041-population-regions-cities-and-municipalities?themeCode=IR | 31/08/2022 |
| Lithuania | https://osp.stat.gov.lt/statistiniu-rodikliu-analize?hash=ab672ada-e22f-409e-9ce0-33915afade8f#/ | 5/06/2023 |
| Norway | https://www.ssb.no/en/statbank/list/folkemengde/ | 20/05/2022 |
| Russia | [https://rosstat.gov.ru/vpn_popul](https://protect-au.mimecast.com/s/U97dCq71ovtwLQV8UqtsQ8?domain=rosstat.gov.ru) | 05/06/2022 |
| Singapore | https://tablebuilder.singstat.gov.sg/table/CT/17396 | 13/12/2022 |
| Slovakia | https://data.statistics.sk/api/detail.php# | 05/06/2022 |
| Slovenia | https://pxweb.stat.si/SiStatData/pxweb/en/Data/-/05C1002S.px | 05/06/2022 |
| South Africa | :https://www.statssa.gov.za/?page_id=1854&PPN=P0302&SCH=73305 | 15/12/2022 |
| South Korea | [https://kosis.kr/eng/statisticsList/statisticsListIndex.do?menuId=M_01_01&vwcd=MT_ETITLE&parmTabId=M_01_01#content-group](https://protect-au.mimecast.com/s/LQwXCr81pwUJwqW8cQi2CR?domain=kosis.kr) | 28/05/2022 |
| Spain | <https://www.ine.es/dyngs/INEbase/en/operacion.htm?c=Estadistica_C&cid=1254736176951&menu=ultiDatos&idp=1254735572981> | 18/06/2022 |
| Sweden | https://www.statistikdatabasen.scb.se/pxweb/en/ssd/START__BE__BE0101__BE0101A/BefolkningNy/table/tableViewLayout1/ | 15/12/2022 |
| Turkey | https://data.tuik.gov.tr/Bulten/Index?p=The-Results-of-Address-Based-Population-Registration-System-2021-45500&dil=2 | 15/12/2022 |
| UK | https://www.ons.gov.uk/census | 18/06/2022 |
| US | https://www.census.gov/data/tables/2019/demo/age-and-sex/2019-age-sex-composition.html  https://www.census.gov/quickfacts/geo/chart/ID/PST045221 | 20/05/2022 |

1. **Discrete choice experiment**

**Table A31** Attributes and levels used in the DCE

|  | **Levels** | | | | |
| --- | --- | --- | --- | --- | --- |
| **Attributes** | **1** | **2** | **3** | **4** | **5** |
| **Effectiveness in reducing the risk of severe symptoms** | 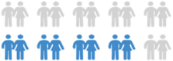  40 out of 100 will be protected | 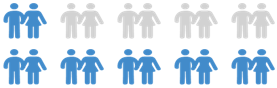  60 out of 100 will be protected | 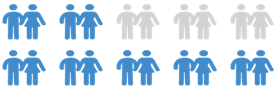  70 out of 100 will be protected | 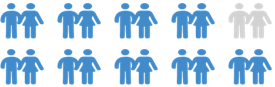  90 out of 100 will be protected |  |
| **Risk of severe side-effects after the vaccination** | 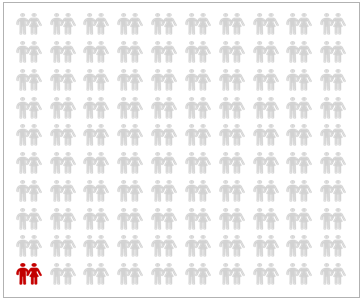  Risk of severe side-effects:  1 out of 100,000 | 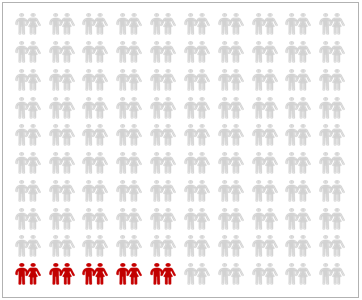  Risk of severe side-effects:  5 out of 100,000 | 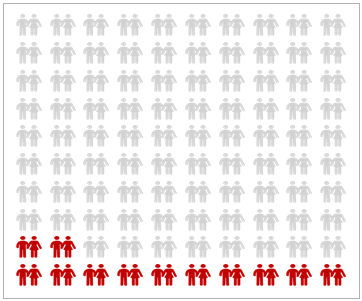  Risk of severe side-effects  12 out of 100,000 | 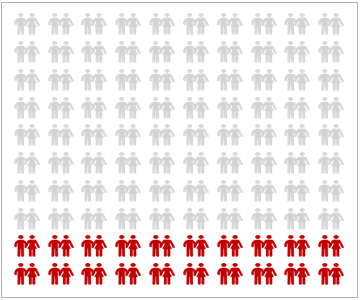  Risk of severe side-effects  20 out of 100,000 |  |
| **Duration of the protection** | 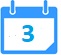  Duration of the protection:  3 months | 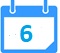 Duration of the protection:  6 months | 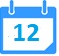  Duration of the protection:  12 months | 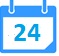  Duration of the protection:  24 months |  |
| **Time taken from first vaccine trial to market approval** | 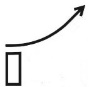  Time spent in research and development:  6 months | 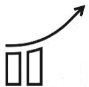 Time spent in research and development:  12 months | 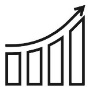  Time spent in research and development:  24 months |  |  |
| **Origin of the manufacturer** | 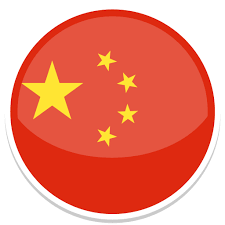  China | 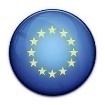  European Union | 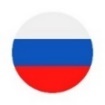  Russia | 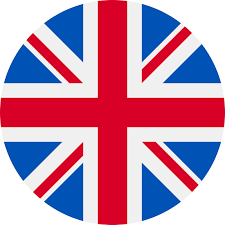  UK | 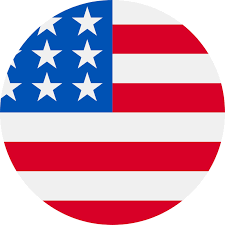  USA |
| **Social/entertainment activities of leisure** | 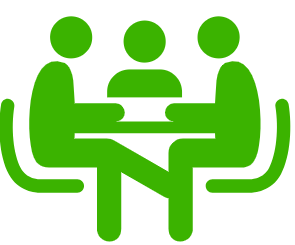  All social activities allowed | 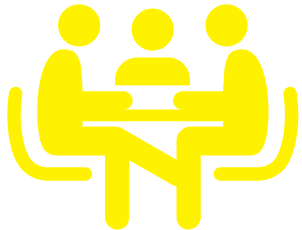  Some social activities allowed | 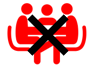  No social activities allowed |  |  |
| **Vaccination requirement to return to formal or informal work activities** | 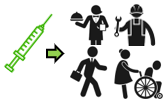  Return to formal and informal work activities allowed only with the vaccine | 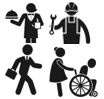  Return to formal and informal work activities allowed without the vaccine |  |  |  |

Figure A2: DCE choice task – example


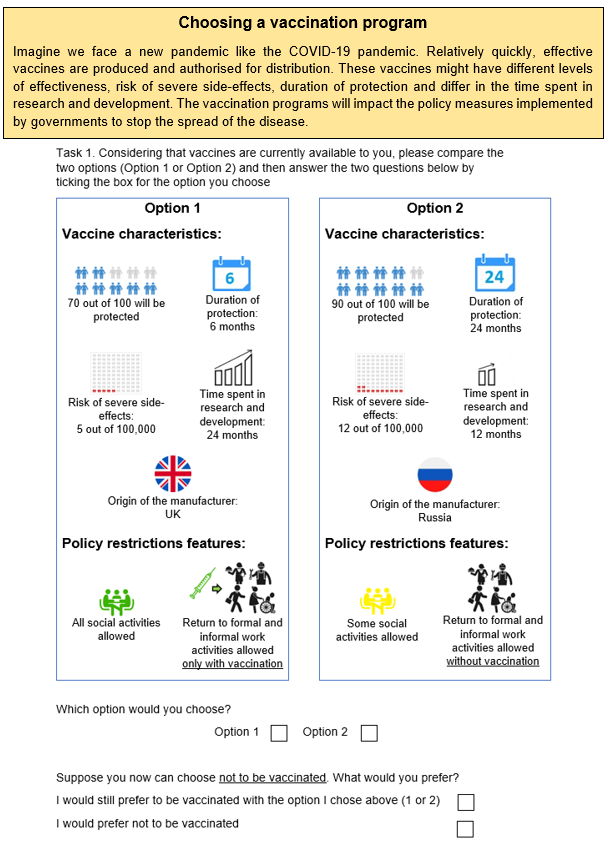


**References**

1. Antonini, M., Genie, D.M.G., Attema, A., Attwell, D.K., Balogh, P.Z.J., Behmane, D.D., Berardi, C., Brammli-Greenberg, D.S., Greenland, A., Hagen, P.T.P., Hinwood, D.M., James, P.C., Kellner, A., Kelly, P.B., Murauskienė, D.L., McGregor, D.N., Melegaro, P.A., Moy, D.N., Sequeira, D.A.R., Singh, D.R., Torbica, D.A., Ward, D.J.K., Yang, D.D., Paolucci, P.F.: Public preferences for vaccination campaigns in the COVID-19 endemic phase: Insights from the VaxPref database. Health Policy and Technology, 100849 (2024) doi: 10.1016/j.hlpt.2024.100849
